# Altered Stereostructures of the DNA-Binding Domains of Variant Mating Proteins of *Ophiocordyceps sinensis* and the Wild Insect–Fungal Complex

**DOI:** 10.3390/biology15020186

**Published:** 2026-01-19

**Authors:** Xiu-Zhang Li, Yu-Ling Li, Wei Liu, Jia-Shi Zhu

**Affiliations:** 1 State Key Laboratory of Plateau Ecology and Agriculture, Qinghai Academy of Animal and Veterinary Sciences, Qinghai University, Xining 810016, China; xiuzhang11@163.com (X.-Z.L.); yulingli2000@163.com (Y.-L.L.); 2 Institute of Immunology, Army Medical University, Chongqing 400038, China; weiliu@tmmu.edu.cn

**Keywords:** MATα_HMGbox domain of the MAT1-1-1 protein, HMG-box_ROX1-like domain of the MAT1-2-1 protein, heteromorphic protein structures, wild-type *Cordyceps sinensis* isolates, sexual reproduction of *Ophiocordyceps sinensis*, *Cordyceps sinensis* insect–fungal complex

## Abstract

The MATα_HMGbox and HMG-box_ROX1-like domains of the MAT1-1-1 and MAT1-2-1 proteins, respectively, play essential roles in DNA binding and the subsequent regulation of gene transcription, controlling *Ophiocordyceps sinensis* sexual reproduction. Alternative splicing, differential occurrence and transcription of the *MAT1-1-1* and *MAT1-2-1* genes have been demonstrated in *Hirsutella sinensis* (GC-biased Genotype #1 of the 17 *O. sinensis* genotypes), suggesting self-sterility under heterothallic or hybrid outcrossing. In this study, the MATα_HMGbox domains of MAT1-1-1 proteins in wild-type *Cordyceps sinensis* isolates were shown to cluster into 5 clades in the Bayesian clustering tree and belong to diverse stereostructure morphs under 19 AlphaFold codes. The HMG-box_ROX1-like domains of MAT1-2-1 proteins, on the other hand, were shown to cluster into 2 branched Bayesian clades and belong to stereostructure morphs under 25 AlphaFold codes. Correlation analysis revealed that 1–3 amino acid substitutions in the DNA-binding domains of the mating proteins resulted in altered hydrophobicity and secondary and tertiary structures of the DNA-binding domains of the proteins, especially altered stereostructures of the hydrophobic cores formed by 3 critical α- helices within the functional domains of the proteins. Fungal origin analysis revealed possible heterospecific fungal sources of mating proteins with stereostructure variations in wild-type *C. sinensis* isolates, suggesting that alterations in DNA binding function and the subsequent regulation of mating-related gene transcription are involved in ensuring the accuracy and genetic diversity of heterothallic and hybrid reproduction of *O. sinensis* during the lifecycle of the *C. sinensis* insect–fungal complex.

## Simple Summary

The *Cordyceps sinensis* insect–fungal complex, comprising the *Ophiocordyceps sinensis* fruiting body and the remains of a *Hepialidae* moth larva, is a highly valued therapeutic agent in traditional Chinese medicine. The sexual reproduction of *O. sinensis* is controlled by the tertiary structures of functional domains of the MAT1-1-1 and MAT1-2-1 proteins. This study reveals the primary structures of MAT1-1-1 and MAT1-2-1 protein variants derived from numerous wild-type *C. sinensis* isolates. The protein sequences exhibited various amino acid substitutions in the functional domains and clustered into several Bayesian clades associated with altered secondary and tertiary structures of the functional domains. In combination with data on the alternative splicing and differential occurrence and transcription of the *MAT1-1-1* and *MAT1-2-1* genes, the altered 3D structures of the functional domains of mating proteins refute the hypothesis that *O. sinensis* can self-fertilize via homothallic mating and instead suggest that *O. sinensis* is self-sterile and requires a mating partner for sexual reproduction under heterothallism or hybridization during the sexual life of the *C. sinensis* insect–fungal complex. This conceptual shift in reproduction mode provides a new perspective on *O. sinensis* and the *C. sinensis* insect–fungal complex and will guide future reproductive physiology studies for experimental validation.

## 1. Introduction

*Cordyceps sinensis* is one of the most expensive therapeutic agents in traditional Chinese medicine (TCM) and has a rich history of clinical use over several centuries for health maintenance, disease amelioration, post-illness and postoperative recovery, and antiaging therapy [1,2,3]. This natural therapeutic agent consists of the *Ophiocordyceps sinensis* (Hypocreales) fruiting body and the remains of a *Hepialidae* moth larva containing an intact, thick larval body wall with numerous bristles, an intact larval intestine, head tissues, and fragments of other larval tissues [4,5,6,7,8,9,10]. Studies of *C. sinensis* have demonstrated its multicellular heterokaryotic microscopic structures and genetic heterogeneity, including 17 genomically independent genotypes of *O. sinensis* fungi, >90 other fungal species spanning at least 37 fungal genera, and larval genes [4,7,10,11,12,13,14,15,16,17,18,19,20,21,22,23,24,25,26,27,28,29]. Thus, the Chinese Pharmacopoeia defines natural *C. sinensis* as an insect–fungal complex classified as a LEVEL-II endangered natural species [30].

Among the numerous fungal species [15,21,26], *Hirsutella sinensis* has been postulated to be the sole anamorph of *O. sinensis* [31]. However, ten years later, in an artificial cultivation study conducted in an industrial product-oriented setting, Wei et al. [32] reported a species contradiction between anamorphic inoculants (GC-biased Genotype #1 *H. sinensis* strains) on *Hepialidae* moth larvae and the sole teleomorph (the genomically independent AT-biased Genotype #4 of *O. sinensis*) in the fruiting body of cultivated *C. sinensis*.

Notably, the Latin name *Cordyceps sinensis*, which was originally given to the intrinsic fungus, has been indiscriminately used since the 1840s to refer to both the teleomorph/holomorph of the fungus *C. sinensis* and the wild insect–fungal complex. The fungus was renamed *O. sinensis* in 2007, with the use of the *H. sinensis* strain EFCC 7287 as the nomenclatural reference [4,7,33,34,35]. Zhang et al. [36] proposed improper implementation of the “One Fungus = One Name” nomenclature rule set by the International Mycological Association [37], disregarding the findings of multiple genomically independent genotypes of *O. sinensis* fungi and inappropriately replacing the anamorphic name *H. sinensis* with the teleomorphic name *O. sinensis* [7,9,10]. In this work, we continue to use the anamorphic name *H. sinensis* for GC-biased Genotype #1 of the 17 *O. sinensis* genotypes that may share a common evolutionary ancestor [17] and refer to the genomically independent Genotypes #2–17 fungi as *O. sinensis* according to the taxonomic descriptions that are used in several public depository databases, such as the GenBank and AlphaFold databases, before the systematic positions of these evolutionarily related genotypes are individually determined and differentiated, regardless of whether they are GC- or AT-biased genetically. In this study, we continue to use the customary name *C. sinensis* to refer to the wild or cultivated insect–fungal complex because the renaming of *C. sinensis* to *O. sinensis* in 2007 using the *H. sinensis* strain EFCC 7287 as the nomenclatural reference did not involve the indiscriminately used Latin name for the insect–fungal complex [7,33]. However, we understand that this practice will likely be revised in the future by the differential use of proprietary and exclusive Latin names for the multiple genome-independent *O. sinensis* genotypic fungi and the insect–fungal complex.

The sexual reproductive behavior of ascomycetes is strictly regulated by transcription factors encoded at the mating-type (*MAT*) locus. These transcription factors constitute the core mechanism that determines mating compatibility, regulates mating type recognition, and controls the development of fruiting bodies and reproductive structures, e.g., ascocarps and ascospores [38,39,40,41,42,43,44,45,46]. Like other fungi in Ascomycota, the reproductive behavior of *O. sinensis* relies on the synergistic interaction of 2 mating proteins, the *MAT1-1* and *MAT1-2* idiomorphs. These proteins contain 2 types of critical domains that specifically regulate the expression of genes related to sexual reproduction: (1) the mating-type alpha high mobility group box (MATα_HMGbox) domain in the MAT1-1-1 protein and (2) the high mobility group box ROX1-like (HMG-box_ROX1-like) domain in the MAT1-2-1 protein [41,47,48,49,50]. However, differential occurrence, alternative splicing, and differential transcription of mating-type and pheromone receptor genes and heteromorphic stereostructures of the entire MAT1-1-1 and MAT1-2-1 proteins in *H. sinensis* strains and wild-type *C. sinensis* isolates have been observed, invalidating the self-fertilization hypothesis under homothallism and pseudohomothallism for *H. sinensis*, which was postulated to be the sole anamorph of *O. sinensis*, and instead suggesting that the self-sterile *O. sinensis* requires sexual partners to accomplish heterothallic or hybrid reproduction within the lifecycle of the *C. sinensis* insect–fungal complex [31,50,51,52,53,54,55].

Mutations of the 2 critical DNA-binding domains of mating proteins that result in a few amino acid substitutions may cause conformational changes in the core stereostructures of the domains, thereby affecting the synergistic interaction of the mating proteins, probably through the regulation of the expression of complementary downstream genes of the 2 idiomorphic mating proteins. Our previous study [50] showed variations in the 3D structures of complete mating proteins. This study continues from our previous study, focusing on amino acid substitutions within the MATα_HMGbox and HMG-box_ROX1-like domains of the full-length MAT1-1-1 and MAT1-2-1 proteins, respectively, and the impact of the variable primary structures of the DNA-binding domains on the changes in the hydrophobic properties and the secondary and tertiary structures of the functional domains in wild-type *C. sinensis* isolates. Correlations between changes in hydrophobicity and the primary and secondary structures of the DNA-binding domains of mating proteins encoded by the genome, transcriptome and metatranscriptome assemblies of *H. sinensis* and *C. sinensis* insect–fungal complexes were also analyzed.

## 2. Materials and Methods

### 2.1. The MAT1-1-1 and MAT1-2-1 Protein Sequences of the Wild-Type C. sinensis Isolates

The AlphaFold database lists the AlphaFold-predicted 3D structures for 138 MAT1-1-1 proteins and 74 MAT1-2-1 proteins [50]. These proteins were produced by different sample sources: *O. sinensis* strains of different genotypes (including GC-biased Genotype #1 *H. sinensis* strains), wild-type *C. sinensis* isolates, and *C. sinensis* insect-fungal complexes (Table S1). Among these proteins, 118 MAT1-1-1 proteins and 69 MAT1-2-1 proteins are full-length proteins, as shown in Tables S2 and S3. Three full-length MAT1-1-1 proteins (ALH24945, AGW27560, and EQK97643) and 2 full-length MAT1-2-1 proteins (AEH27625 and EQL04085) were derived from *H. sinensis* strains (bolded and underlined in Tables S2 and S3). All other full-length mating proteins were derived from wild-type *C. sinensis* isolates that were collected from various production areas on the Qinghai–Tibet Plateau [5,34,36,49,50,52,56,57]. The internal transcribed spacer (ITS) sequence information is available in the GenBank database for the full-length MAT1-1-1 and MAT1-2-1 protein variants derived from 34 wild-type *C. sinensis* isolates and aligned with the reference ITS sequences of the 7 GC-biased genotypes of *O. sinensis* (Table S4).

The remaining 20 MAT1-1-1 proteins (AGW27517–AGW27536) and 5 MAT1-2-1 proteins (AGW27543, AGW27548, AGW27552, AGW27554, and AGW27555), which were derived from *O. sinensis* strains [23,48], are truncated at the N- and/or C-termini and will be analyzed elsewhere.

### 2.2. Genome, Transcriptome, and Metatranscriptome Assemblies of H. sinensis Strains and C. sinensis Insect–Fungal Complexes

The GenBank database lists 5 genome assemblies, LKHE00000000, NGJJ00000000, ANOV00000000, JAAVMX000000000, and LWBQ00000000, of the *H. sinensis* strains 1229, CC1406-20395, Co18, IOZ07, and ZJB12195, respectively, and a transcriptome assembly, GCQL00000000, of the *H. sinensis* strain L0106 [49,58,59,60,61,62].

The metatranscriptome assembly GAGW00000000 for the *C. sinensis* insect–fungal samples (unknown maturation stages) collected from Kangding County, Sichuan Province, China [63], is available in the GenBank database.

Another metatranscriptome assembly was derived from mature *C. sinensis* samples collected from Deqin, Yunnan Province, China, and was uploaded to a repository database, http://www.plantkingdomgdb.com/Ophiocordyceps_sinensis/ (accessed from 18 May 2017) [64]. Although this database is currently inaccessible, it was accessed from 18 May 2017 to 18 January 2018, and a cDNA file was downloaded.

The genome, transcriptome, and metatranscriptome assemblies were used to analyze the MATα_HMGbox and HMG-box_ROX1-like domains of the MAT1-1-1 and MAT1-2-1 proteins, respectively.

### 2.3. Alignment of the DNA-Binding Domain Sequences of the Mating Proteins

The amino acid sequences of the MATα_HMGbox domains of the MAT1-1-1 proteins and the HMG-box_ROX1-like domains of the MAT1-2-1 proteins of the wild-type *C. sinensis* isolates and *C. sinensis* insect–fungal complexes were aligned using the GenBank Blastp program (https://blast.ncbi.nlm.nih.gov/ (Bethesda, MD, USA), accessed from 18 October 2024 to 10 July 2025).

### 2.4. Bayesian Clustering Analysis of the DNA-Binding Domains of the Mating Proteins

Multiple sequence alignment of the DNA-binding domains of the mating proteins of the wild-type *C. sinensis* isolates and *C. sinensis* insect–fungal complexes was performed using the auto mode of MAFFT (v7.427; Osaka, Japan). The intron regions were removed using trimAl (v1.4. rev15; Barcelona, Spain). Based on the Bayesian information criterion, ProtTest v3.4.2 (University of Vigo and University of La Coruña, Spain) was used to determine the optimal amino acid substitution model. Bayesian clustering trees of the DNA-binding domain sequences of the MAT1-1-1 and MAT1-2-1 proteins were then inferred using MrBayes v3.2.7 software (Markov chain Monte Carlo [MCMC] algorithm; Uppsala, Sweden) at a sampling frequency of 100 iterations after discarding the initial 25% of the samples from a total of 1 million iterations. This process yielded final majority rule consensus trees for the MATα_HMGbox domains of the MAT1-1-1 proteins and the HMG-box_ROX1-like domains of the MAT1-2-1 proteins [7,50,54,55,65,66]. Clustering analysis was conducted by Nanjing Genepioneer Biotechnologies Co. (Nanjing, China).

### 2.5. Amino Acid Properties and Scale Analysis

The amino acid sequences of the DNA-binding domains of the mating proteins were scaled on the basis of the general chemical characteristics of their side chains (Table S5), as reported at https://web.expasy.org/protscale/ (Basel, Switzerland), accessed from 18 October 2024 to 20 May 2025 [50,55,67,68,69,70,71]. The MATα_HMGbox domain of MAT1-1-1 proteins (amino acids 51→225 of the reference sequence AGW27560 derived from the *H. sinensis* strain CS68-2-1229 (Table S2) [48]) and the HMG-box_ROX1-like domain of MAT1-2-1 proteins (amino acids 127→197 of the reference sequence AEH27625 derived from the *H. sinensis* strain CS2 (Table S3) [56]), with an additional 9 amino acid residues extending upstream and downstream of the domains, were plotted sequentially with a window size of 9 amino acid residues using the linear weight variation model of the ExPASy ProtScale algorithms [50,55,67,68,69,70,71] to generate ExPASy ProtScale plots that display and compare the topology and waveform changes in hydrophobicity and the 2D structures for α-helices, β-sheets, β-turns, and coils of the DNA-binding domains of the mating proteins.

### 2.6. AlphaFold-Based Predictions of the 3D Structures of the Mating Proteins

For heteromorphic stereostructure analysis, the 3D structures of the MAT1-1-1 and MAT1-2-1 proteins from the wild-type *C. sinensis* isolates were computationally predicted from their amino acid sequences using the artificial intelligence (AI)-based machine learning technology AlphaFold (https://alphafold.com/, Cambridgeshire, UK), which was downloaded from the AlphaFold database (accessed from 18 October 2024 to 10 November 2025) [50,72,73,74,75,76,77,78,79,80,81,82].

The AlphaFold database provides per-residue model confidence, predicts scores between 0 and 100 for the local distance difference test (pLDDT), and provides a per-residue score that is assigned to each individual residue [73,74,75,76,78,79]. The model’s confidence bands are used to color-code the residues in the 3D structures: residues with very high confidence (pLDDT > 90) are shown in dark blue, those with high confidence (90 > pLDDT > 70) are shown in light blue, residues with low confidence (70 > pLDDT > 50) are shown in yellow, and residues with very low confidence (pLDDT < 50) are shown in orange [50,80,83]. The AlphaFold database provides an average pLDDT score for each of the AI-predicted 3D structural models.

The main reasons for low-confidence region AlphaFold predictions include the following: (1) There are insufficient supporting data because the AlphaFold-based prediction relies on the quality of multiple sequence alignment (MSA). If few homologous sequences are available in a certain region of the MSA, the model will lack sufficient evolutionary information to infer the structure, resulting in low confidence. (2) Some protein regions are naturally flexible under physiological conditions, such as the activation domains of transcription factors, which do not have a fixed 3D structure, leading to low prediction confidence assigned by AlphaFold. (3) In terms of special structural regions, such as small-molecule binding sites and artificial linkers in fused proteins, the AlphaFold system may have limited prediction power, leading to low confidence in the prediction of complex structures. Thus, the low-confidence regions in AlphaFold predictions are informative flags for highlighting prediction challenges and should not be interpreted too confidently.

### 2.7. Correlations of the Primary, Secondary, and Tertiary Structures of the DNA-Binding Domains of the MAT1-1-1 and MAT1-2-1 Proteins

The AlphaFold-predicted 3D structures of the MATα_HMGbox domains of the MAT1-1-1 proteins and the HMG-box_ROX1-like domains of the MAT1-2-1 proteins were magnified at the sites of amino acid substitutions. The locally magnified 3D structures at the mutation sites were correlated with the amino acid substitutions to obtain the primary structures and the changes in topology and waveform via ExPASy ProtScale plotting, which revealed hydropathy, α-helices, β-sheets, β-turns, and coils for altered hydrophobicity and the 2D structures of the DNA-binding domains of the MAT1-1-1 and MAT1-2-1 proteins.

## 3. Results

### 3.1. Primary Structures of the MATα_HMGbox Domains of the Full-Length MAT1-1-1 Proteins

Among the 138 MAT1-1-1 proteins in the AlphaFold database, 118 (85.5%) are full-length proteins containing 372 amino acid residues. These proteins contribute to 15 heteromorphic 3D structure morphs belonging to 5 Bayesian clusters (Table S2) [50]. Twenty-nine (24.6%) of the 118 full-length MAT1-1-1 proteins derived from wild-type *C. sinensis* isolates contain variable amino acid substitutions.

The MAT1-1-1 proteins contain a MATα_HMGbox domain that is located at amino acids 51→225 (shown in blue and underlined in Figure S1), as represented by the reference MAT1-1-1 protein AGW27560 derived from the *H. sinensis* strain CS68-2-1229 (Table S2) [48]. Other MAT1-1-1 proteins derived from wild-type *C. sinensis* isolates or encoded by the genome assemblies of the *H. sinensis* strains and the metatranscriptome assemblies of *C. sinensis* insect–fungal complexes contain various amino acid substitutions (Figure S1). The MATα_HMGbox domains of 10 (34.5%) of the 29 variant proteins are 100% identical to the domain sequence of the query protein AGW27560 when the amino acid substitutions outside the domains are not considered. Each of the remaining 19 proteins (65.5%) contains a mutation or mutations that results in 1 or 2 amino acid substitutions at various sites within the MATα_HMGbox domains, as shown in red in Figure S1.

Figure S1 also shows the MATα_HMGbox domains of the MAT1-1-1 proteins encoded by the genome assemblies ANOV01017390 (794←1129 & 1280←1396), LKHE01001116 (4183←4620 & 4667←4759), and JAAVMX010000001 (6,699,061→6,699,153 & 6,699,203→6,699,637) of the *H. sinensis* strains Co18, 1229, and IOZ07, respectively [49,60,62]. (Note: the arrows “→” and “←” indicate sequences in the sense and antisense strands of the genomes, respectively; “&” refers to the removed intron portion.) These genome-encoded MAT1-1-1 proteins are truncated at their C-termini; the truncated segments are located far downstream of the MATα_HMGbox domains.

The genome assemblies LWBQ00000000 and NGJJ00000000 and the transcriptome assembly GCQL00000000 of the *H. sinensis* strains ZJB12195, CC1406-20395, and L0106, respectively, do not contain the genes or transcripts that encode the MAT1-1-1 proteins [58,59,60].

The metatranscriptome assemblies GAGW01008880 (300←1127) and OSIN7648 (1→1065) of natural *C. sinensis* insect–fungal complexes encode MAT1-1-1 proteins (Figure S1) [63,64]. The MAT1-1-1 protein OSIN7648 contains an 18 amino acid truncation in the middle sequence (SMQREYQAPRFFYDYSVS) between residues 863 and 864; the truncated segment is located downstream of the MATα_HMGbox domain (151→675). The N-terminus of the MATα_HMGbox domain (741←1127) of the MAT1-1-1 protein encoded by GAGW01008880 is truncated by 46 amino acid residues (AAASRATRQTKEASCDRAKRPLNAFMAFRSYYLKLFPDVQQKTASG).

### 3.2. Primary Structures of the HMG-Box_ROX1-like Domains of the Full-Length MAT1-2-1 Proteins

Among the 74 MAT1-2-1 proteins listed in the AlphaFold database, 69 (93.2%) are full-length proteins, each of which contains 249 amino acid residues; these proteins contribute to 17 heteromorphic AlphaFold tertiary structure morphs belonging to 5 Bayesian clusters (Table S3) [50]. Among the 69 full-length proteins, 39 (56.5%) are 100% identical to the representative MAT1-2-1 protein AEH27625, which was derived from the *H. sinensis* strain CS2 (Table S3) [56]. The remaining 30 full-length proteins (43.5%) contain amino acid substitutions at various sites, as shown in red in Figure S2. The HMG-box_ROX1-like domains of the MAT1-2-1 proteins are located at amino acid residues 127→197, as shown in blue and underlined in Figure S2, whereas the 9 external amino acid residues upstream and downstream of the domain are shown in blue but not underlined. The HMG-box_ROX1-like domain sequences of 3 of the 30 variable MAT1-2-1 proteins are 100% identical to the domain sequence of the query protein AEH27625, although the amino acid substitutions occur outside the functional domains, whereas the domains of the remaining 27 proteins contain 1–3 amino acid substitutions at various sites inside and/or outside the functional domains, as shown in red in Figure S2.

The MAT1-2-1 proteins encoded by the genome assemblies ANOV01000063, LKHE01001605, LWBQ01000021, and NGJJ01000619 of the *H. sinensis* strains Co18, 1229, ZJB12195, and CC1406-20395, respectively, are shown in Figure S2 [49,59,60,61]. An S-to-A substitution occurred in each of the HMG-box_ROX1-like domains of the genome-encoded MAT1-2-1 proteins ANOV01000063 (9759→9851 & 9907→10,026), LKHE01001605 (14,016←14,135 & 14,191←14,283), LWBQ01000021 (239,029←239,148 & 239,204←239,269), and NGJJ01000619 (23,186←23,305 & 23,361←23,453). An additional Y-to-H substitution occurred in the HMG-box_ROX1-like domains of LKHE01001605, LWBQ01000021 and NGJJ01000619 but not in the HMG-box_ROX1-like domain of ANOV01000063, resulting in 97.2–98.6% similarity to the sequence of the HMG-box_ROX1-like domain of the query protein AEH27625 [56].

The genome assembly JAAVMX000000000 of the *H. sinensis* strain IOZ07 and the metatranscriptome assembly GAGW00000000 of the *C. sinensis* insect–fungal complex do not contain genes or transcripts encoding MAT1-2-1 proteins [62,63].

The protein sequences encoded by the MAT1-2-1 transcripts of the transcriptome assembly GCQL01020543 (397←1143) of the *H. sinensis* strain L0106 and the metatranscriptome assembly OSIN7649 (1→747) of the mature *C. sinensis* insect–fungal complex are shown in Figure S2 [59,64]. The HMG-box_ROX1-like domains (553←765 of GCQL01020543 and 379→591 of OSIN7649) of these proteins contain Y-to-H substitutions, resulting in 98.6% similarity to the sequence of that domain in the query protein AEH27625 [36].

### 3.3. Bayesian Analysis of the MATα_HMGbox Domains of the Full-Length MAT1-1-1 Proteins

**Figure 1:**
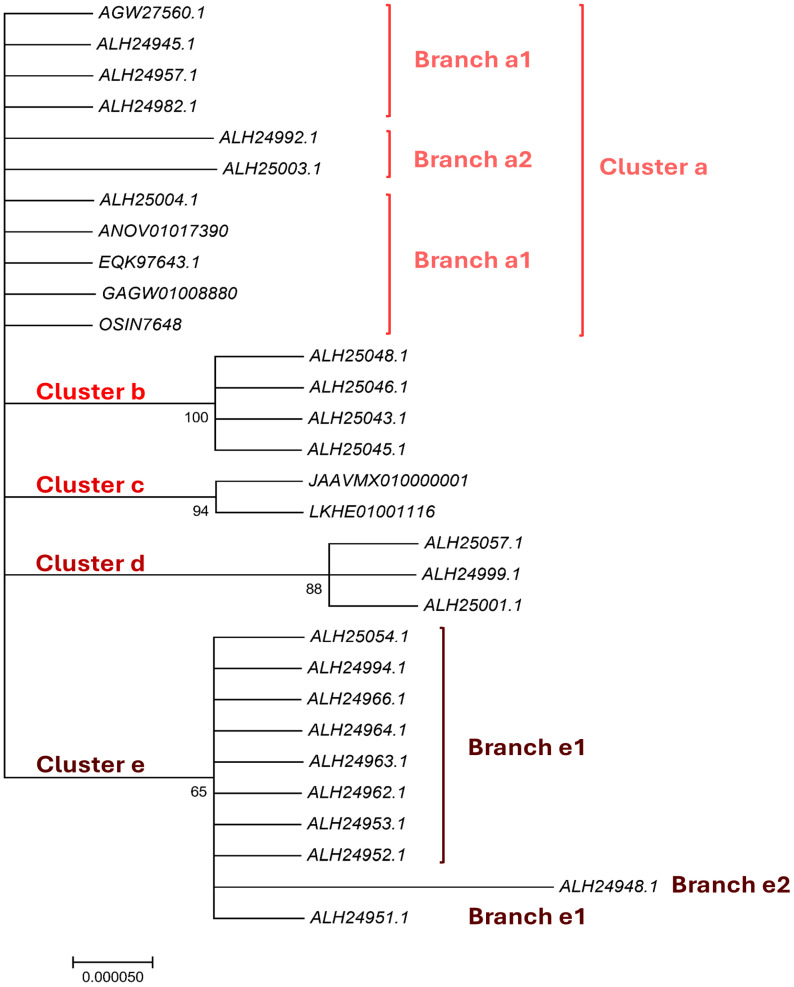
A Bayesian majority rule consensus clustering tree inferred using MrBayes v3.2.7 software for the MATα_HMGbox domain sequences of the 25 full-length MAT1-1-1 proteins of the wild-type *C. sinensis* isolates and the corresponding domain sequences encoded by the genome and metatranscriptome assemblies of the *H. sinensis* strains and *C. sinensis* insect–fungal complexes.

The sequences of the MATα_HMGbox domains of 19 full-length MAT1-1-1 proteins that display various amino acid substitutions were subjected to Bayesian clustering analysis and compared with the sequences of 6 authentic MAT1-1-1 proteins as reference sequences (Figure 1). The MATα_HMGbox domains of the full-length MAT1-1-1 proteins were clustered into 5 Bayesian clusters (Figure 1), among which Clusters a and d were branched. The functional domains of the 19 proteins with various amino acid substitutions were clustered into Clusters/Branches a2, b, d, and e1–e2 in the Bayesian clustering tree. Cluster d has a longer clustering distance than some of the other clusters, and Branch e2 has the longest clustering distance.

The MATα_HMGbox domains encoded by the genome assemblies JAAVMX010000001 (6,699,061→6,699,153 & 6,699,203→6,699,637) and LKHE01001116 (4183←4620 & 4667←4759) of the *H. sinensis* strains 1229 and IOZ07 contain Y-to-M substitutions (Figure S1) [60,62]. The domains encoded by the genome assembly ANOV01017390 (794←1129 & 1280←1396) of *H. sinensis* strain Co18 and the metatranscriptome assembly OSIN7648 (151→675) of the *C. sinensis* insect–fungal complex contain no altered amino acid residues [49,64]. The domain encoded by the metatranscriptome assembly GAGW01008880 (714←1127) of the *C. sinensis* insect–fungal complex is truncated at the N-terminus (Figure S1) [63]. The MATα_HMGbox domains encoded by the genome assembly ANOV01017390 and metatranscriptome assemblies OSIN7648 and GAGW01008880 cluster into Branch a1 in the Bayesian clustering tree, together with the 6 authentic MAT1-1-1 proteins (Figure 1).

Tables S6 and S7 summarize the amino acid substitutions and deletions in the MATα_HMGbox domains of the MAT1-1-1 proteins derived from wild-type *C. sinensis* isolates and encoded by the genome and metatranscriptome assemblies of *H. sinensis* strains and *C. sinensis* insect–fungal complexes, respectively, which resulted in different Bayesian clustering results and changes in the predicted 3D structures under various AlphaFold codes.

### 3.4. Bayesian Analysis of the HMG-Box_ROX1-like Domains of the Full-Length MAT1-2-1 Protein Sequences

**Figure 2:**
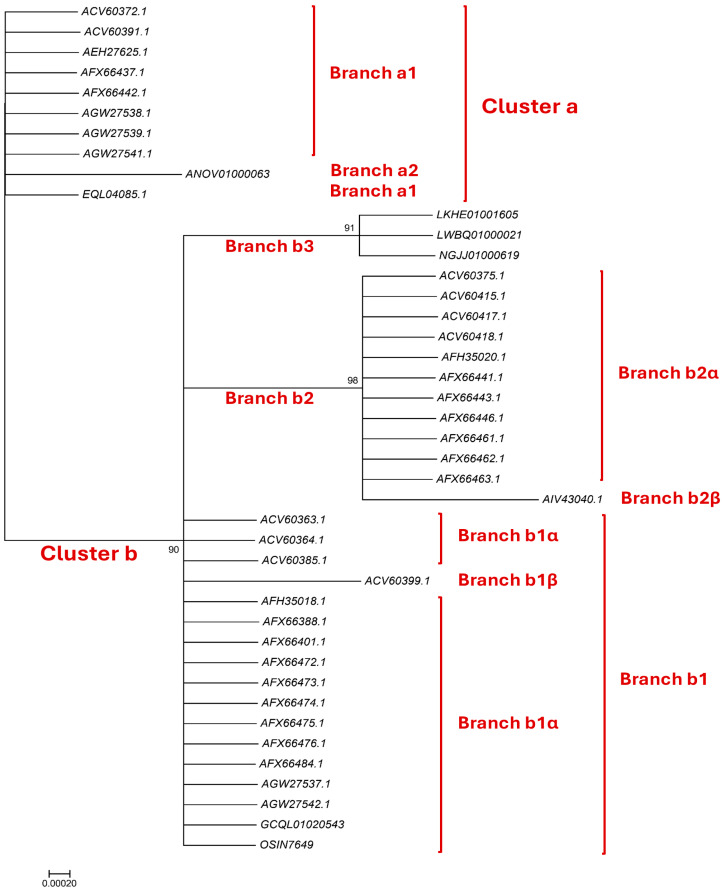
Bayesian majority rule consensus clustering tree inferred using MrBayes v3.2.7 software for the HMG-box_ROX1-like domain sequences of 35 full-length MAT1-2-1 proteins of the wild-type *C. sinensis* isolates and the corresponding domain segments of the translated genome, transcriptome, and metatranscriptome assemblies of the *H. sinensis* strains and *C. sinensis* insect–fungal complexes.

Thirty full-length MAT1-2-1 proteins that are present in wild-type *C. sinensis* isolates or encoded by the genome and transcriptome assemblies of *H. sinensis* strains and by the metatranscriptome assembly of the *C. sinensis* insect–fungal complex and that have various amino acid substitutions in their HMG-box_ROX1-like domains were subjected to Bayesian clustering analysis and compared with the HMG-box_ROX1-like domains of 6 authentic MAT1-2-1 proteins as references. Figure 2 shows 2 branched clusters (a and b). Branches b2–b3 have longer clustering distances than some of the other branches, and Branch b2β has the longest clustering distance.

The HMG-box_ROX1-like domain encoded by the genome assembly ANOV01000063 (9759→9851 & 9907→10,026) of *H. sinensis* strain Co18 contains an S-to-A substitution and is clustered into Branch a2 with a slightly longer clustering distance (Figure 2) [49]. The domains encoded by other genome assemblies [LKHE01001605 (14,016←14,135 & 14,191←14,135 & 14,191←14,283), LWBQ01000021 (239,029←239,148 & 239,204←239,269), and NGJJ01000619 (23,186←23,305 & 23,361←23,453) of the *H. sinensis* strains 1229, ZJB12195, and CC1406-20395, respectively] [59,60,61] contain Y-to-H and S-to-A substitutions and are clustered into Branch b3 in the Bayesian clustering tree (Figure 2). The HMG-box_ROX1-like domain of the MAT1-2-1 protein encoded by the transcriptome assembly GCQL01020543 (553←765) of the *H. sinensis* strain L0106 contains a Y-to-H substitution [58]. The domain of the MAT1-2-1 protein encoded by the metatranscriptome assembly OSIN7649 (379→591) of the *C. sinensis* insect–fungal complex is 100% identical to that of the reference MAT1-2-1 protein AEH27625 [56,64]. The domains of the MAT1-2-1 proteins encoded by the transcriptome and metatranscriptome assemblies were clustered into Branch b1α in the Bayesian clustering tree (Figure 2).

Tables S8 and S9 summarize the amino acid substitutions and deletions in the HMG-box_ROX1-like domains of MAT1-2-1 proteins derived from wild-type *C. sinensis* isolates and encoded by the genome, transcriptome, and metatranscriptome assemblies of *H. sinensis* strains and *C. sinensis* insect–fungal complexes, respectively, which resulted in different Bayesian clustering results and changes in the predicted 3D structures under various AlphaFold codes.

### 3.5. Heteromorphic Stereostructures of the MATα_HMGbox Domains of Full-Length MAT1-1-1 Proteins Derived from Wild-Type C. sinensis Isolates

Section 3.5 presents analyses of the correlations between the changes in the structure and hydrophobicity of the MATα_HMGbox domains of full-length MAT1-1-1 proteins (under 7 AlphaFold 3D structure codes; Table S6) derived from wild-type *C. sinensis* isolates compared with the reference MAT1-1-1 proteins represented by AGW27560 (under AlphaFold code U3N942) derived from the *H. sinensis* strain CS68-2-1229 (Table S2) [48]. The MATα_HMGbox domain sequences (amino acid residues 51→225) were extended by 9 amino acid residues both upstream and downstream of the domains because of the setting of the sequential ExPASy ProtScale plotting for hydropathy, α-helices, β-sheets, β-turns, and coils at a window size of 9 amino acid residues (Section 2.5 of the Materials and Methods).

**Figure 3:**
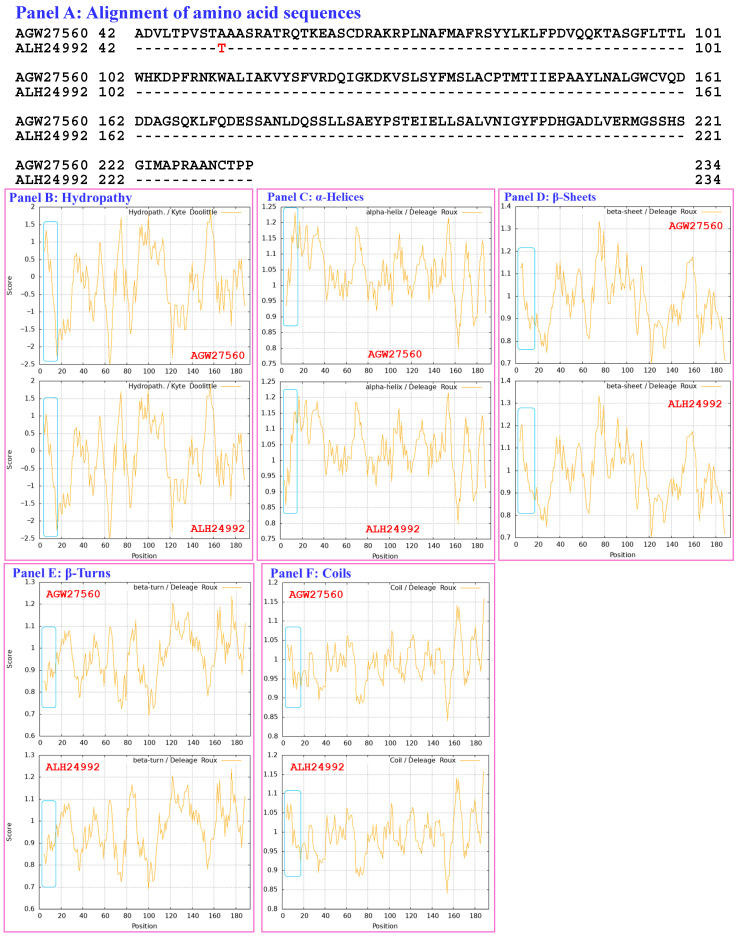

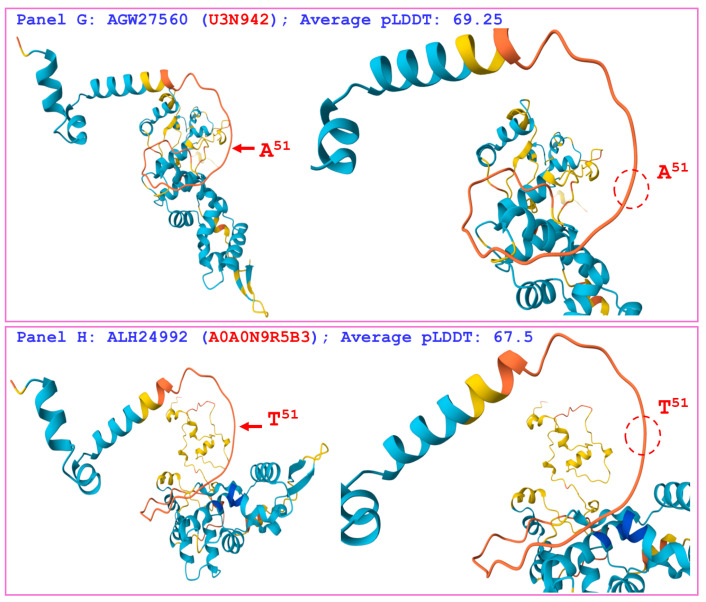
Correlations of the changes in hydrophobicity and the primary, secondary, and tertiary structures of the MATα_HMGbox domains of the reference MAT1-1-1 protein AGW27560 derived from the *H. sinensis* strain CS68-2-1229 [48] and the variant MAT1-1-1 protein ALH24992 derived from the wild-type *C. sinensis* isolate SC09_65. Panel (**A**) shows the alignment of the amino acid sequences of the MATα_HMGbox domains of the proteins; the amino acid substitution is shown in red, whereas the hyphens indicate identical amino acid residues. The ExPASy ProtScale plots show the changes in hydrophobicity (Panel (**B**)) and in the 2D structures (Panels (**C**–**F**) for α-helices, β-sheets, β-turns, and coils, respectively) of the proteins; the open blue rectangles highlight the topological structure and waveform changes of the ExPASy plots. Panels (**G**,**H**) show the 3D structures of the full-length proteins on the left and the locally magnified structures surrounding the mutation site on the right. The model confidence values for the AlphaFold-predicted 3D structures are as follows: ☐ very high (pLDDT > 90); ☐ high (90 > pLDDT > 70); ☐ low (70 > pLDDT > 50); and ☐ very low (pLDDT < 50).

Figure 3 compares the hydrophobicity and structure of the MATα_HMGbox domain of the mutant MAT1-1-1 protein ALH24992 (under AlphaFold code A0A0N9R5B3) derived from the wild-type *C. sinensis* isolate SC09_65 (Tables S2 and S6) [52] with those of the MATα_HMGbox domain of the reference protein AGW27560 (under AlphaFold code U3N942) derived from the *H. sinensis* strain CS68-2-1229 (Table S2) [48]. Panel (A) of Figure 3 shows an A-to-T substitution (aliphatic alanine to threonine with polar side chains; hydropathy indices changed from 1.8 to −0.7). This substitution reduces the hydrophobicity (the larger the hydropathy value is, the stronger the hydrophobicity, whereas negative values indicate hydrophilicity [67]), as illustrated by the topological structure and waveform changes in the hydropathy plot shown in Panel (B). These changes alter the secondary structure of the protein in the area surrounding the mutation site in the MATα_HMGbox domain of the mutant MAT1-1-1 protein ALH24992, as illustrated by the changes in the topology and waveform in the ExPASy ProtScale plots for α-helices, β-sheets, β-turns, and coils shown in Panels C–F, respectively.

As shown in Panels (G and H) of Figure 3, which illustrate the 3D structures of the proteins, the substitution is located upstream of the core structure of 3 α-helices and may not directly participate in stabilizing the hydrophobic core structure of the DNA-binding domain [84,85]. However, the replacement of alanine with a threonine residue with reduced hydrophobicity altered the stereostructure of the MATα_HMGbox domain of the MAT1-1-1 protein ALH24992 under AlphaFold code A0A0N9R5B3, possibly through long-range spatial interactions. The variant MATα_HMGbox domain clustered into Branch a2 in the Bayesian clustering tree (Figure 1, Table S6).

**Figure 4:**
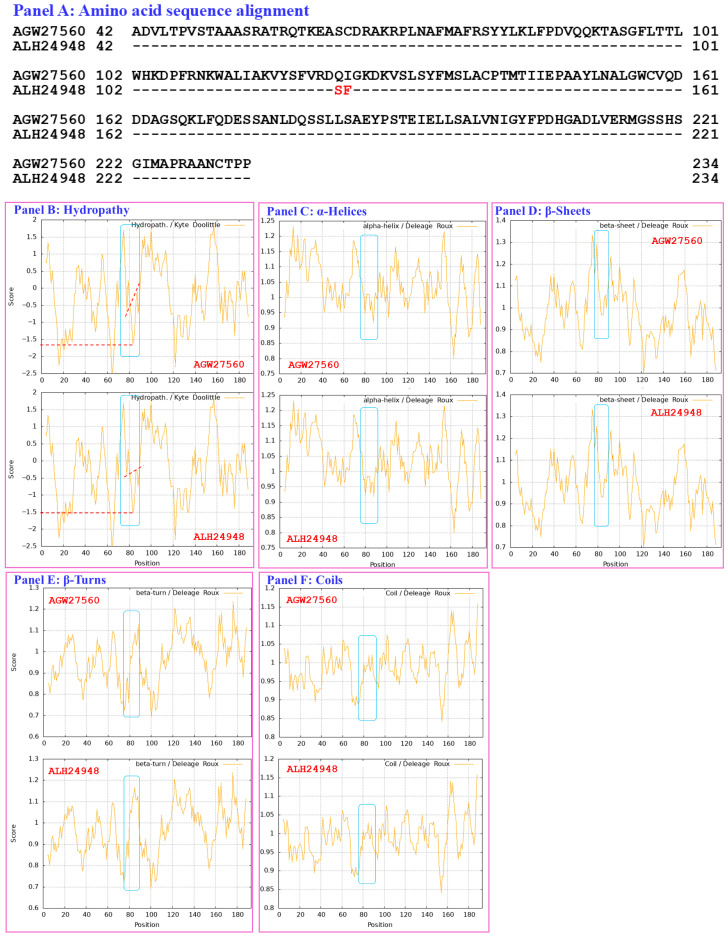

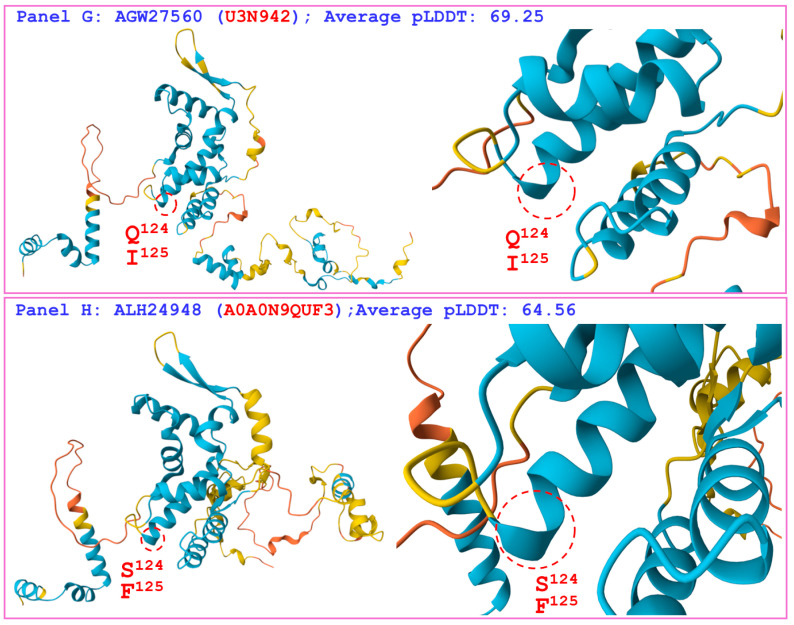
Correlations among the changes in the primary, secondary, and tertiary structures of the MATα_HMGbox domains of the reference MAT1-1-1 protein AGW27560 derived from the *H. sinensis* strain CS68-2-1229 [48] and the variant MAT1-1-1 protein ALH24948 derived from the wild-type *C. sinensis* isolate GS09_143. Panel (**A**) shows an alignment of the MATα_HMGbox domain sequences of the proteins; amino acid substitutions are shown in red, whereas the hyphens indicate identical amino acid residues. The ExPASy ProtScale plots show the changes in hydrophobicity (Panel (**B**)) and in the 2D structures (Panels (**C**–**F**) for α-helices, β-sheets, β-turns, and coils, respectively) of the proteins; the open blue rectangles highlight the topological structure and waveform changes shown in the ExPASy plots. Panels (**G**,**H**) show the 3D structures of the full-length proteins on the left and the locally magnified structures at the substitution sites on the right. The model confidence values for the AlphaFold-predicted 3D structures are as follows: ☐ very high (pLDDT > 90); ☐ high (90 > pLDDT > 70); ☐ low (70 > pLDDT > 50); and ☐ very low (pLDDT < 50).

Figure 4 compares the hydrophobicity and structure of the MATα_HMGbox domain of the MAT1-1-1 protein ALH24948 (AlphaFold code A0A0N9QUF3) derived from the wild-type *C. sinensis* isolate GS09_143 (Table S2) [52] with those of the MATα_HMGbox domain of the reference protein AGW27560 (AlphaFold code U3N942) derived from the *H. sinensis* strain CS68-2-1229 (Table S2) [48]. Panel (A) of Figure 4 shows substitutions of two adjacent residues, Q-to-S (glutamine to serine, both of which have polar side chains; hydropathy indices changed from −3.5 to −0.8 [67]) and I-to-F (aliphatic isoleucine to aromatic phenylalanine; hydropathy indices changed from 4.5 to 2.8), causing bidirectionally changed hydrophobicity, as illustrated by the topological structure and waveform changes in the hydropathy plot in Panel (B). These changes alter the secondary structure surrounding the mutation sites in the MATα_HMGbox domain, as illustrated by the topological structure and waveform changes in the ExPASy ProtScale plots for α-helices, β-sheets, β-turns, and coils shown in Panels (C–F). As shown in the diagrams of the 3D structures in Panels (G and H), the adjacent amino acid substitutions are located at the end of one of the 3 α-helices that form the hydrophobic core stereostructure [84,85], and they alter the tertiary structure of the MATα_HMGbox domain of the variant protein ALH24948 under AlphaFold code A0A0N9QUF3. The variant MATα_HMGbox domain clustered into Branch e2 in the Bayesian clustering tree (Figure 1, Table S6).

**Figure 5:**
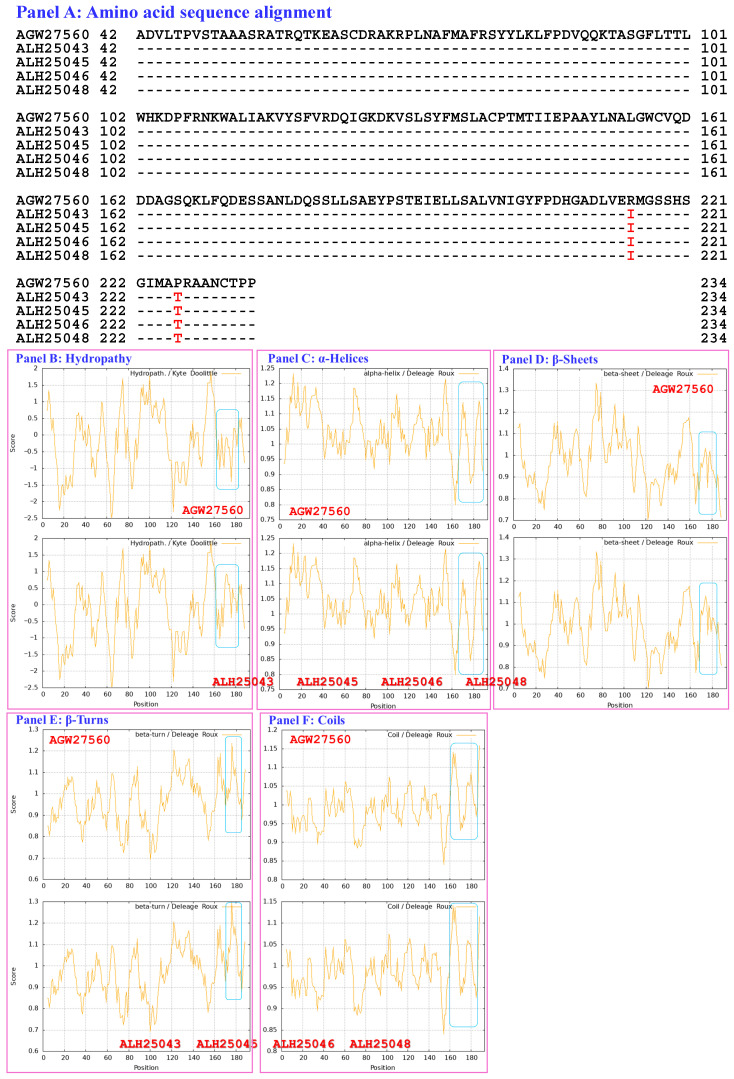

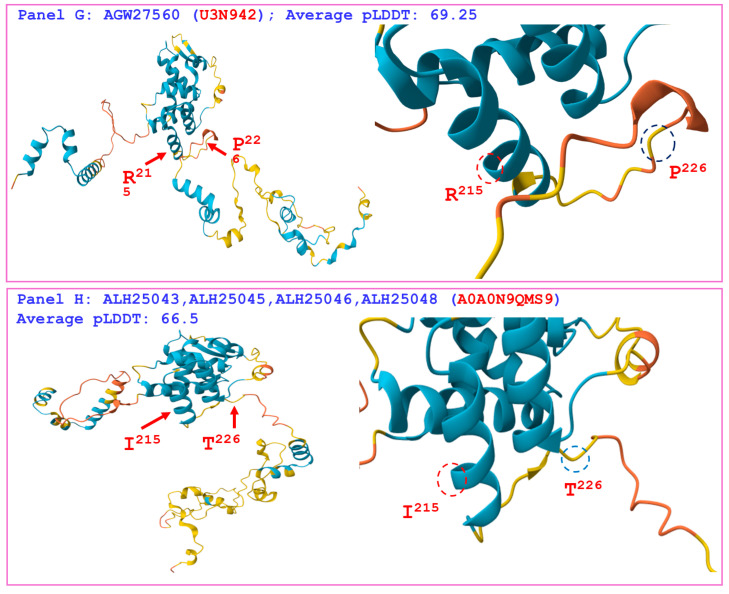
Correlations of changes in the primary, secondary, and tertiary structures of the MATα_HMGbox domains of the reference MAT1-1-1 protein AGW27560 derived from the *H. sinensis* strain CS68-2-1229 [48], and the variant MAT1-1-1 proteins ALH25043, ALH25045, ALH25046, and ALH25048 derived from the wild-type *C. sinensis* isolates YN09_22, YN09_51, YN09_6, and YN09_64, respectively. Panel (**A**) shows an alignment of the amino acid sequences of the MATα_HMGbox domains of the proteins; amino acid substitutions are shown in red, whereas the hyphens indicate identical amino acid residues. The ExPASy ProtScale plots show the changes in hydrophobicity (Panel (**B**)) and in the 2D structures (Panels (**C**–**F**) for α-helices, β-sheets, β-turns, and coils, respectively) of the proteins; the open blue rectangles highlight the topological structure and waveform changes of the plots. Panels (**G**,**H**) show the 3D structures of the full-length proteins on the left and the locally magnified structures at the substitution sites on the right. The model confidence values for the AlphaFold-predicted 3D structures are as follows: ☐ very high (pLDDT > 90); ☐ high (90 > pLDDT > 70); ☐ low (70 > pLDDT > 50); and ☐ very low (pLDDT < 50).

Figure 5 compares the hydrophobicity and the structures of the MATα_HMGbox domains of the MAT1-1-1 proteins ALH25043, ALH25045, ALH25046, and ALH25048 (AlphaFold code A0A0N9QMS9) derived from the wild-type *C. sinensis* isolates YN09_22, YN09_51, YN09_6, and YN09_64, respectively (Table S2) [52], with those of the MATα_HMGbox domain of the reference protein AGW27560 (AlphaFold code U3N942) derived from the *H. sinensis* strain CS68-2-1229 (Table S2) [48]. Panel (A) of Figure 5 shows substitutions of R-to-I (basic arginine to aliphatic isoleucine; hydropathy indices changed from −4.5 to 4.5 [67]) and P-to-T (proline to threonine, both of which possess polar neutral side chains; hydropathy indices changed from −1.6 to −0.7). These substitutions result in increased hydrophobicity, as illustrated by the topological structure and waveform changes that are apparent in the hydropathy plot in Panel (B). They also alter the secondary structure surrounding the mutation sites in the MATα_HMGbox domains of the MAT1-1-1 proteins ALH25043, ALH25045, ALH25046, and ALH25048, as illustrated by the topological structure and waveform changes shown in the ExPASy ProtScale plots for α-helices, β-sheets, β-turns, and coils in Panels (C–F). As shown in the diagrams of the 3D structure in Panels (G and H), the sites of the R-to-I and P-to-T substitutions are inside and outside, respectively, the hydrophobic core formed by the 3 α-helices, and the substantial increase in hydrophobicity alters the tertiary structures of the MATα_HMGbox domains of the MAT1-1-1 proteins under AlphaFold code A0A0N9QMS9. The variant MATα_HMGbox domain clustered into Cluster 2 in the Bayesian clustering tree (Figure 1, Table S6).

**Figure 6:**
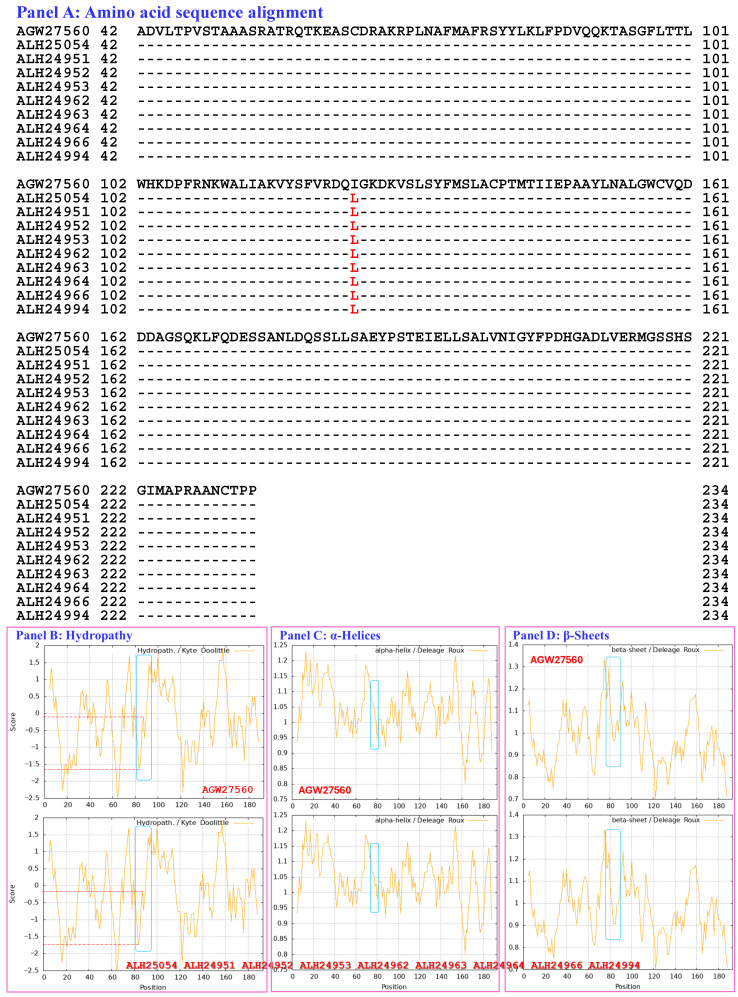

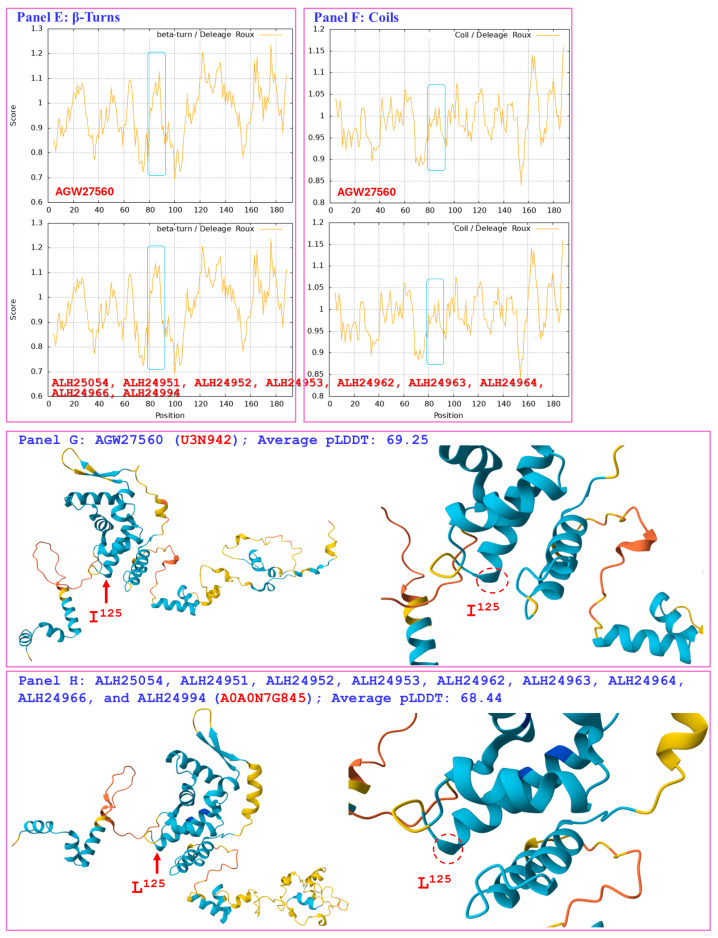
Correlations of changes in the primary, secondary, and tertiary structures of the MATα_HMGbox domains in the reference MAT1-1-1 protein AGW27560 derived from the *H. sinensis* strain CS68-2-1229 [48], and the mutant MAT1-1-1 proteins ALH25054, ALH24951, ALH24952, ALH24953, ALH24962, ALH24963, ALH24964, ALH24966, and ALH24994 derived from the wild-type *C. sinensis* isolates GS09_311, GS09_229, GS09_281, GS10_1, QH09_164, QH09_173, QH09_201, QH09_210, and SC09_87, respectively. Panel (**A**) shows the alignment of the amino acid sequences of the MATα_HMGbox domains of the proteins; amino acid substitutions are shown in red, whereas the hyphens indicate identical amino acid residues. The ExPASy ProtScale plots show the changes in hydrophobicity (Panel (**B**)) and in the 2D structures (Panels (**C**–**F**) for α-helices, β-sheets, β-turns, and coils, respectively) of the proteins; the open blue rectangles highlight the topological structure and waveform changes shown in the plots. Panels (**G**,**H**) show the 3D structures of the full-length proteins on the left and the locally magnified structures at the substitution sites on the right. The model confidence values for the AlphaFold-predicted 3D structures are as follows: ☐ very high (pLDDT > 90); ☐ high (90 > pLDDT > 70); ☐ low (70 > pLDDT > 50); and ☐ very low (pLDDT < 50).

Figure 6 compares the hydrophobicity and structures of the MATα_HMGbox domains of the MAT1-1-1 proteins ALH25054, ALH24951, ALH24952, ALH24953, ALH24962, ALH24963, ALH24964, ALH24966, and ALH24994 (AlphaFold code A0A0N7G845) derived from the wild-type *C. sinensis* isolates GS09_311, GS09_229, GS09_281, GS10_1, QH09_164, QH09_173, QH09_201, QH09_210, and SC09_87, respectively (Table S2) [52], with those of the reference protein AGW27560 (AlphaFold code U3N942) derived from the *H. sinensis* strain CS68-2-1229 (Table S2) [48]. Panel (A) of Figure 6 shows I-to-L substitutions (aliphatic isoleucine to aliphatic leucine, hydropathy indices changed from 4.5 to 3.8 [67]); these substitutions resulted in reduced hydrophobicity, as illustrated by the topological structure and waveform changes shown in the hydropathy plot in Panel (B). As shown in Panels (C–F), the secondary structure of the proteins near the mutation sites in the MATα_HMGbox domains of the MAT1-1-1 proteins ALH25054, ALH24951, ALH24952, ALH24953, ALH24962, ALH24963, ALH24964, ALH24966, and ALH24994 (Table S2) was altered, as illustrated by the topological structure and waveform changes in the ExPASy ProtScale plots for α-helices, β-sheets, β-turns, and coils, respectively. As shown in the representations of the 3D structures presented in Panels (G and H), the substituted amino acid residues are located within the core structures of the 3 α-helices, and they destabilize the hydrophobic core of the protein [84,85], altering the tertiary structures of the MATα_HMGbox domains of the MAT1-1-1 proteins under AlphaFold code A0A0N7G845, which cluster into Branch e1 in the Bayesian clustering tree (Figure 1, Table S6).

**Figure 7:**
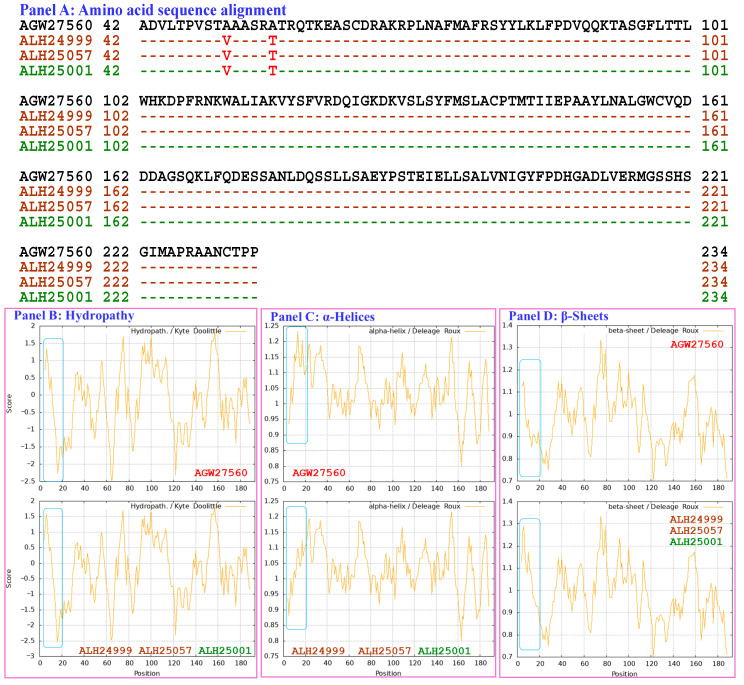

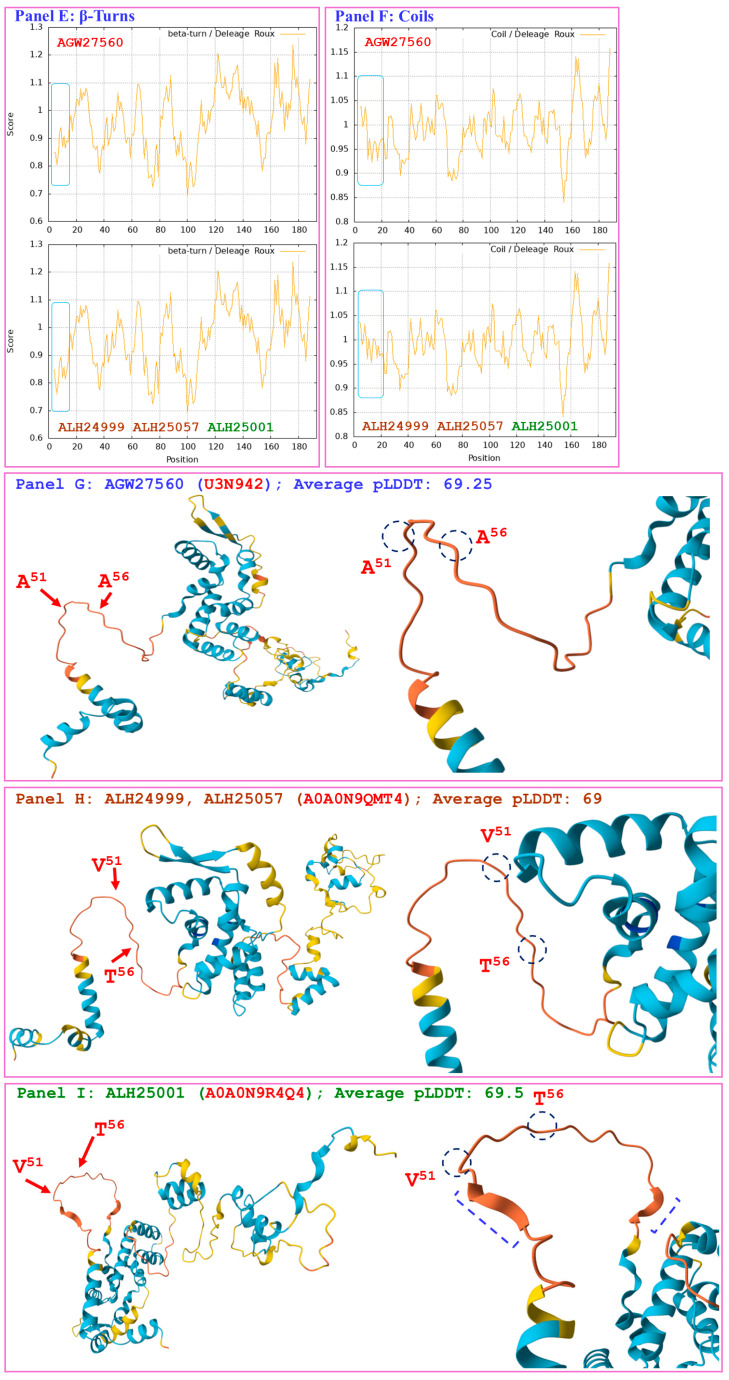
Correlations of the changes in the primary, secondary, and tertiary structures of the MATα_HMGbox domains in the reference MAT1-1-1 protein AGW27560 derived from the *H. sinensis* strain CS68-2-1229 [48], the mutant MAT1-1-1 proteins ALH24999 and ALH25057 (AlphaFold code A0A0N9QMT4), and the protein ALH25001 (AlphaFold code A0A0N9R4Q4) derived from the wild-type *C. sinensis* isolates XZ07_H2, XZ12_16, and XZ05_2, respectively. Panel (**A**) shows an alignment of the amino acid sequences of the MATα_HMGbox domains of the proteins; amino acid substitutions are shown in red; the sequences displayed in brown and green represent the MAT1-1-1 proteins under the AlphaFold codes A0A0N9QMT4 and A0A0N9R4Q4, respectively; and the hyphens indicate identical amino acid residues. The ExPASy ProtScale plots show the changes in hydrophobicity (Panel (**B**)) and in the 2D structures (Panels (**C**–**F**), which display the α-helices, β-sheets, β-turns, and coils, respectively) of the proteins. The open blue rectangles highlight the topological structure and waveform changes shown in the plots. Panels (**G**–**I**) show the 3D structures of the full-length proteins on the left and the locally magnified structures at the substitution sites on the right. The model confidence values for the AlphaFold-predicted 3D structures are as follows: ☐ very high (pLDDT > 90); ☐ high (90 > pLDDT > 70); ☐ low (70 > pLDDT > 50); and ☐ very low (pLDDT < 50).

Figure 7 shows the hydrophobicity and structures of the MATα_HMGbox domains of the MAT1-1-1 proteins ALH24999 and ALH25057 (AlphaFold code A0A0N9QMT4) and the MAT1-1-1 protein ALH25001 (AlphaFold code A0A0N9R4Q4) derived from the wild-type *C. sinensis* isolates XZ07_H2, XZ12_16, and XZ05_2, respectively (Table S2) [52], compared with the reference protein AGW27560 (AlphaFold code U3N942) derived from the *H. sinensis* strain CS68-2-1229 (Table S2) [48]. Panel (A) of Figure 7 shows A-to-V (aliphatic alanine to aliphatic valine; hydropathy indices changed from 1.8 to 4.2 [67]) and A-to-T (aliphatic isoleucine to threonine, an amino acid that contains polar neutral side chains; hydropathy indices changed from 1.8 to −0.7) substitutions. These substitutions altered the hydrophobicity of the protein, as illustrated by the topological structure and waveform changes shown in the hydropathy plot in Panel (B). These changes altered the secondary structure surrounding the mutation sites in the MATα_HMGbox domain of the MAT1-1-1 proteins ALH24999, ALH25057, and ALH25001, as illustrated by the topological structure and waveform changes in the ExPASy ProtScale plots for α-helices, β-sheets, β-turns, and coils shown in Panels (C–F), respectively. As shown in the 3D structures presented in Panels (G–I), the mutation sites are located upstream of the hydrophobic core structure of 3 α-helices in the MATα_HMGbox domains, and mutations at these sites alter the tertiary structures of the MATα_HMGbox domains of MAT1-1-1 proteins under AlphaFold codes A0A0N9QMT4 and A0A0N9R4Q4, which are clustered into Cluster d in the Bayesian clustering tree (Figure 1, Table S6).

Although Panel (A) of Figure 7 shows the same substitutions of A-to-V and A-to-T in the MATα_HMGbox domains of the variant proteins ALH24999, ALH25057, and ALH25001 [52], Panels (H and I) show different tertiary structures for the MATα_HMGbox domains of the MAT1-1-1 protein variants under the AlphaFold codes A0A0N9QMT4 and A0A0N9R4Q4. Specifically, the protein ALH25001 contains 2 short β-sheet structures upstream and downstream of the mutation sites in the MATα_HMGbox domain shown in Panel (I); these structures are marked with dashed half brackets in blue. In contrast, the distinct tertiary structures of the mutant proteins ALH24999 and ALH25057 without similar β-sheet structures are shown in Panel (H).

**Figure 8:**
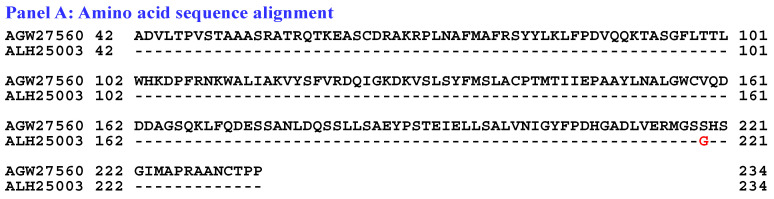

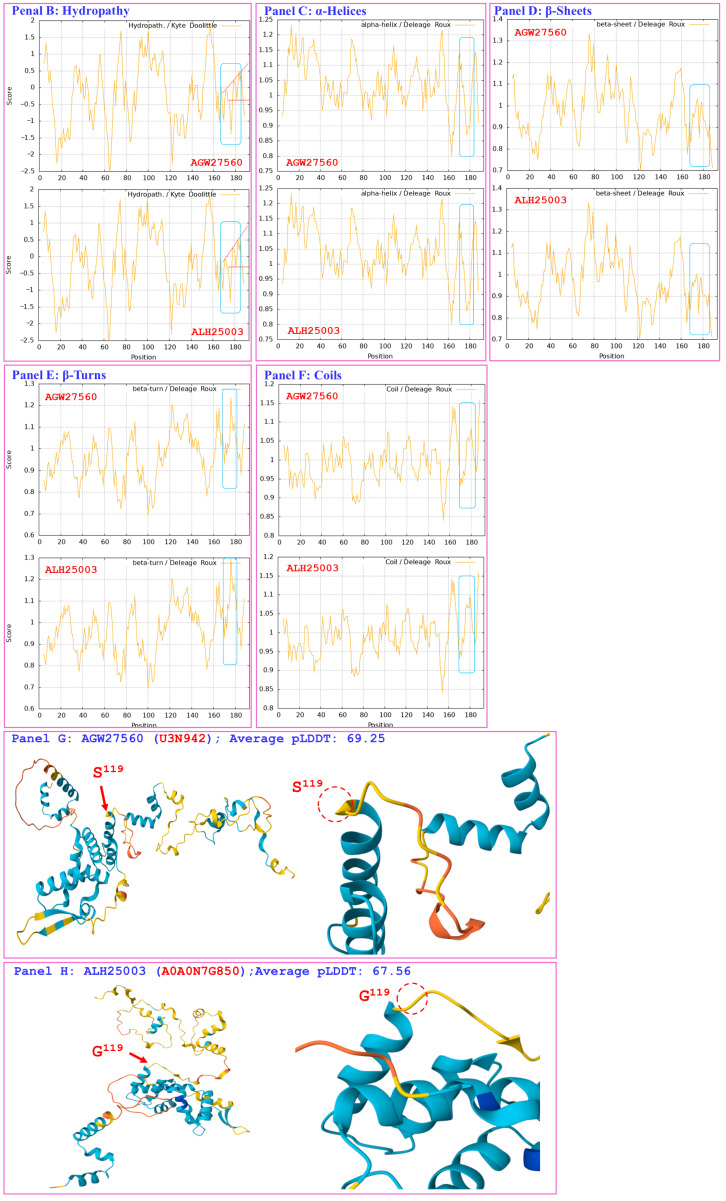
Correlations of the changes in the primary, secondary, and tertiary structures of the MATα_HMGbox domains of the reference MAT1-1-1 protein AGW27560 derived from the *H. sinensis* strain CS68-2-1229 [48] and the mutant MAT1-1-1 protein ALH25003 derived from the wild-type *C. sinensis* isolate XZ05_6. Panel (**A**) shows an alignment of the amino acid sequences of the MATα_HMGbox domains of the MAT1-1-1 proteins; the amino acid substitution is shown in red, whereas the hyphens indicate identical amino acid residues. The ExPASy ProtScale plots show the changes in the hydrophobicity (Panel (**B**)) and in the 2D structures (Panels (**C**–**F**) for the α-helices, β-sheets, β-turns, and coils, respectively) of the proteins, and the open blue rectangles highlight the topological structure and waveform changes shown in the plots. Panels (**G**,**H**) show the 3D structures of the full-length proteins on the left and the locally magnified structures at the substitution sites on the right. The model confidence values for the AlphaFold-predicted 3D structures are as follows: ☐ very high (pLDDT > 90); ☐ high (90 > pLDDT > 70); ☐ low (70 > pLDDT > 50); and ☐ very low (pLDDT < 50).

Figure 8 compares the hydrophobicity and structures of the MATα_HMGbox domain of the MAT1-1-1 protein ALH25003 (AlphaFold code A0A0N7G850) derived from the wild-type *C. sinensis* isolate XZ05_6 (Table S2) [52] with those of the reference protein AGW27560 (AlphaFold code U3N942) derived from the *H. sinensis* strain CS68-2-1229 (Table S2) [48]. Panel (A) of Figure 8 shows an S-to-G substitution (serine, which has polar neutral side chains, is replaced by the unique amino acid glycine; hydropathy indices changed from −0.8 to −0.4 [67]). This substitution resulted in a slight increase in hydrophobicity, as illustrated by the topological structure and waveform changes shown in the hydropathy plot in Panel (B). These changes altered the secondary structure of the mutant MAT1-1-1 protein ALH25003 in the region surrounding the mutation site in the MATα_HMGbox domain, as illustrated by the topological structure and waveform changes in the ExPASy ProtScale plots for α-helices, β-sheets, β-turns, and coils shown in Panels (C–F). As shown in the representations of the 3D structures of the proteins in Panels (G and H), the amino acid residue (serine), which is located at the end of one of the 3 α-helices that form the hydrophobic core structure of the MATα_HMGbox domain [84,85], was replaced by glycine, which became the first residue after the α-helix in the domain. This single-residue substitution plus additional E-to-K and Y-to-H substitutions downstream of the MATα_HMGbox domain (Figure S1) significantly altered the tertiary structure of the protein ALH25003 under the unique AlphaFold code A0A0N7G850. The mutant MATα_HMGbox domain clustered into Branch a2 in the Bayesian clustering tree (Figure 1, Table S6).

Aggregating the data presented in Section 3.5, Panel (A) in Figure 3, Figure 4, Figure 5, Figure 6, Figure 7 and Figure 8 depicts the 1–2 amino acid substitutions in the MATα_HMGbox domains of MAT1-1-1 proteins under various AlphaFold 3D structure codes derived from wild-type *C. sinensis* isolates compared with the reference MAT1-1-1 protein AGW27560 under AlphaFold code U3N942 [48]. The substitutions within the MATα_HMGbox domains caused topological structure and waveform changes shown in the ExPASy ProtScale plots in Panels (B–F) of Figure 3, Figure 4, Figure 5, Figure 6, Figure 7 and Figure 8. These changes indicate differences in the hydrophobicity and secondary structures (α-helices, β-sheets, β-turns, and coils) of the variant full-length proteins. The changes in hydrophobicity and in the primary and secondary structures of the MATα_HMGbox domains altered the AlphaFold 3D structures of the domains of the proteins under various AlphaFold codes (Panels (H) in Figure 3, Figure 4, Figure 5, Figure 6, Figure 7 and Figure 8 and Panel (I) of Figure 7). The mutant domain sequences containing 1 or 2 amino acid substitutions were clustered into different clades in the Bayesian clustering tree (Figure 1, Table S6), and the altered 3D structures of the proteins may ultimately affect the DNA binding affinities and the regulation of the transcription of genes related to the sexual reproduction of *O. sinensis*.

### 3.6. Diverse Primary and Secondary Structures of the MATα_HMGbox Domains of MAT1-1-1 Proteins Encoded by the Genome and Metatranscriptome Assemblies of H. sinensis and C. sinensis Insect–Fungal Complexes

**Figure 9:**
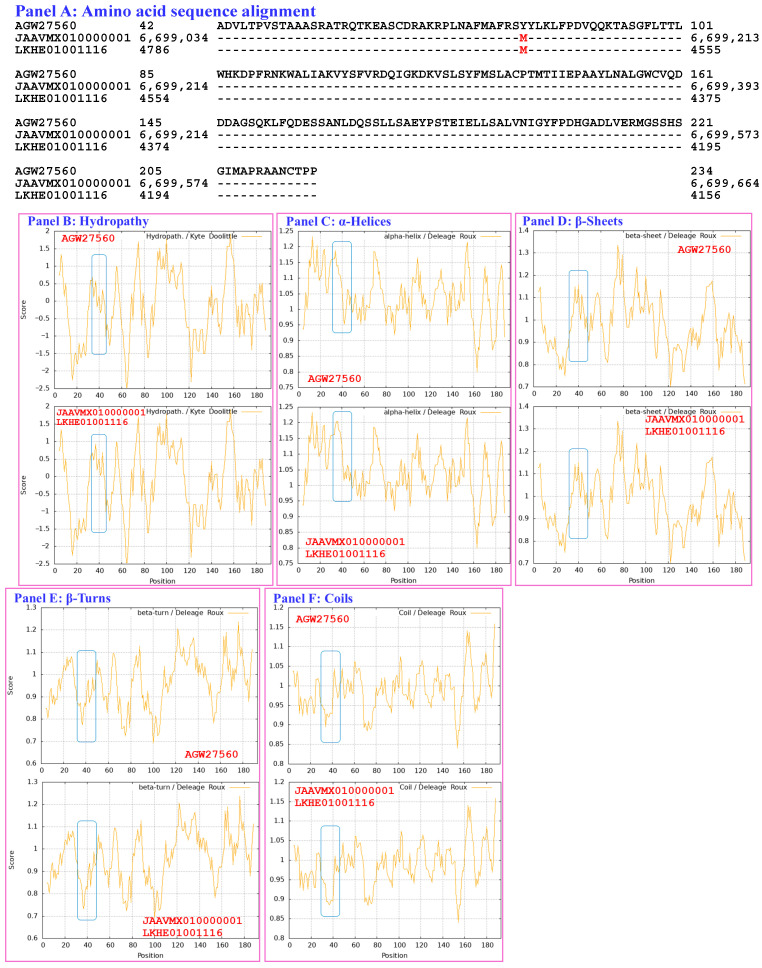
Correlations of the changes in the primary and secondary structures of the MATα_HMGbox domains of the reference MAT1-1-1 protein AGW27560 derived from the *H. sinensis* strain CS68-2-1229 [48] and the truncated MAT1-1-1 proteins encoded by the genome assemblies JAAVMX010000001 and LKHE01001116 of the *H. sinensis* strains IOZ07 and 1229, respectively. Panel (**A**) shows an alignment of the amino acid sequences in the MATα_HMGbox domains of the MAT1-1-1 proteins; amino acid substitutions are shown in red, whereas the hyphens indicate identical amino acid residues. The ExPASy ProtScale plots show the changes in hydrophobicity (Panel (**B**)) and in the 2D structures (Panels (**C**–**F**) show the α-helices, β-sheets, β-turns, and coils, respectively) of the proteins; the open blue rectangles highlight the topological structure and waveform changes shown in the plots.

Figure 9 compares the hydrophobicity and secondary structures (α-helices, β-sheets, β-turns, and coils) of the MATα_HMGbox domains of the MAT1-1-1 proteins encoded by the genome assemblies JAAVMX010000001 (6,699,061→6,699,153 & 6,699,206→6,699,637) and LKHE01001116 (4183←4620 & 4667←4759) of the *H. sinensis* strains IOZ07 and 1229 [60,62] with those of the reference MAT1-1-1 protein AGW27560 derived from the *H. sinensis* strain CS68-2-1229 (Table S2) [48]. Panel (A) of Figure 9 shows a Y-to-M substitution (aromatic tyrosine to methionine, an amino acid that has polar neutral side chains; change in the hydropathy index from −1.3 to 1.9 [67]). This substitution increased the hydrophobicity of the MATα_HMGbox domains of the MAT1-1-1 proteins encoded by the genome assemblies JAAVMX010000001 and LKHE01001116 [60,62], as illustrated by the topological structure and waveform changes in the hydropathy plot shown in Panel (B). These changes resulted in altered secondary structures surrounding the mutation sites in the MATα_HMGbox domains of the MAT1-1-1 proteins, as illustrated in the ExPASy ProtScale plots for α-helices, β-sheets, β-turns, and coils in Panels (C–F), respectively. The MATα_HMGbox domains of the MAT1-1-1 proteins were clustered into Branch c in the Bayesian clustering tree (Figure 1, Table S7).

Compared with the reference MAT1-1-1-1 protein AGW27560 (Figure S1, Tables S2 and S7), no changes were detected in the amino acid sequences, hydrophobicity, or secondary structures (α-helices, β-sheets, β-turns, and coils) of the MATα_HMGbox domains of the MAT1-1-1 proteins encoded by the genome assembly ANOV01017390 (794←1228 & 1280←1369) of the *H. sinensis* strain Co18 or the metatranscriptome assembly OSIN7648 (151→675) of the *C. sinensis* insect–fungal complex collected from Deqin, Yunnan, China [48,49,58,67]. The MATα_HMGbox domains of the genome- and metatranscriptome-encoded MAT1-1-1 proteins clustered into Branch a1 in the Bayesian clustering tree (Figure 1, Table S7).

The MATα_HMGbox domain of the MAT1-1-1 protein encoded by the metatranscriptome assembly GAGW01008880 (714←1127) of the *C. sinensis* insect–fungal complex is truncated at its N-terminus, as shown in Figure S1 and Panel (A) of Figure S3 [63]. The remaining portion of the MATα_HMGbox domain of this protein is 100% identical to the query protein AGW27560 (AlphaFold code U3N942) derived from the *H. sinensis* strain CS68-2-1229 (Table S2) [48], and it showed no apparent changes in hydrophobicity or secondary structure, as illustrated by the identical topology and waveforms in the ExPASy ProtScale plots for hydropathy, α-helices, β-sheets, β-turns, and coils shown in Panels (B–F), respectively, of Figure S3. Although the MATα_HMGbox domain of the metatranscriptome-encoded protein clustered into Branch a1 in the Bayesian clustering tree together with the 6 reference authentic MAT1-1-1 proteins (Figure 1, Table S7), the truncated MATα_HMGbox domain, which lacks 50 and 48 amino acid residues encoded by exons I and II of the *MAT1-1-1* gene, respectively, may not be able to form a core functional stereostructure containing 3 critical α-helices that support high-affinity DNA binding and full functionality in the regulation of the transcription of genes related to the sexual reproduction of *O. sinensis* [50,84,85].

The genome assemblies LWBQ00000000 and NGJJ00000000 and the transcriptome assembly GCQL00000000 of the *H. sinensis* strains ZJB12195, CC1406-20395, and L0106, respectively, do not contain genes or transcripts that encode MAT1-1-1 proteins [58,59,61].

### 3.7. Heteromorphic Stereostructures of the HMG-Box_ROX1-like Domains of the MAT1-2-1 Proteins Derived from Wild-Type C. sinensis Isolates

Section 3.7 presents analyses of the correlations between the changes in the hydrophobicity and structures of the HMG-box_ROX1-like domains of full-length MAT1-2-1 proteins (under 12 AlphaFold codes; Table S8) derived from wild-type *C. sinensis* isolates compared with the reference MAT1-2-1 proteins represented by AEH27625 (AlphaFold code D7F2E9) derived from the *H. sinensis* strain CS2 (Table S3) [56]. The HMG-box_ROX1-like domain sequences (amino acid residues 127→197) were extended both upstream and downstream by 9 external amino acid residues because the sequential ExPASy ProtScale plots for hydropathy, α-helices, β-sheets, β-turns, and coils were constructed at a window size of 9 amino acids (Section 2.5 of the Materials and Methods).

**Figure 10:**
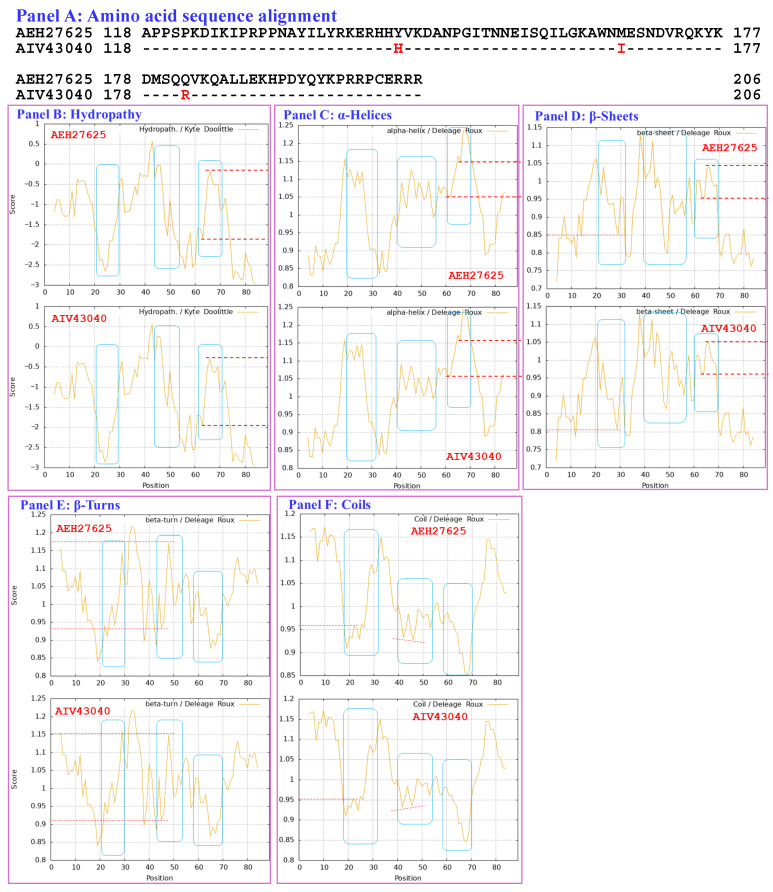

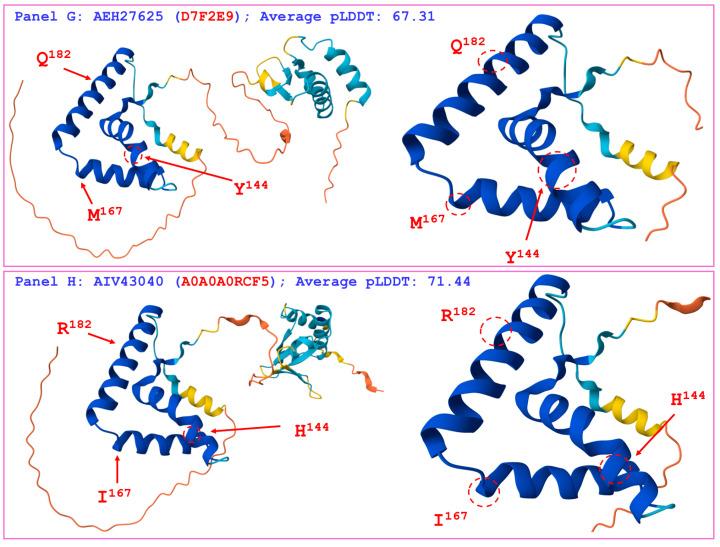
Correlations of the changes in the primary, secondary, and tertiary structures of the HMG-box_ROX1-like domains of the reference MAT1-2-1 protein AEH27625 derived from the *H. sinensis* strain CS2 [56] and the variant MAT1-2-1 protein AIV43040 derived from the wild-type *C. sinensis* isolate XZ12_16. Panel (**A**) shows an alignment of the amino acid sequences of the HMG-box_ROX1-like domains of the proteins; amino acid substitutions are shown in red, whereas the hyphens indicate identical amino acid residues. The ExPASy ProtScale plots show the changes in hydrophobicity (Panel (**B**)) and in the 2D structures (Panels (**C**–**F**) show the α-helices, β-sheets, β-turns, and coils, respectively) of the proteins; the open blue rectangles highlight the topological structure and waveform changes observed in the plots. Panels (**G**,**H**) show representations of the 3D structures of the full-length proteins on the left; the locally magnified structures at the substitution sites are shown on the right. The model confidence values for the AlphaFold-predicted 3D structures are as follows: ☐ very high (pLDDT > 90); ☐ high (90 > pLDDT > 70); ☐ low (70 > pLDDT > 50); and ☐ very low (pLDDT < 50).

Figure 10 compares the hydrophobicity and structures of the HMG-box_ROX1-like domain of the MAT1-2-1 protein AIV43040 (AlphaFold code A0A0A0RCF5) derived from the *C. sinensis* isolate XZ12_16 (Table S3) [5] with those of the reference protein AEH27625 (AlphaFold code D7F2E9) derived from the *H. sinensis* strain CS2 [56]. Panel (A) of Figure 10 shows 3 amino acid substitutions: Y-to-H (aromatic tyrosine to basic histidine; change in the hydropathy index from −1.3 to −3.2 [67]), M-to-I (methionine, which possesses polar neutral side chains, to aliphatic isoleucine; change in the hydropathy index from 1.9 to 4.5) and Q-to-R (glutamine, which has polar neutral side chains, to basic arginine; change in the hydropathy index from −3.5 to −4.5). Individually, these substitutions caused decreased or increased hydrophobicity, as illustrated by the changes in topological structure and waveform changes in the hydropathy plot shown in Panel (B). These changes resulted in altered secondary structures surrounding the mutation sites in the HMG-box_ROX1-like domain of the MAT1-2-1 protein AIV43040, as illustrated by the topological structure and waveform changes in the ExPASy ProtScale plots for α-helices, β-sheets, β-turns, and coils shown in Panels (C–F), respectively. As shown in the representations of the 3D structures in Panels (G and H), the replaced residues are located in each of the 3 core α-helices, and they alter the stability of the hydrophobic core of the HMG-box_ROX1-like domain [84,85] as well as the stereostructure of the DNA-binding domain of the MAT1-2-1 protein AIV43040 under AlphaFold code A0A0A0RCF5. The variant HMG-box_ROX1-like domain clustered into Branch b2β, which displayed the longest clustering distance in the Bayesian clustering tree (Figure 2, Table S8).

**Figure 11:**
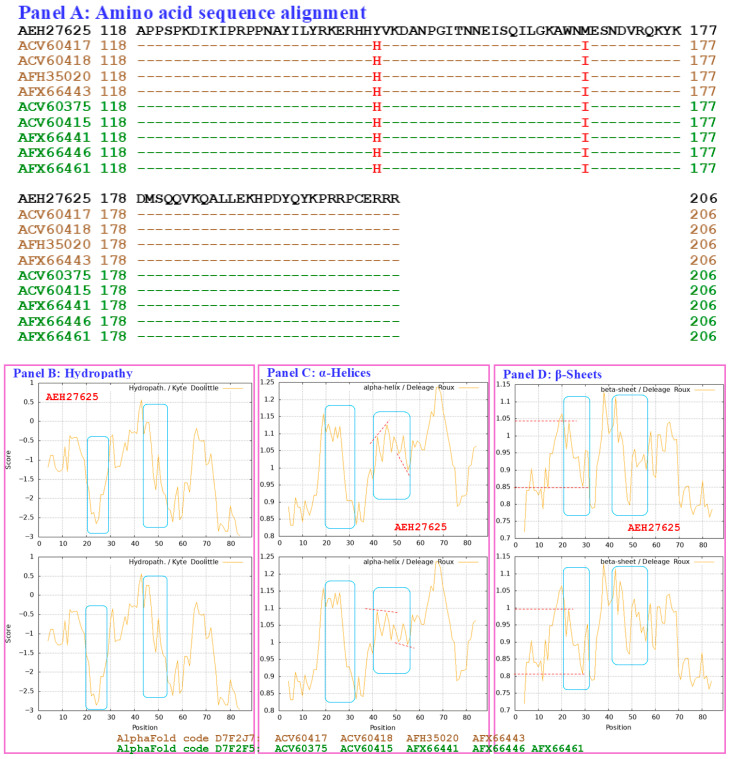

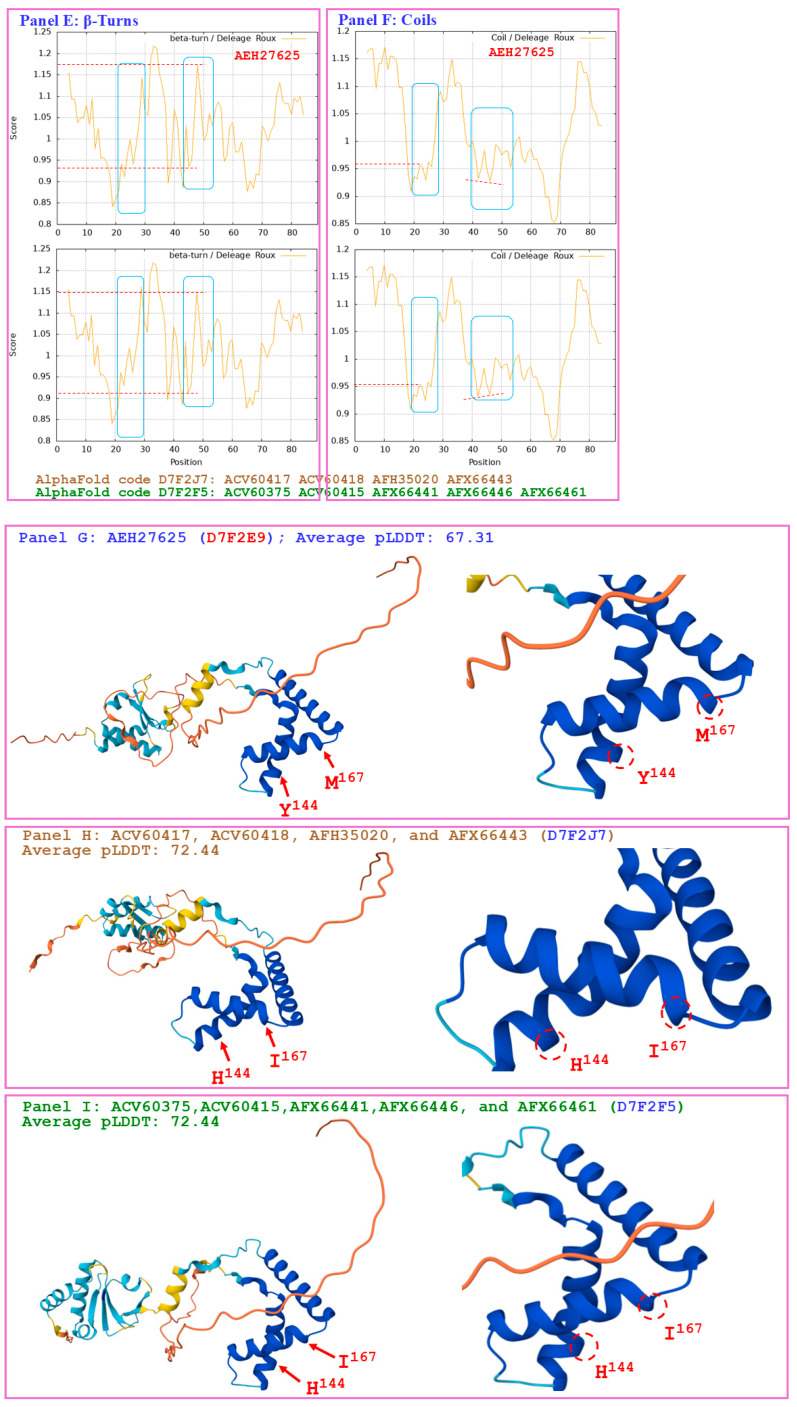
Correlations of the changes in the primary, secondary, and tertiary structures of the HMG-box_ROX1-like domains of the reference MAT1-2-1 protein AEH27625 (AlphaFold code D7F2E9) derived from the *H. sinensis* strain CS2 [56]; the mutant MAT1-2-1 proteins under AlphaFold code D7F2J7 (ACV60417, ACV60418, AFH35020, and AFX66443 shown in brown) derived from the wild-type *C. sinensis* isolate XZ-LZ07-H1, XZ-LZ07-H2, XZ06-124, and XZ05_8); and the mutant proteins under AlphaFold code D7F2F5 (ACV60375, ACV60415, AFX66441, AFX66446, and AFX66461 shown in green) derived from the wild-type *C. sinensis* isolates XZ-SN-44, XZ-LZ05-6, XZ05_2, XZ06_260, and XZ09_80. Panel (**A**) shows an alignment of the amino acid sequences of the HMG-box_ROX1-like domains of the MAT1-2-1 proteins; amino acid substitutions are shown in red; the sequences displayed in brown and green represent the MAT1-2-1 proteins under AlphaFold codes D7F2J7 and D7F2F5, respectively; and the hyphens indicate identical amino acid residues. The ExPASy ProtScale plots show the changes in hydrophobicity (Panel (**B**)) and in the 2D structures (Panels (**C**–**F**) for α-helices, β-sheets, β-turns, and coils, respectively) of the HMG-box_ROX1-like domains of the proteins; the open blue rectangles highlight the topological structure and waveform changes seen in the plots. Panels (**G**–**I**) show representations of the 3D structures of the full-length proteins on the left and of the locally magnified structures at the substitution sites on the right. The model confidence values for the AlphaFold-predicted 3D structures are as follows: ☐ very high (pLDDT > 90); ☐ high (90 > pLDDT > 70); ☐ low (70 > pLDDT > 50); and ☐ very low (pLDDT < 50).

Figure 11 compares the hydrophobicity and structures of the HMG-box_ROX1-like domains of the MAT1-2-1 proteins under AlphaFold code D7F2J7 (ACV60417, ACV60418, AFH35020, and AFX66443 shown in brown in Figure 11, derived from the wild-type *C. sinensis* isolates XZ-LZ07-H1, XZ-LZ07-H2, XZ06-124, and XZ05_8, respectively) and the hydrophobicity and structures of the proteins under AlphaFold code D7F2F5 (ACV60375, ACV60415, AFX66441, AFX66446, and AFX66461 shown in green in Figure 11, derived from the wild-type *C. sinensis* isolates XZ-SN-44, XZ-LZ05-6, XZ05_2, XZ06_260, and XZ09_80, respectively) (Table S3) [5,34,56] with those of the reference protein AEH27625 (AlphaFold code D7F2E9) derived from the *H. sinensis* strain CS2 (Table S3) [56]. Panel (A) of Figure 11 shows substitutions of Y-to-H (aromatic tyrosine to basic histidine; change in the hydropathy index from −1.3 to −3.2 [67]) and M-to-I (methionine, which contains polar neutral side chains, to aliphatic isoleucine; change in the hydropathy index from 1.9 to 4.5). These substitutions changed the hydrophobicity, as illustrated by the topological structure and waveform changes shown in the hydropathy plot in Panel (B). They also altered the secondary structures surrounding the mutation sites in the HMG-box_ROX1-like domains of the mutant MAT1-2-1 proteins ACV60417, ACV60418, AFH35020, and AFX66443 (AlphaFold code D7F2J7) and in those regions in the proteins ACV60375, ACV60415, AFX66441, AFX66446, and AFX66461 (AlphaFold code D7F2F5), as illustrated by the topological structure and waveform changes shown in Panels (C–F), which display the ExPASy ProtScale plots for α-helices, β-sheets, β-turns, and coils. As shown in the 3D structures presented in Panels (G–I) of Figure 11, the substituted residues are located within two of the 3 core α-helices, and they differentially alter the stability of the core structure of the HMG-box_ROX1-like domain [84,85] and the tertiary structure of this domain in the MAT1-2-1 proteins under AlphaFold 3D structural codes D7F2J7 and D7F2F5. The sequences of the HMG-box_ROX1-like domain of the mutant proteins ACV60417, ACV60418, AFH35020, AFX66443, ACV60375, ACV60415, AFX66441, AFX66446, and AFX66461 clustered into Branch b2α in the Bayesian clustering tree (Figure 2, Table S8).

**Figure 12:**
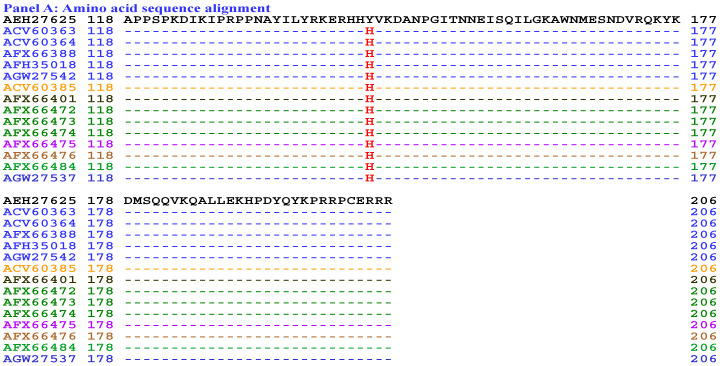

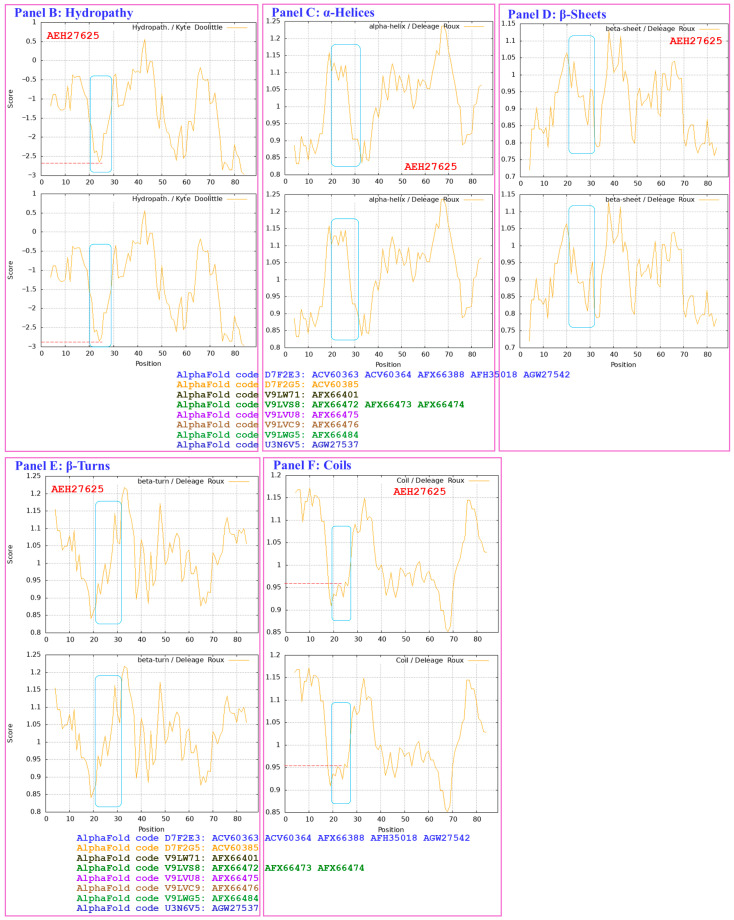

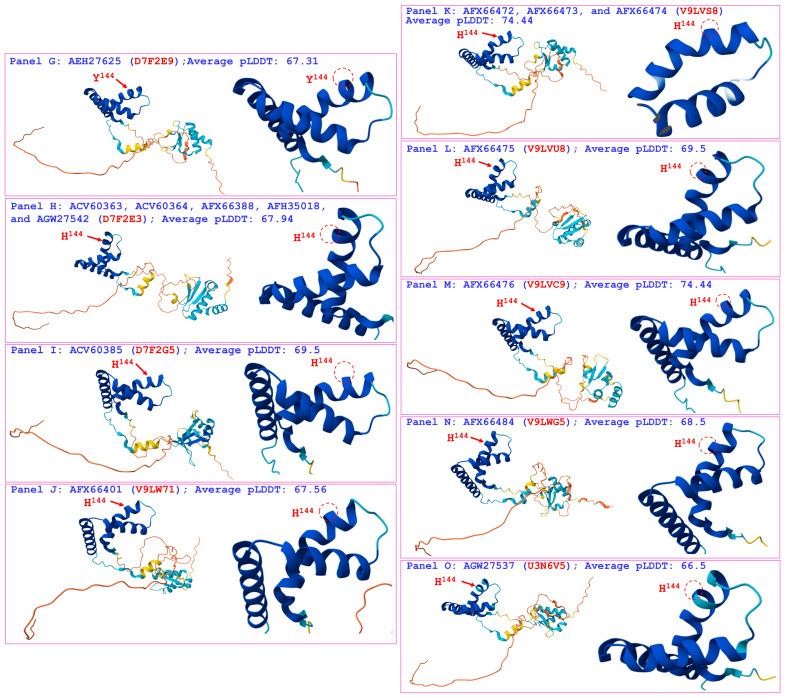
Correlations of the changes in the hydrophobicity and the primary, secondary, and tertiary structures of the HMG-box_ROX1-like domains of the reference MAT1-2-1 protein AEH27625 (AlphaFold code **D7F2E9**) derived from the *H. sinensis* strain CS2 [56], and the mutant MAT1-2-1 proteins under AlphaFold codes **D7F2E3** (ACV60363, ACV60364, AFX66388, AFH35018, and AGW27542), **D7F2G5** (ACV60385), **V9LW71** (AFX66401), **V9LVS8** (AFX66472, AFX66473, and AFX66474), **V9LVU8** (AFX66475), **V9LWC9** (AFX66476), **V9LWG5** (AFX66484), and **U3N6V5** (AGW27537) derived from the wild-type *C. sinensis* isolates YN09_64, YN09_6, YN09_22, YN09_51, XZ-NQ-154, XZ-NQ-155, GS09_111, QH09-93, CS560-961, QH-YS-199, QH09_11, ID10_1, and CS6-251, respectively. Panel (**A**) shows an alignment of the amino acid sequences of the HMG-box_ROX1-like domains of the proteins; amino acid substitutions are shown in red, and the sequences depicted in various colors represent the MAT1-2-1 proteins under AlphaFold codes V9LWC9, V9LVS8, D7F2E3, V9LVU8, D7F2G5, V9LW71, V9LWG5, and U3N6V5; the hyphens indicate identical amino acid residues. The ExPASy ProtScale plots show the changes in hydrophobicity (Panel (**B**)) and in the 2D structures (Panels (**C**–**F**) for α-helices, β-sheets, β-turns, and coils, respectively) of the proteins. The open blue rectangles highlight the topological structure and waveform changes shown in the plots. Panels (**G**–**O**) show representations of the 3D structures of the full-length proteins on the left and the locally magnified structures at the mutation site on the right. The model confidence values for the AlphaFold-predicted 3D structures are as follows: ☐ very high (pLDDT > 90); ☐ high (90 > pLDDT > 70); ☐ low (70 > pLDDT > 50); and ☐ very low (pLDDT < 50).

Figure 12 compares the hydrophobicity and structures of the HMG-box_ROX1-like domains of the MAT1-2-1 proteins under AlphaFold codes **D7F2E3** (ACV60363, ACV60364, AFX66388, AFH35018, and AGW27542), **D7F2G5** (ACV60385), **V9LW71** (AFX66401), **V9LVS8** (AFX66472, AFX66473, and AFX66474), **V9LVU8** (AFX66475), **V9LWC9** (AFX66476), **V9LWG5** (AFX66484), and **U3N6V5** (AGW27537) derived from the wild-type *C. sinensis* isolates YN09_64, YN09_6, YN09_22, YN09_51, XZ-NQ-154, XZ-NQ-155, GS09_111, QH09-93, CS560-961, QH-YS-199, QH09_11, ID10_1, and CS6-251, respectively (Table S3) [5,34,48,56], with the hydrophobicity and structure of the reference protein AEH27625 (AlphaFold code D7F2E9) derived from the *H. sinensis* strain CS2 (Table S3) [56]. Panel (A) of Figure 12 shows Y-to-H substitutions (aromatic tyrosine to basic histidine; change in the hydropathy index from −1.3 to −3.2 [67]) in the HMG-box_ROX1-like domains of all the mutant MAT1-2-1 proteins mentioned above; these substitutions caused reduced hydrophobicity, as illustrated by the topological structure and waveform changes shown in the hydropathy plots in Panel (B). The substitutions altered the secondary structure surrounding the mutation sites in the HMG-box_ROX1-like domains of the MAT1-2-1 proteins, as illustrated by the topological structure and waveform changes in the ExPASy ProtScale plots for α-helices, β-sheets, β-turns, and coils that are shown in Panels (C–F), respectively.

As shown in the representations of the tertiary structures presented in Panels (G–O) of Figure 12, the substituted residues with reduced hydrophobicity are located within the core α-helices in the HMG-box_ROX1-like domains, and they destabilize the hydrophobic core and alter the hydrophobic stereostructures of the domains [84,85]. Although Panel (A) of Figure 12 shows the same Y-to-H substitution that caused changes in hydrophobicity and secondary structure [shown in the ExPASy ProtScale plots in Panels (B–F)] in the HMG-box_ROX1-like domains of the MAT1-2-1 proteins ACV60363–ACV60364, ACV60385, AFH35018, AFX66388, AFX66472–AFX66476, AFX66401, AFX66484, AGW27537, and AGW27542, additional amino acid substitutions outside the HMG-box_ROX1-like domains (Figure S2) might exert synergistic effects on altering the tertiary structures of the proteins through long-range spatial interactions with the mutations within the domains [Panels (H–O) of Figure 12] of the MAT1-2-1 proteins under the 8 AlphaFold codes summarized in Table S8. The mutant HMG-box_ROX1-like domains clustered into Branch b1α in the Bayesian clustering tree (Figure 2, Table S8).

**Figure 13:**
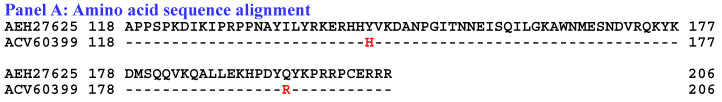

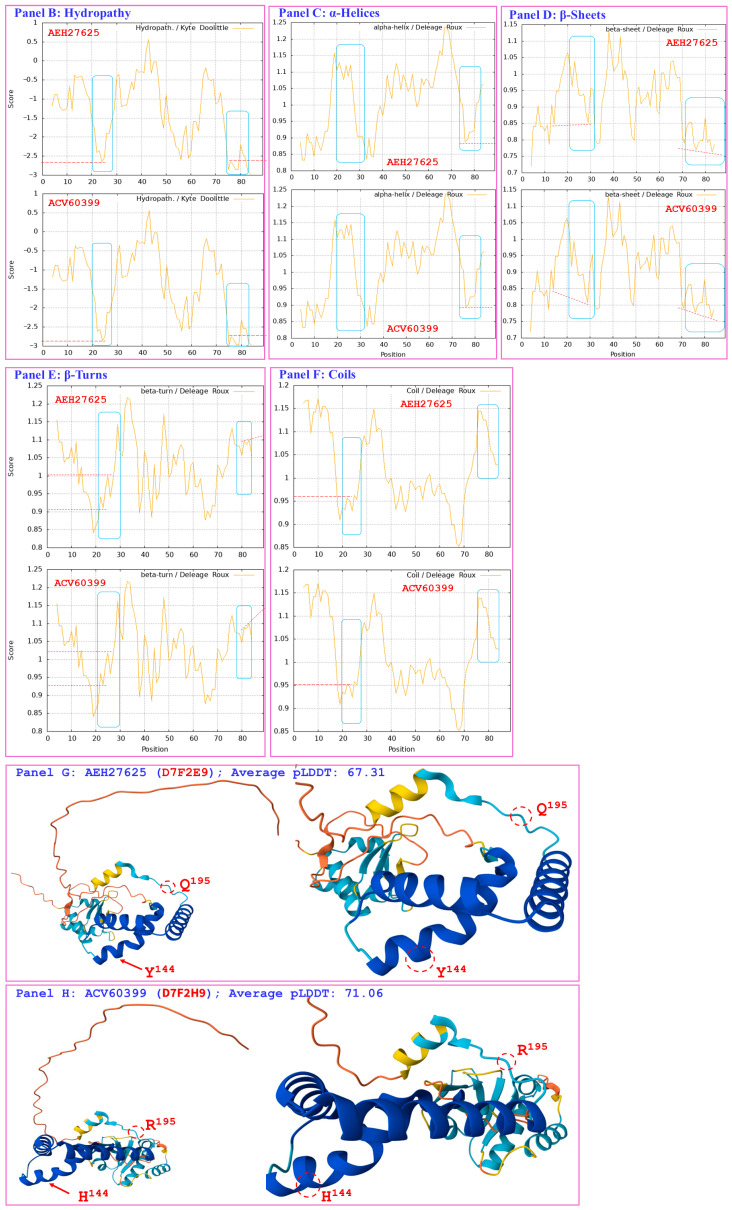
Correlations of the changes in the primary, secondary, and tertiary structures of the HMG-box_ROX1-like domains of the reference MAT1-2-1 protein AEH27625 derived from the *H. sinensis* strain CS2 [56] and the mutant MAT1-2-1 protein ACV60399 derived from the wild-type *C. sinensis* isolate SC-3. Panel (**A**) shows an alignment of the amino acid sequences of the HMG-box_ROX1-like domains of the proteins; amino acid substitutions are shown in red, whereas the hyphens indicate identical amino acid residues. The ExPASy ProtScale plots show the changes in hydrophobicity (Panel (**B**)) and in the 2D structures (Panels (**C**–**F**) for α-helices, β-sheets, β-turns, and coils) of the proteins; the open blue rectangles highlight the topological structure and waveform changes observed in the plots. Panels (**G**,**H**) show the 3D structures of the full-length proteins on the left and the locally magnified structures at the substitution sites on the right. The model confidence values for the AlphaFold-predicted 3D structures are as follows: ☐ very high (pLDDT > 90); ☐ high (90 > pLDDT > 70); ☐ low (70 > pLDDT > 50); and ☐ very low (pLDDT < 50).

Figure 13 compares the hydrophobicity and structure of the HMG-box_ROX1-like domain of the MAT1-2-1 protein ACV60399 (AlphaFold code D7F2H9) derived from the wild-type *C. sinensis* isolate SC-3 (Table S3) [56] with those of the HMG-box_ROX1-like domain of the reference protein AEH27625 (AlphaFold code D7F2E9) derived from the *H. sinensis* strain CS2 (Table S3) [56]. Panel (A) of Figure 13 shows substitutions of Y-to-H (aromatic tyrosine to basic histidine; change in the hydropathy index from −1.3 to −3.2 [67]) and Q-to-R (glutamine, which possesses polar neutral side chains, to basic arginine; change in the hydropathy index from −3.5 to −4.5); both of these substitutions reduced the hydrophobicity, as illustrated by the topological structure and waveform changes shown in the hydropathy plot in Panel (B). These changes altered the secondary structure surrounding the mutation sites in the HMG-box_ROX1-like domain of the mutant MAT1-2-1 protein ACV60399, as illustrated by the topological structure and waveform changes in the ExPASy ProtScale plots for α-helices, β-sheets, β-turns, and coils presented in Panels (C–F). As shown in the representations of 3D structures in Panels (G and H) of Figure 13, the 2 replaced residues with reduced hydrophobicity are located inside and outside the hydrophobic core structure of the 3 α-helices, and they destabilize and alter the core stereostructure of the domain [84,85]. The HMG-box_ROX1-like domain of the mutant protein ACV60399 under AlphaFold code D7F2H9 clustered into Branch b1β in the Bayesian clustering tree (Figure 2, Table S8).

Aggregating the data presented in Section 3.7, Panel (A) in Figure 10, Figure 11, Figure 12 and Figure 13 presents the 1–3 amino acid substitutions detected in the HMG-box_ROX1-like domains that altered the topologies and waveforms shown in the ExPASy ProtScale plots presented in Panels (B–F) of the figures. These changes indicate the presence of altered hydrophobicity and secondary structures (α-helices, β-sheets, β-turns, and coils) of the HMG-box_ROX1-like domains of the mutant MAT1-2-1 proteins. These changes altered the stereostructures of the domains of the mutant proteins under different AlphaFold codes, as illustrated in Panel (H) of Figure 10, Figure 11, Figure 12 and Figure 13, Panel (I) of Figure 11, Panels (I–O) of Figure 12 and summarized in Table S8. The mutant HMG-box_ROX1-like domains clustered into various clades in the Bayesian clustering tree (Figure 2, Table S8) and ultimately changed the DNA binding specificities and affinities and the regulation of gene transcription, which are factors that play key roles in the sexual reproduction of *O. sinensis*.

### 3.8. Diverse Primary and Secondary Structures of the HMG-Box_ROX1-like Domains of MAT1-2-1 Proteins Encoded by the Genome and Transcriptome Assemblies of H. sinensis and the Metatranscriptome Assembly of the C. sinensis Insect–Fungal Complex

**Figure 14:**
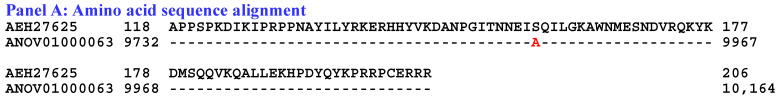

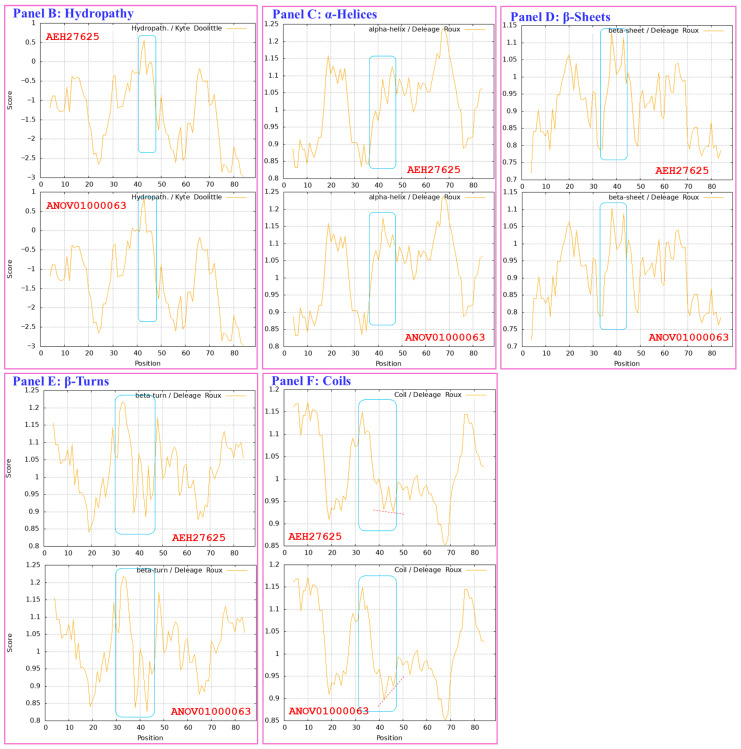
Correlations of the changes in the primary and secondary structures of the HMG-box_ROX1-like domains of the reference MAT1-2-1 protein AEH27625 derived from the *H. sinensis* strain CS2 [56] and the mutant MAT1-2-1 protein encoded by the genome assembly ANOV01000063 derived from the *H. sinensis* strain Co18. Panel (**A**) shows an alignment of the amino acid sequences of the HMG-box_ROX1-like domains of the proteins; amino acid substitutions are shown in red, whereas the hyphens indicate identical amino acid residues. The ExPASy ProtScale plots show the changes in hydrophobicity (Panel (**B**)) and in the 2D structures (Panels (**C**–**F**) for α-helices, β-sheets, β-turns, and coils, respectively) of the proteins; the open blue rectangles highlight the topological structure and waveform changes observed in the plots.

Figure 14 shows a comparison of the hydrophobicity and structure of the HMG-box_ROX1-like domain of the MAT1-2-1 protein encoded by the genome assembly ANOV01000063 (9759→9851 & 9907→10,026) derived from *H. sinensis* strain Co18 [49] with the hydrophobicity and structure of the reference MAT1-2-1 protein AEH27625 derived from *H. sinensis* strain CS2 (AlphaFold code D7F2E9) (Table S3) [56]. Panel (A) of Figure 14 shows an S-to-A substitution (serine with polar neutral side chains to aliphatic alanine; hydropathy indices changed from −0.8 to 1.8 [67]). This substitution increased the hydrophobicity surrounding the mutation site in the domain of the MAT1-2-1 protein encoded by the genome assembly ANOV01000063, as illustrated by the topological structure and waveform changes shown in the hydropathy plot in Panel (B).

These changes in amino acid sequence and hydrophobicity resulted in an altered secondary structure (α-helices, β-sheets, β-turns, and coils) within the HMG-box_ROX1-like domain of the mutant MAT1-2-1 protein encoded by the genome assembly ANOV01000063, as illustrated in the ExPASy ProtScale plots for α-helices, β-sheets, β-turns, and coils shown in Panels (C–F) of Figure 14, respectively [84,85]. These changes in sequence and hydrophobicity may have altered the tertiary structure of the HMG-box_ROX1-like domain of the genome-encoded protein. The altered HMG-box_ROX1-like domain clustered into Branch a2 in the Bayesian clustering tree (Figure 2, Table S9).

**Figure 15:**
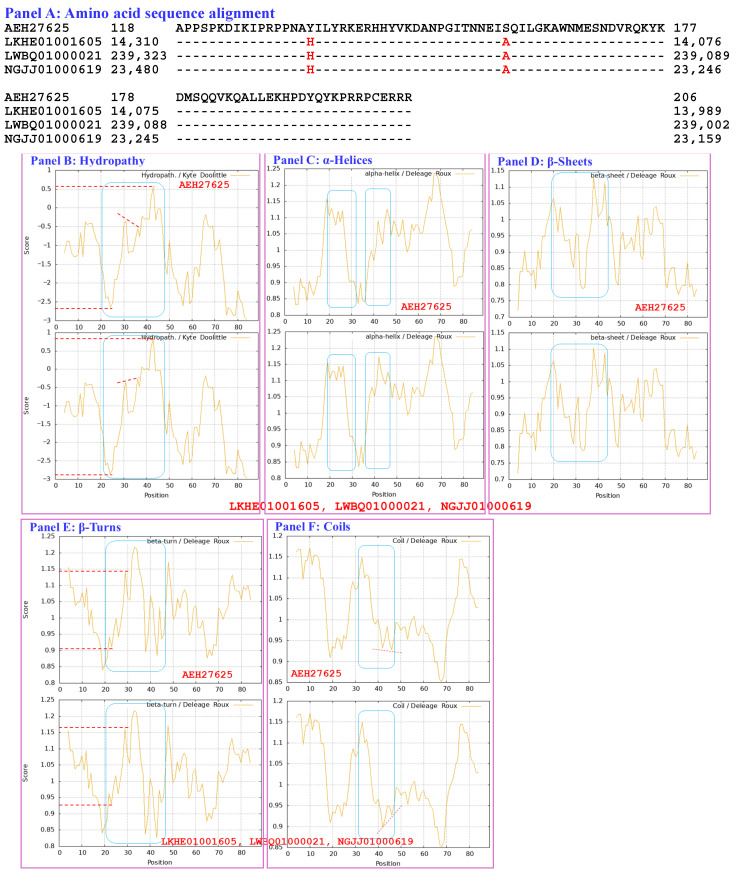
Correlations of the changes in the primary and secondary structures of the HMG-box_ROX1-like domain of the reference MAT1-2-1 protein AEH27625 derived from the *H. sinensis* strain CS2 [56] and the MAT1-2-1 protein variants encoded by the genome assemblies LKHE01001605, LWBQ01000021, and NGJJ01000619 derived from *H. sinensis* strains 1229, ZJB12195, and CC1406-20395, respectively. Panel (**A**) shows an alignment of the amino acid sequences of the HMG-box_ROX1-like domains of the proteins; amino acid substitutions are shown in red, whereas the hyphens indicate identical amino acid residues. The ExPASy ProtScale plots show the changes in hydrophobicity (Panel (**B**)) and in the 2D structures (Panels (**C**–**F**) for α-helices, β-sheets, β-turns, and coils, respectively) of the proteins; the open blue rectangles highlight the topological structure and waveform changes observed in the plots.

Figure 15 compares the hydrophobicity and structures of the HMG-box_ROX1-like domains of the MAT1-2-1 proteins encoded by the genome assemblies LKHE01001605 (14,016←14,135 & 14,191←14,283), LWBQ01000021 (14,016←14,135 & 14,191←14,283), and NGJJ01000619 (23,186←23,305 & 23,361←23,453) of the *H. sinensis* strains 1229, ZJB12195, and CC1406-20395, respectively [59,60,61], with the hydrophobicity and structure of the reference protein AEH27625 (AlphaFold code D7F2E9) derived from the *H. sinensis* strain CS2 (Table S3) [56]. Panel (A) of Figure 15 shows Y-to-H (aromatic tyrosine to basic histidine; change in the hydropathy index from −1.3 to −3.2 [67]) and S-to-A (serine, which has polar neutral side chains, to aliphatic alanine; change in the hydropathy index from −0.8 to 1.8) substitutions, which resulted in altered hydrophobicity, as illustrated in the hydropathy plot in Panel (B).

These changes altered the secondary structures surrounding the mutation sites in the HMG-box_ROX1-like domains of the mutant MAT1-2-1 proteins encoded by the genome assemblies LKHE01001605, LWBQ01000021, and NGJJ01000619, as illustrated in the ExPASy ProtScale plots for α-helices, β-sheets, β-turns, and coils shown in Panels (C–F) of Figure 15, respectively [84,85], and subsequently altered the tertiary structures of the core stereostructures of the domains. The mutant HMG-box_ROX1-like domains of the genome-encoded proteins clustered into Branch b3 in the Bayesian clustering tree (Figure 2, Table S9). The genome assembly JAAVMX000000000 of the *H. sinensis* strain IOZ07 does not contain a gene encoding the MAT1-2-1 protein [62].

**Figure 16:**
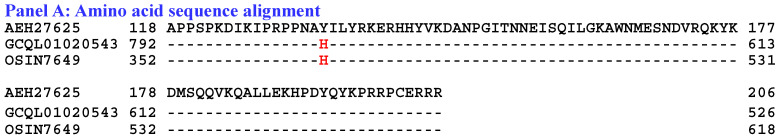

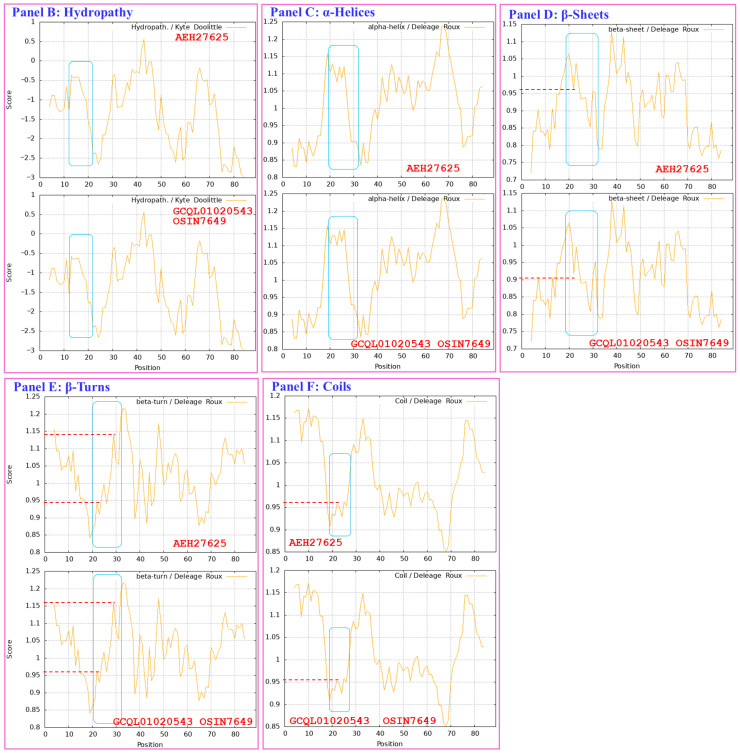
Correlations of the primary and secondary structures of the HMG-box_ROX1-like domain of the reference MAT1-2-1 protein AEH27625 derived from the *H. sinensis* strain CS2 [56] with the primary and secondary structures of the mutant MAT1-2-1 proteins encoded by the transcriptome assembly GCQL01020543 derived from the *H. sinensis* strain L0106 and the metatranscriptome assembly OSIN7649 (379→591) of the mature *C. sinensis* insect–fungal complex. Panel (**A**) shows an alignment of the amino acid sequences of the HMG-box_ROX1-like domains of the proteins; amino acid substitutions are shown in red, whereas the hyphens indicate identical amino acid residues. The ExPASy ProtScale plots show the changes in hydrophobicity (Panel (**B**)) and in the 2D structures (Panels (**C**–**F**) for α-helices, β-sheets, β-turns, and coils, respectively) of the proteins; the open blue rectangles highlight the topological structure and waveform changes observed in the plots.

Figure 16 compares the hydrophobicity and structure of the HMG-box_ROX1-like domain of the MAT1-2-1 proteins encoded by the transcriptome assembly GCQL01020543 (553←765) of the *H. sinensis* strain L0106 and by the metatranscriptome assembly OSIN7649 (379→591) of the mature *C. sinensis* insect–fungal complex [58,64] with the hydrophobicity and structure of the reference protein AEH27625 (AlphaFold code D7F2E9) derived from the *H. sinensis* strain CS2 (Table S3) [56]. Panel (A) of Figure 16 shows Y-to-H substitutions (aromatic tyrosine to basic histidine; change in the hydropathy index from −1.3 to −3.2 [67]). The substitutions decreased the hydrophobicity, as illustrated by the topological structure and waveform changes shown in the hydropathy plot in Panel (B). These changes altered the secondary structure surrounding the mutation sites in the HMG-box_ROX1-like domains of the MAT1-2-1 proteins encoded by the transcriptome assembly GCQL01020543 and the metatranscriptome assembly OSIN7649, as illustrated by the topological structure and waveform changes shown in the ExPASy ProtScale plots for α-helices, β-sheets, β-turns, and coils in Panels (C–F).

Similar to the Y-to-H substitutions in the HMG-box_ROX1-like domains of the mutant MAT1-2-1 proteins under various AlphaFold 3D structural models (such as V9LWC9, V9LVS8, D7F2E3, V9LVU8, D7F2G5, V9LW71, V9LWG5, and U3N6V5) shown in Figure 12, the changes in hydrophobicity and primary and secondary structures shown in Figure 16 may subsequently alter the tertiary structures of the HMG-box_ROX1-like domains of the transcriptome-encoded and metatranscriptome-encoded mutant proteins. The mutant HMG-box_ROX1-like domains of the transcriptome- and metatranscriptome-encoded proteins clustered into Branch b1α in the Bayesian clustering tree (Figure 2, Table S9).

### 3.9. Heterogenous Fungal Sources of the MAT1-1-1 and MAT1-2-1 Proteins

Although wild-type *C. sinensis* isolates are often considered impure *H. sinensis*, an analysis of mutant full-length mating proteins revealed their possible heterogeneous fungal sources, as shown in Table S4. Among the wild-type *C. sinensis* isolates whose full-length MAT1-1-1 and MAT1-2-1 proteins were analyzed and are presented in Figure 3, Figure 4, Figure 5, Figure 6, Figure 7, Figure 8, Figure 10, Figure 11, Figure 12 and Figure 13, 34 had ITS genotyping information in the GenBank database and belonged to GC-biased Genotypes #1 and #3 of genome-independent *O. sinensis* fungi (Table S4) [7]. Although the ITS sequences of 29 of the 34 wild-type *C. sinensis* isolates appeared to show high homology (≥97%) to the ITS sequences of GC-biased Genotype #1 *H. sinensis*, the mutant MAT1-1-1 and MAT1-2-1 proteins might not have been derived from GC-biased Genotype #1 *H. sinensis* within the impure fungal complex or wild-type fungal isolates that contains more than one fungal species [7,86]. Actually, the *H. sinensis* ITS sequence is most easily amplified during traditional single-step PCR under the experimental settings, except when multiple pairs of primers and a touch-down PCR protocol were employed combining with amplicon cloning techniques with selection and sequencing of sufficient (usually 30–50) white colonies. In other words, because of the impure nature of the wild-type *C. sinensis* isolates [7,86], the protein variants may have been derived from cooccurring unvalidated heterospecific or genotypic fungi.

## 4. Discussion

Previous studies [54,55] reported the differential occurrence, differential translation, and alternative splicing of *MAT1-1-1* and *MAT1-2-1* and pheromone receptor genes of *H. sinensis*, wild-type *C. sinensis* isolates, and *C. sinensis* insect-fungal complex. The evidence from the genetic and transcriptional level studies indicates that *O. sinensis* experiences self-sterility and uses a heterothallic or hybrid (even parasexuality) strategy to accomplish sexual reproduction during the lifecycle of the *C. sinensis* insect–fungal complex [10,87,88,89,90,91,92,93,94]. Li et al. [50] reported the 3D structural changes of the complete mating proteins, further validating the self-sterility hypothesis for *O. sinensis* at the protein structure level. They demonstrated that the entire MAT1-1-1 and MAT1-2-1 proteins contribute to 15 and 17 stereostructure heteromorphs, belonging to 5 and 5 Bayesian clusters, respectively. The research presented in this paper continues our previous studies, with a focus on various amino acid substitutions within the MATα_HMGbox and HMG-box_ROX1-like domains of the full-length MAT1-1-1 and MAT1-2-1 proteins, respectively, which are derived from wild-type *C. sinensis* isolates. The MATα_HMGbox domains of MAT1-1-1 proteins with different amino acid substitutions are clustered to 5 Bayesian clusters, with or without branches, as shown in Figure 1 and summarized in Tables S6 and S7. The HMG-box_ROX1-like domains of the MAT1-2-1 proteins are clustered to 2 branched Bayesian clusters, as shown in Figure 2 and summarized in Tables S8 and S9. The amino acid substitutions within the DNA-binding domains significantly affect the hydrophobicity of functional domains and alter the secondary and tertiary structures of mating proteins, especially the tertiary structures of hydrophobic core formed by 3 α-helices within the DNA-binding domains, ultimately affecting the synergistic functionality of the key functional domains of mating proteins in the sexual reproduction of *O. sinensis*.

### 4.1. Historical and Current Usage of Latin Names Regarding Cordyceps sinensis, Hirsutella sinensis, and Ophiocordyceps sinensis to Refer to Fungus/Fungi and the Insect–Fungal Complex

Natural *C. sinensis* was introduced to Western countries by the French missionary Dominicus Parennin in 1723. Its intrinsic fungus was identified by Jonathan Pereira in 1843 as belonging to the Sphaeria genus, and the insect portion was identified by Edward Doubleday as belonging to Agrotis in 1842 [95,96,97]. Historical taxonomic examinations of natural *C. sinensis* clearly demonstrated that it is an insect–fungal complex. Miles Joseph Berkeley described the fungus as Sphaeria sinensis Berkeley in 1843 and renamed it Cordyceps sinensis in 1857 [98,99]. Pier Andrea Saccardo renamed it Cordyceps sinensis (Berkeley) Saccardo in 1883 [100,101]. Unfortunately, since then, the Latin name *C. sinensis*, which was originally given to the intrinsic fungus, has been indiscriminately used in academia and the mass market to refer to both the fungus *C. sinensis* and the wild insect–fungal complex.

The fungus was renamed *O. sinensis* in 2007 by Sung et al. [33] with the use of the *H. sinensis* strain EFCC 7287 as the nomenclatural reference [4,7,33,34,35]. Since 2001, >600 ITS1-5.8S-ITS2 sequences of *O. sinensis* have been uploaded to the GenBank database under the GenBank Taxid 72228 [7]. These ITS sequences represent 17 genotypes of *O. sinensis* with numerous, scattered transition, transversion, or insertion/deletion alleles or hereditary variations with reciprocal substitutions of large DNA segments and genetic material recombination [7,9,10,86]. Li et al. [7,10,86] have shown that the 17 genotypes are genomically independent and belong to different *O. sinensis* fungi, which have been postulated to share a common hereditary ancestor [17].

To date, only Genotype #1 of *O. sinensis* (i.e., *H. sinensis*) has been purified and taxonomically characterized [7,102]. Genome-independent Genotypes #2–17 have not been purified, and their taxonomic positions have not been determined [7], although these genotypes are assigned the same Latin name, *O. sinensis*, under the same GenBank taxonomic ID 72228. Controversies, hypotheses, and scientific arguments surrounding genotypic mutations of *O. sinensis* pervade the entire field of *O. sinensis* research.

This historical situation prompted us to embrace the currently available information and use different names, i.e., *H. sinensis*, *O. sinensis*, *C. sinensis* insect–fungal complex, and wild-type *C. sinensis* isolates, to refer to different study materials. This practice might not be perfect because of historical uncertainties in academia. However, we hope that the taxonomic/nomenclatural uncertainties will encourage taxonomists in mycology, botany, and traditional Chinese medicine to design and conduct future studies to solve these historical academic problems.

### 4.2. Correlations of Changes in the Hydrophobic Properties and the Primary, Secondary, and Tertiary Structures of the DNA-Binding Domains of MAT1-1-1 and MAT1-2-1 Proteins

The MATα_HMGbox domain of the MAT1-1-1 protein and the HMG-box_ROX1-like domain of the MAT1-2-1 protein are distinct DNA-binding domains that are critical for fungal mating-type regulation but differ in their evolutionary lineages, structural architectures, and functional roles [103,104,105]. The HMGbox domains in mating proteins are multifunctional motifs that are central to the transcriptional regulation of mating-related genes, *O. sinensis* sexual reproduction, and host adaptation in the *C. sinensis* insect–fungal complex. Our study revealed that various 1–3 amino acid substitutions within the MATα_HMGbox and HMG-box_ROX1-like domains of the MAT1-1-1 and MAT1-2-1 proteins, respectively, of the wild-type *C. sinensis* isolates and *C. sinensis* insect–fungal complexes resulted in changes in the hydrophobicity properties and in the secondary and tertiary structures of the DNA-binding domains of the proteins (Figure 3, Figure 4, Figure 5, Figure 6, Figure 7, Figure 8, Figure 9, Figure 10, Figure 11, Figure 12, Figure 13, Figure 14, Figure 15 and Figure 16). The variable sequences of the DNA-binding domains clustered into different clades in the Bayesian clustering trees (Figure 1 and Figure 2, Tables S6–S9).

The MATα_HMGbox domain of the MAT1-1-1 protein contains a core structure of 3 α-helices that ensures high-affinity, sequence-dependent binding to AT-rich DNA motifs [44,45,46]. This binding facilitates the assembly of the transcriptional machinery in a way that involves mating-type-specific genes and activates gene transcription by inducing DNA conformational changes through physical bending or twisting of the target DNA segments [46,106,107]. This domain directly participates in the promoter recognition of specific genes related to the heterothallic reproductive process [108].

Table S5 shows the hydropathy values for amino acids that are defined by their side chains according to Kyte & Doolittle [67] (https://web.expasy.org/protscale/, accessed from 18 October 2024 to 20 May 2025). Larger hydropathy values indicate greater hydrophobicity, whereas negative values indicate hydrophilicity [67,84,85]. The core α-helical stereostructures of the MATα_HMGbox domains are stabilized through a hydrophobic core that is composed of nonpolar amino acids such as isoleucine (Ile, I), valine (Val, V), leucine (Leu, L), phenylalanine (Phe, F), cysteine (Cys, C), methionine (Met, M), and alanine (Ala, A); these amino acids have hydropathy indices of 4.5, 4.2, 3.8, 2.8, 2.5, 1.9 and 1.8, respectively (Table S5) [67].

The HMG-box_ROX1-like domain of the MAT1-2-1 protein also consists of 3 asymmetrically arranged α-helices that are connected by flexible loops, forming a key hydrophobic core that is stabilized by the presence of the aforementioned nonpolar amino acids (Table S5). The HMG-box_ROX1-like domain of the MAT1-2-1 protein regulates gene expression in a sequence-specific manner by binding to ATTAAT or ATTGTT motifs [103,104,105]. This domain has been implicated in chromatin remodeling and transcriptional repression through its interaction with other corepressors, similar to the repressor of oxygen-regulated genes 1 (ROX1) domain in yeast through pathways opposing to the MATα_HMGbox domain of the MAT1-1-1 protein [104,109,110]. ROX1 represses its own promoter, creating a feedback loop for regulating the hypoxic response as a central hypoxia-responsive regulator that plays roles in chromatin remodeling following DNA binding and in the integration of metabolic signals [111].

To date, no studies have directly addressed heterodimerization between the MAT1-1-1 and MAT1-2-1 proteins of *O. sinensis*, although the formation of heterodimers between the MATα_HMGbox domain of the MAT1-1-1 protein and the HMG-box_ROX1-like domain of the MAT1-2-1 protein is believed to be a critical mechanism regulating fungal mating, particularly in heterothallic ascomycetes [112,113,114]. However, the MATα_HMGbox and HMG-box_ROX1-like domains likely interact with each other, enabling them to cooperate synergistically in DNA binding through their complementary electrostatic surfaces [115]. The heteromorphic stereostructures of the HMGbox domains of the mating protein variants derived from wild-type *C. sinensis* isolates presented in this study likely alter the complementary cooperation of the mating proteins of self-sterile *O. sinensis* under heterothallic or hybrid outcrossing and affect the subsequent functions of the activation or repression of mating-related gene transcription [50,54,55].

### 4.3. Heterogenous Fungal Sources of the MAT1-1-1 and MAT1-2-1 Proteins

The analysis of full-length mating protein variants derived from wild-type *C. sinensis* isolates revealed their heterogeneous fungal components, which belong to genomically independent GC-biased Genotypes #1 and #3 of *O. sinensis* fungi (Table S4), suggesting that the mating protein variants derived from the wild-type *C. sinensis* isolates (Figure 3, Figure 4, Figure 5, Figure 6, Figure 7, Figure 8, Figure 10, Figure 11, Figure 12 and Figure 13) may have different fungal sources [5,7,10,34,36,52,56,57]. In addition to the impure wild-type *C. sinensis* isolates, Li et al. [23] reported 8 heterogeneous *O. sinensis* strains, which are cultures of *C. sinensis* monoascospores that contain both genome-independent GC-biased Genotype #1 and AT-biased Genotype #5 of *O. sinensis* fungi. These *O. sinensis* strains produced MAT1-1-1 and MAT1-2-1 proteins with different truncation mutations and various amino acid substitutions at different mutation sites (the detailed analytical data will be published elsewhere). Whether the impure wild-type *C. sinensis* isolates co-occur with GC-biased Genotype #1 and other heterospecific fungi remains to be determined through the use of culture-dependent or culture-independent protocols involving the use of multiple pairs of PCR primers combined with strict and rigorous amplicon cloning–sequencing techniques.

Li et al. [86] conducted a similar study on other wild-type *C. sinensis* isolates, CH1 and CH2. Through the use of multiple pairs of PCR primers and rigorous amplicon cloning-sequencing techniques, these wild-type isolates were proven to coexist with GC-biased Genotype #1 and AT-biased Genotypes #4–5 of *O. sinensis* and *Paecilomyces hepiali*. Li et al. [7,9,10,54,55] further confirmed that the ITS sequences of Genotypes #2–17 of *O. sinensis* with multiple transition and transversion point mutations are not repetitive genomic copies of GC-biased Genotype #1 *H. sinensis* but apparently belong to independent *O. sinensis* fungi, regardless of whether they exhibit GC or AT biases. These wild-type isolates phenotypically exhibit in vitro growth and microscopic morphological characteristics of psychrophilic *H. sinensis*. Functionally, in the experiments in which the larvae of *Hepialus armoricanus* were inoculated with *H. sinensis* or wild-type *C. sinensis* isolates, CH1 and CH2 (*n* = 100 larvae per study group), the infection rate significantly increased from nearly noninfectious levels of 1–3% for the conidia or mycelia of *H. sinensis* to 55.2% for the wild-type isolates CH1 and CH2, representing an up to 39-fold enhancement (*p* < 0.001), and the larval death latency largely decreased from 35–50 days for *H. sinensis* to 5–8 days for the wild-type isolates. The findings for the wild-type *C. sinensis* isolates suggested that naturally cooccurring fungi may have a synergistic ability increasing the inoculation/infection potency.

### 4.4. The MAT1-1-1 and MAT1-2-1 Proteins Are Involved in the Sexual Reproduction of O. sinensis

The MAT1-1-1 and MAT1-2-1 proteins coregulate mating compatibility and sexual development during the sexual reproduction of *O. sinensis*, a process in which the MATα_HMGbox and HMG-box_ROX1-like domains play key roles. The hypothesis of self-fertilization via a homothallic or pseudohomothallic strategy has been proposed for *O. sinensis* [24,48,49,60] based on genetic research. However, Zhang and Zhang [52] argued against this hypothesis and suggested that *O. sinensis* uses facultative hybridization for sexual reproduction based on the differential occurrence of the *MAT1-1-1* and *MAT1-2-1* genes in more than 170 wild-type *C. sinensis* isolates derived from insect–fungal complex specimens collected from different production areas on the Qinghai–Tibet Plateau. Furthermore, Li et al. [50,54,55] reported the differential occurrence, differential translation, and alternative splicing of *MAT1-1-1* and *MAT1-2-1* and pheromone receptor genes and heteromorphic stereostructures of the MAT1-1-1 and MAT1-2-1 proteins of wild-type *C. sinensis* isolates. In addition, the analysis of DNA-binding domains presented in this study further revealed diverse stereostructures of the MATα_HMGbox domain of the MAT1-1-1 protein and the HMG-box_ROX1-like domain of the MAT1-2-1 protein. These findings suggest that *O. sinensis* experiences self-sterility and uses a heterothallic or hybrid (even parasexuality) strategy to accomplish sexual reproduction during the lifecycle of the *C. sinensis* insect–fungal complex [10,50,54,55,87,88,89,90,91,92,93,94].

Unlike homothallic reproduction, heterothallic or hybrid reproduction of *O. sinensis* requires mating partners. Among the 17 genotypes of *O. sinensis* [7,14,16,17,23,87,116,117,118,119,120,121,122,123], GC-biased Genotype #1 *H. sinensis* has been proposed as the sole anamorph of *O. sinensis* [31]. Wei et al. [32] reported the successful industrial cultivation of *C. sinensis* insect–fungal complexes; however, the cultivated insect–fungal complexes presented a species contradiction between the anamorphic inoculants of 3 *H. sinensis* strains and the sole teleomorph of AT-biased Genotype #4 that was detected in the fruiting body of the cultivated insect–fungal complex. The sequences of AT-biased Genotypes #4–6 and #15–17, as well as the sequences of GC-biased Genotypes #2–3 and #7–14 of *O. sinensis*, do not reside in the genome assemblies ANOV00000000, JAAVMX000000000, LKHE00000000, LWBQ00000000, or NGJJ00000000 of the *H. sinensis* strains Co18, IOZ07, 1229, ZJB12195, and CC1406-20395, respectively [7,8,9,17,23,49,59,60,61,62,121,124]. Li et al. [124] further revealed that the sequences of Genotypes #2–17 of *O. sinensis* did not appear as repetitive copies in the genome of Genotype #1 *H. sinensis*, invalidating the hypothesis proposed by Li et al. [23,24,125] that “RIP mutation” [24] induces or generates “ITS pseudogenes” [23] and “rRNA pseudogenes” [125] in the genome of *H. sinensis*. These data indicate that the 17 genotypes of *O. sinensis* are genomically independent and belong to different fungi. Critically, the data indicate that the sole anamorph hypothesis previously proposed for *H. sinensis* [31] fails to pass the examination of all 4 criteria of Koch’s postulates.

Li et al. [126] revised the inoculant information for industrial cultivation projects and reported that the cultivation project used cultures of *C. sinensis* ascospores as inoculants rather than pure *H. sinensis* strains, as previously reported [32]. However, Li et al. [9,10] reported the detection of GC-biased Genotypes #1 and #14 and AT-biased Genotypes #5–6 and #16, as well as *P. hepiali,* in the ascospores of natural *C. sinensis* insect–fungal complexes through a strict and rigorous culture-independent approach. In addition, Li et al. [23] used a culture-dependent protocol and reported the detection of GC-biased Genotype #1 and AT-biased Genotype #5 in 8 cultures of *C. sinensis* monoascospores. Li et al. [86] observed the nearly noninfectious feature of *H. sinensis* conidia and mycelia on 200 larvae of *H. armoricanus* in inoculation experiments. Furthermore, Li et al. [7,9,10] reported that AT-biased Genotype #4 of *O. sinensis* occurred in the stromata throughout the entire course of *C. sinensis* maturation and that the stromal fertile portion (SFP) contained numerous ascocarps of the *C. sinensis* insect–fungal complex; the abundance of Genotype #4 was high in immature stromata, which are in asexual growth stages, and largely decreased in mature stromata and SFP, which are in sexual production stages or in the transitional stages from asexual growth to sexual reproduction. Unfortunately, AT-biased Genotype #4 of *O. sinensis* was absent from the *C. sinensis* ascospores. Thus, although Wei et al. [32] reported the detection of the sole teleomorphic AT-biased Genotype #4 in the cultivated *C. sinensis* insect–fungal complex and Li et al. [126] later revised the inoculant information, the source of the sole teleomorphic, AT-biased Genotype #4 of *O. sinensis* remains scientifically uncertain in the cultivated *C. sinensis* insect–fungal complex.

Hu et al. [49], Holliday & Cleaver [127], and Stone [128] reported unsuccessful attempts at artificial cultivation of the fruiting bodies and ascospores of *C. sinensis* in academic settings. Faced with the unsuccessful experiment reported by Hu et al. [49], their coauthors Zhang et al. [36] summarized the 40-year history of cultivation failures of insect–fungal complexes using a “pure” mycology strategy in academic research-oriented settings. Qin et al. [129] further analyzed the unsuccessful situation and summarized the obstacles to the cultivation of *O. sinensis* fruiting bodies and ascospores. In contrast, Wei et al. [32] reported success in such a cultivation effort in industrial product-oriented settings. This industrial success might be attributed to the application of a “mycologically impure” cultivation strategy based on at least two facts:

(1) Cultures of mycologically impure ascospores of natural *C. sinensis* are used as inoculants [126]. These cultures are most likely combined with cocultures of *C. sinensis* stroma and/or a stromal fertile portion (SFP) containing numerous ascocarps that, most importantly, contain AT-biased Genotype #4 of *O. sinensis*, which is consistent with the discovery of this genotype as the “sole” teleomorph of *O. sinensis* [32];

(2) Soil collected from natural *C. sinensis* production areas on the Qinghai–Tibet Plateau was added to the industrial cultivation system, as reported by Wei et al. [32].

Unfortunately, the purification and genomic sequencing of GC- and AT-biased Genotypes #2–17 of *O. sinensis* have not been reported to date. Thus, genomic and transcriptomic information regarding mutations in the *MAT1-1-1* and *MAT1-2-1* genes in the genome-independent *O. sinensis* fungi of Genotypes #2–17, especially the DNA-binding domains of the MAT1-1-1 and MAT1-2-1 proteins, is lacking. Table S4 shows that the MAT1-1-1 proteins ALH25057, ALH25005, and ALH25006 and the MAT1-2-1 proteins AIV43040, AFX66443, ACV60417, AFH35020, and ACV60418 can be possibly produced by Genotype #3 of *O. sinensis* if GC-biased Genotype #3 of *O. sinensis* does not coexist with other heterospecific fungi in the wild-type *C. sinensis* isolates XZ12_16, XZ05_8, XZ-LZ07-H1, XZ06-124, and XZ-LZ07-H2, which were derived from insect–fungal complex specimens collected from Tibet.

The synergy of the MAT1-1-1 and MAT1-2-1 proteins of *O. sinensis* constitutes the core mechanism that regulates mating type recognition, triggering downstream nuclear fusion signaling pathways and fruiting body development [39,40,41,42,43]. The differential occurrence, differential transcription, and alternative splicing of mating-type and pheromone receptor genes in *H. sinensis* and wild-type *C. sinensis* isolates disproves the self-fertilization hypothesis at the genomic and transcriptomic levels but suggests self-sterility of *O. sinensis* under heterothallic or hybrid reproduction [31,48,49,50,51,53,54,55]. Furthermore, at the protein level, many MAT1-1-1 and MAT1-2-1 protein variants reported by Li et al. [50] that contain variant MATα_HMGbox and HMG-box_ROX1-like domains, respectively, have been discovered in wild-type *C. sinensis* isolates, and the insect–fungal complex analyzed in this study suggests that mating proteins possibly from heterogeneous fungal sources may accomplish coordinated heterothallic or hybrid reproduction of *O. sinensis* during the lifecycle of the *C. sinensis* insect–fungal complex.

## 5. Conclusions

This study reveals the variations in the primary, secondary, and tertiary structures of the mating proteins in numerous wild-type *C. sinensis* isolates, *H. sinensis* strains, and *C. sinensis* insect–fungal complex specimens, focusing on the DNA-binding domains, i.e., the MATα_HMGbox domain of the MAT1-1-1 protein and the HMG-box_ROX1-like domain of the MAT1-2-1 protein. These DNA-binding domains, with a hydrophobic core formed by 3 α-helices, are crucial for specifically controlling the expression of genes related to the sexual reproduction of *O. sinensis*. The sequences of the DNA-binding domains of the MAT1-1-1 and MAT1-2-1 proteins with various amino acid substitutions at different mutation sites are clustered into 5 and 2 major Bayesian clades, respectively, each encompassing several Bayesian branches, and alter the tertiary protein structures based on AlphaFold-predicted stereostructure models. These findings support the self-sterility hypothesis for *O. sinensis* under heterothallic or hybrid mating at the protein structural level, further complementing the genetic and transcriptional evidence to disprove the self-fertilization hypothesis for *O. sinensis* during homothallic or pseudohomothallic reproduction. Thus, the identification of an appropriate mating partner is the only mechanism by which self-sterile *O. sinensis* can accomplish sexual reproduction during the lifecycle of the *C. sinensis* insect–fungal complex on the Qinghai–Tibet Plateau. This conceptual shift in sexual reproduction mode will aid in the design of future reproductive physiology studies for experimental validation.

## Data Availability

All sequence and 3D structure data are available in public depository databases: GenBank (https://www.ncbi.nlm.nih.gov/genbank/, accessed from 18 October 2024 to 10 July 2025) and AlphaFold (https://alphafold.com/, accessed from 18 October 2024 to 10 November 2025).

## Supplementary Materials

The following supporting information can be downloaded at https://www.mdpi.com/article/10.3390/biology15020186/s1. Figure S1. Alignment of the sequence of the reference MAT1-1-1 protein AGW27560 derived from the *Hirsutella sinensis* strain CS68-2-1229 [48] and the sequences of the variant proteins derived from the wild-type *Cordyceps sinensis* isolates with various amino acid residue substitutions derived from the wild-type *C. sinensis* isolates and from the genome and metatranscriptome assemblies of *H. sinensis* strains or the *C. sinensis* insect–fungal complexes [49,60,62,63,64]. The underlined segment in blue refers to the MATα_HMGbox domain (amino acids 51→225) of the reference MAT1-1-1 protein AGW27560, and the 9 external amino acid residues upstream and downstream of the domain are shown in blue but not underlined. The amino acid substitution is shown in red, whereas the hyphens indicate identical amino acid residues, and the spaces denote unmatched protein sequence gaps. Figure S2. Alignment of the sequence of the reference MAT1-2-1 protein AEH27625 derived from the *Hirsutella sinensis* strain CS2 [56] and the sequences of the variant MAT1-2-1 proteins derived from the wild-type *Cordyceps sinensis* isolates with various amino acid residue substitutions and from the genome and metatranscriptome assemblies of *H. sinensis* strains or the *C. sinensis* insect–fungal complexes [49,58,59,60,61,64]. The underlined segment in blue refers to the HMG-box_ROX1-like domain (127→197 of the reference sequence AEH27625), and the 9 external amino acid residues upstream and downstream of the domain are shown in blue but not underlined. The amino acid substitution is shown in red, whereas the hyphens indicate identical amino acid residues, and the spaces denote unmatched protein sequence gaps. Figure S3. Correlations of the changes in the primary and secondary structures of the MATalpha_HMGbox domains of MAT1-1-1 proteins. The reference protein AGW27560 is derived from the *H. sinensis* strain CS68-2-1229 [48], and the truncated MAT1-1-1 protein is encoded by the metatranscriptome assembly GAGW01008880 derived from the *C. sinensis* insect–fungal complex. Panel (A) shows an alignment of the amino acid sequences in the MATα_HMGbox domains of the MAT1-1-1 proteins; amino acid substitutions are shown in red, whereas the hyphens indicate identical amino acid residues, and the spaces denote unmatched protein sequence gaps. The ExPASy ProtScale plots show the changes in hydrophobicity [Panel (B)] and the 2D structures [Panels (C–F) show the α-helices, β-sheets, β-turns, and coils, respectively]); the open blue rectangles highlight the truncation region in the plots. Table S1. Co-occurrence or differential occurrence of the MAT1-1-1 and MAT1-2-1 proteins detected in different sample sources. Table S2. GenBank accession numbers (in red in parentheses) for the full-length MAT1-1-1 proteins in the AlphaFold database with the corresponding AlphaFold UniProt codes [50]. Table S3. GenBank accession numbers (in red) for the full-length MAT1-2-1 proteins of 69 *H. sinensis* strains or *C. sinensis* isolates with the corresponding AlphaFold UniProt codes [50]. Table S4. Wild-type *C. sinensis* isolates, GenBank accession numbers for the ITS nucleic acid sequences and mating protein sequences, and percentage similarities compared with GC-biased *O. sinensis* Genotypes #1–3 and #7–9. Table S5. Amino acids are scaled based on the general chemical characteristics of their side chains for the ProtScale analysis (https://web.expasy.org/protscale/, accessed from 18 October 2024 to 20 May 2025) to predict the hydrophobicity and secondary structures (α-helices, β-sheets, β-turns, and coils) of proteins. Table S6. Summary of the results shown in Figure 1, Figure 3, Figure 4, Figure 5, Figure 6, Figure 7, Figure 8 and Figure S1 with the amino acid substitutions in the MATα_HMGbox domains of the 19 full-length MAT1-1-1 proteins of the wild-type *C. sinensis* isolates based on AlphaFold UniProt codes and GenBank accession numbers. Table S7. Summary of the Bayesian clustering results shown in Figure 1, Figure 9, Figure S1, and Figure S3 with the amino acid substitutions in the MATα_HMGbox domains of the MAT1-1-1 proteins encoded by the genome assemblies of *H. sinensis* strains and the metatranscriptome assemblies of natural *C. sinensis* based on the GenBank accession numbers. Table S8. Summary of the results shown in Figure 2, Figure 10, Figure 11, Figure 12, Figure 13 and Figure S2 with the amino acid substitutions in the HMG-box_ROX1-like domains of the 25 full-length MAT1-2-1 proteins of wild-type *C. sinensis* isolates based on the AlphaFold UniProt codes and GenBank accession numbers. Table S9. Summary of the results shown in Figure 2, Figure 14, Figure 15, Figure 16 and Figure S2 with the amino acid substitutions and deletions in the HMG-box_ROX1-like domains of the MAT1-2-1 proteins encoded by the genome and transcriptome assemblies of *H. sinensis* strains and the metatranscriptome assembly of natural *C. sinensis* insect-fungal complexes found based on the GenBank accession numbers.
