# Supplementary material for: Altered Stereostructures of the DNA-Binding Domains of Variant Mating Proteins of Ophiocordyceps sinensis and the Wild Insect–Fungal Complex
Source: Biology (Basel). 2026 Jan 19;15(2):186. doi: 10.3390/biology15020186 (PMC12837857; doi:10.3390/biology15020186)
Supplement: Supplementary file 1 [file biology-15-00186-s001.zip › biology-4031874-supplementary.pdf]

Altered Stereostructures of the DNA-Binding Domains of Variant Mating Proteins of *Ophiocordyceps sinensis* and the Wild Insect–Fungal Complex

Xiu-Zhang Li [1], Yu-Ling Li [1], Wei Liu [2] and Jia-Shi Zhu [1,*]

[1] State Key Laboratory of Plateau Ecology and Agriculture, Qinghai Academy of Animal and Veterinary Sciences, Qinghai University, Xining 810016, China; xiuzhang11@163.com (X.-Z.L.); yulingli2000@163.com (Y.-L.L.)
[2] Institute of Immunology, Army Medical University, Chongqing 400038, China; weiliu@tmmu.edu.cn
* Correspondence: zhujosh@163.com

```
AGW27560          1          MTTRNEVMQRLSSVRADVLLNFLTDDAIFQLASRYHESTTEADVLTPVSTAAASRATRQT 60
EQK97643          1          ------------------------------------------------------------ 60
ALH25054          1          ------------------------------------------------------------ 60
ALH24951          1          ------------------------------------------------------------ 60
ALH24992          1          --------------------------------------------------T--------- 60
ALH24999          1          ------------T----I--------------------------------V----T---- 60
ALH25057          1          ------------T----I--------------------------------V----T---- 60
ALH25001          1          ------------T----I--------------------------------V----T---- 60
ALH25005          1          ------------------------------------------------------------ 60
ALH25043          1          ------------------------------------------------------------ 60
ALH25045          1          ------------------------------------------------------------ 60
ALH25046          1          ------------------------------------------------------------ 60
ALH25048          1          ------------------------------------------------------------ 60
ALH25047          1          ------------------------------------------------------------ 60
ALH25003          1          ------------------------------------------------------------ 60
ALH24946          1          --------K--------------------------Y------------------------ 60
ALH24948          1          ------------------------------------------------------------ 60
ANOV01017390      1519       ------------------------------------------------------------ 1340
LKHE01001116      4909       ------------------------------------------------------------ 4730
JAAVMX010000001   6,698,911  ------------------------------------------------------------ 6,699,090
GAGW01008880
OSIN7648          1          ------------------------------------------------------------ 181

AGW27560          61         KEASCDRAKRPLNAFMAFRSYYLKLFPDVQQKTASGFLTTLWHKDPFRNKWALIAKVYSF 120
EQK97643          61         ------------------------------------------------------------ 120
ALH25054          61         ------------------------------------------------------------ 120
ALH24951          61         ------------------------------------------------------------ 120
ALH24992          61         ------------------------------------------------------------ 120
ALH24999          61         ------------------------------------------------------------ 120
ALH25057          61         ------------------------------------------------------------ 120
ALH25001          61         ------------------------------------------------------------ 120
ALH25005          61         ------------------------------------------------------------ 120
ALH25043          61         ------------------------------------------------------------ 120
ALH25045          61         ------------------------------------------------------------ 120
ALH25046          61         ------------------------------------------------------------ 120
ALH25048          61         ------------------------------------------------------------ 120
ALH25047          61         ------------------------------------------------------------ 120
ALH25003          61         ------------------------------------------------------------ 120
ALH24946          61         ------------------------------------------------------------ 120
ALH24948          61         ------------------------------------------------------------ 120
ANOV01017390      1339       ------------------------------------------------------------ 1109
LKHE01001116      4729       --------------------M--------------------------------------- 4541
JAAVMX010000001   6,699,091  --------------------M--------------------------------------- 6,699,279
GAGW01008880      1127                                           ------------------------ 1020
OSIN7648          182        ------------------------------------------------------------ 360

AGW27560          121        VRDQIGKDKVSLSYFMSLACPTMTIIEPAAYLNALGWCVQDDDAGSQKLFQDESSANLDQ 180
EQK97643          121        ------------------------------------------------------------ 180
ALH25054          121        ----L------------------------------------------------------- 180
ALH24951          121        ----L------------------------------------------------------- 180
ALH24992          121        ------------------------------------------------------------ 180
ALH24999          121        ------------------------------------------------------------ 180
ALH25057          121        ------------------------------------------------------------ 180
ALH25001          121        ------------------------------------------------------------ 180
ALH25005          121        ------------------------------------------------------------ 180
ALH25043          121        ------------------------------------------------------------ 180
ALH25045          121        ------------------------------------------------------------ 180
ALH25046          121        ------------------------------------------------------------ 180
ALH25048          121        ------------------------------------------------------------ 180
ALH25047          121        ------------------------------------------------------------ 180
ALH25003          121        ------------------------------------------------------------ 180
ALH24946          121        ------------------------------------------------------------ 180
ALH24948          121        ---SF------------------------------------------------------- 180
ANOV01017390      1108       ------------------------------------------------------------ 929
LKHE01001116      4540       ------------------------------------------------------------ 4318
JAAVMX010000001   6,699,280  ------------------------------------------------------------ 6,699,502
GAGW01008880      1019       ------------------------------------------------------------ 840
OSIN7648          361        ------------------------------------------------------------ 541
```

```
AGW27560         181        SSLLSAEYPSTEIELLSALVNIGYFPDHGADLVERMGSSHSGIMAPRAANCTPPVSYTKE 240
EQK97643         181        ------------------------------------------------------------ 240
ALH25054         181        ------------------------------------------------------------ 240
ALH24951         181        ------------------------------------------------------------ 240
ALH24992         181        ------------------------------------------------------------ 240
ALH24999         181        ------------------------------------------------------------ 240
ALH25057         181        ------------------------------------------------------------ 240
ALH25001         181        ------------------------------------------------------------ 240
ALH25005         181        ------------------------------------------------------------ 240
ALH25043         181        ----------------------------------I----------T-------------- 240
ALH25045         181        ----------------------------------I----------T-------------- 240
ALH25046         181        ----------------------------------I----------T-------------- 240
ALH25048         181        ----------------------------------I----------T-------------- 240
ALH25047         181        ------------------------------------------------------------ 240
ALH25003         181        --------------------------------------G--------------------- 240
ALH24946         181        ------------------------------------------------------------ 240
ALH24948         181        ------------------------------------------------------------ 240
ANOV01017390     928        ------------------------------------------------------------ 749
LKHE01001116     4317       ------------------------------------------------------------ 4138
JAAVMX010000001  6,699,503  ------------------------------------------------------------ 6,699,682
GAGW01008880     839        ------------------------------------------------------------ 660
OSIN7648         542        ------------------------------------------------------------ 719

AGW27560         241        KIDFINTIRSDPVQATKEILGDCYDETTIKLLGVKSHNVESVDSITHLSMQREYQAPRFF 300
EQK97643         241        ------------------------------------------------------------ 300
ALH25054         241        -----------------------------------------------------H------ 300
ALH24951         241        ------------------------------------------------------------ 300
ALH24992         241        ------------------------------------------------------------ 300
ALH24999         241        -V---------------------------------------------------------- 300
ALH25057         241        -V---------------------------------------------------------- 300
ALH25001         241        -------------------------K---------------------------H------ 300
ALH25005         241        -V---------------------------------------------------------- 300
ALH25043         241        ------I----------------------------------------------------- 300
ALH25045         241        ------I----------------------------------------------------- 300
ALH25046         241        ------I----------------------------------------------------- 300
ALH25048         241        ------I----------------------------------------------------- 300
ALH25047         241        -----------------------------------------L------------------ 300
ALH25003         241        -------------------------K---------------------------H------ 300
ALH24946         241        ------------------------------------------------------------ 300
ALH24948         241        ------------------------------------------------------------ 300
ANOV01017390     748        ------------------------------------------------------------ 569
LKHE01001116     4137       ------------------------------------------------------------ 3958
JAAVMX010000001  6,699,683  ------------------------------------------------------------ 6,699,862
GAGW01008880     659        ------------------------------------------------------------ 480
OSIN7648         720        -----------------------------------------------F------------ 863

AGW27560         301        YDYSVSYAGMDFGGSNEPVMNLNNLPENETFDIDSPFDLDKILGQSQSEGERTSHLPPSP 360
EQK97643         301        ------------------------------------------------------------ 360
ALH25054         301        -------V---------------------------------------------------- 360
ALH24951         301        -------V---------------------------------------------------- 360
ALH24992         301        ------------------------------------------------------------ 360
ALH24999         301        -------------------------H---------------------------------- 360
ALH25057         301        -------------------------H---------------------------------- 360
ALH25001         301        ------------------------------------------------------------ 360
ALH25005         301        -------------------------H---------------------------------- 360
ALH25043         301        ------------A----------------------------------------------- 360
ALH25045         301        ------------A----------------------------------------------- 360
ALH25046         301        ------------A----------------------------------------------- 360
ALH25048         301        ------------A----------------------------------------------- 360
ALH25047         301        -----------------------------------------------L------------ 360
ALH25003         301        ------------------------------------------------------------ 360
ALH24946         301        ------------------------------------------------------------ 360
ALH24948         301        ------------------------------------------------------------ 360
ANOV01017390     568        ----------------------------------------------------S        410
LKHE01001116     3957       ----------------------------------------------------S        3799
JAAVMX010000001  6,699,863  ----------------------------------------------------S        6,700,021
GAGW01008880     479        ------------------------------------------------------------ 336
OSIN7648         864              ------------------------------------------------------ 1027
```

AGW27560	361	PHNPLDDFYFAF	372
EQK97643	361	------------	372
ALH25054	361	------------	372
ALH24951	361	------------	372
ALH24992	361	------------	372
ALH24999	361	-Y----------	372
ALH25057	361	-Y----------	372
ALH25001	361	------------	372
ALH25005	361	-Y----------	372
ALH25043	361	------------	372
ALH25045	361	------------	372
ALH25046	361	------------	372
ALH25048	361	------------	372
ALH25047	361	------------	372
ALH25003	361	------------	372
ALH24946	361	------------	372
ALH24948	361	------------	372
ANOV01017390	410		
LKHE01001116	3799		
JAAVMX010000001	6,700,021		
GAGW01008880	335	------------	300
OSIN7648	1028	------------	1065

Figure S1. Alignment of the sequence of the reference MAT1-1-1 protein AGW27560 derived from the *Hirsutella sinensis* strain CS68-2-1229 [48] and the sequences of the variant proteins derived from the wild-type *Cordyceps sinensis* isolates with various amino acid residue substitutions derived from the wild-type *C. sinensis* isolates and from the genome and metatranscriptome assemblies of *H. sinensis* strains or the *C. sinensis* insect–fungal complexes [49,60,62–64]. The underlined segment in blue refers to the MATα_HMGbox domain (amino acids 51→225) of the reference MAT1-1-1 protein AGW27560, and the 9 external amino acid residues upstream and downstream of the domain are shown in blue but not underlined. The amino acid substitution is shown in red, whereas the hyphens indicate identical amino acid residues, and the spaces denote unmatched protein sequence gaps.

```
AEH27625     1       MANPINMIPNPQWNATDYEAIWKGLEAQVNPFSQILCLEGDFFRQLDDAAKLFIARKLME 60
EQL04085     1       ------------------------------------------------------------ 60
AGW27537     1       ------------------------------------------------------------ 60
AGW27539     1       ------------------------------------------------------------ 60
AGW27553     1       ------------------------------------------------------------ 60
AFX66401     1       ----------------------------------------I------------------- 60
AFX66442     1       ------------------------------------------------------------ 60
AFX66437     1       ----------------------------------------N------------------- 60
AFX66472     1       -----------------------------------------------G------------ 60
AFX66476     1       -----------------------------------------------G------------ 60
AFX66475     1       ----------------------------------T------------------------- 60
AFX66484     1       ------------------------------------------------------------ 60
ACV60385     1       ----------------------------------V------------------------- 60
ACV60363     1       ------------------------------------------------------------ 60
ACV60372     1       ---------------------------------L-------------------------- 60
ACV60391     1       ------------------------------------------------------------ 60
AFX66401     1       ----------------------------------------I------------------- 60
ACV60399     1       ------------------------------------------------------------ 60
ACV60415     1       ------------------------------------------------------------ 60
ACV60417     1       ---------------I-------------------------------------------- 60
AIV43040     1       ---------------I-------------------------------------------- 60
ANOV01000063 9329    ------------------------------------------------------------ 9508
LWBQ01000021 239,726 ------------------------------------------------------------ 239,547
NGJJ01000619 23,883  ------------------------------------------------------------ 23,704
LKHE01001605 14,713  ------------------------------------------------------------ 14,534
GCQL01020543 1143    ------------------------------------------------------------ 964
OSIN7649     1       ------------------------------------------------------------ 60

AEH27625     61      HVQESVLYVNDGNGPDRVYLGAPRHFVVGGGMILQISGYAPYWIRRSVSKVVTATVLAPP 120
EQL04085     61      ------------------------------------------------------------ 120
AGW27537     61      -----------------I------------------------------------------ 120
AGW27539     61      ------------------------------------------------------------ 120
AGW27553     61      ------------------------------------------------------------ 120
AFX66401     61      ------------------------------------------------------------ 120
AFX66442     61      ------------------------------------------------------------ 120
AFX66437     61      ------------------------------------------------------------ 120
AFX66472     61      -----------------------------------------------A------------ 120
AFX66476     61      -----------------------------------------------A------------ 120
AFX66475     61      ------------------------------------------------------------ 120
AFX66484     61      ------------------------------------------------------------ 120
ACV60385     61      ------------------------------------------------------------ 120
ACV60363     61      ------------------------------------------------------------ 120
ACV60372     61      ------------------------------------------------------------ 120
ACV60391     61      ------------------------------------------------------------ 120
AFX66401     61      ------------------------------------------------------------ 120
ACV60399     61      ------------------------------------------------------------ 120
ACV60415     61      ------------------------------------------------------------ 120
ACV60417     61      ------------------------------------------------------------ 120
AIV43040     61      ------------------------------------------------------------ 120
ANOV01000063 9558    ------------------------------------------------------------ 9740
LWBQ01000021 239,506 ------------------------------------------------------------ 239,315
NGJJ01000619 23,666  ------------------------------------------------------------ 23,472
LKHE01001605 14,496  ------------------------------------------------------------ 14,302
GCQL01020543 963     ------------------------------------------------------------ 784
OSIN7649     61      ------------------------------------------------------------ 120

AEH27625     121     SPKDIKIPRPPNAYILYRKERHHYVKDANPGITNNEISQILGKAWNMESNDVRQKYKDMS 180
EQL04085     121     ------------------------------------------------------------ 180
AGW27537     121     -----------------------H------------------------------------ 180
AGW27539     121     ------------------------------------------------------------ 180
AGW27553     121     -----------------------H------------------------------X----- 180
AFX66401     121     -----------------------H------------------------------------ 180
AFX66442     121     ------------------------------------------------------------ 180
AFX66437     121     ------------------------------------------------------------ 180
AFX66472     121     -----------------------H------------------------------------ 180
AFX66476     121     -----------------------H------------------------------------ 180
AFX66475     121     -----------------------H------------------------------------ 180
AFX66484     121     -----------------------H------------------------------------ 180
ACV60385     121     -----------------------H------------------------------------ 180
ACV60363     121     -----------------------H------------------------------------ 180
ACV60372     121     ------------------------------------------------------------ 180
ACV60391     121     ------------------------------------------------------------ 180
AFX66401     121     -----------------------H------------------------------------ 180
ACV60399     121     -----------------------H------------------------------------ 180
ACV60415     121     -----------------------H----------------------I------------- 180
ACV60417     121     -----------------------H----------------------I------------- 180
AIV43040     121     -----------------------H----------------------I------------- 180
ANOV01000063 9741    -------------------------------------A---------------------- 9974
LWBQ01000021 239,314 -----------------------H-------------A---------------------- 239,081
NGJJ01000619 23,471  -----------------------H-------------A---------------------- 23,238
LKHE01001605 14,301  -----------------------H-------------A---------------------- 14,068
GCQL01020543 783     -----------------------H------------------------------------ 604
OSIN7649     121     -----------------------H------------------------------------ 180
```

```
AEH27625      181     QQVKQALLEKHPDYQYKPRRPCERRRRRRASPNQNPKQSTSRNAATRDAAISSEDTSTAT 240
EQL04085      181     ------------------------------------------------------------ 240
AGW27537      181     ------------------------------------------------------------ 240
AGW27539      181     ------------------------------------------------------------ 240
AGW27553      181     ------------------------------------------------------------ 240
AFX66401      181     ------------------------------------------------------------ 240
AFX66442      181     ------------------------------------------------------------ 240
AFX66437      181     ------------------------------------------------------------ 240
AFX66472      181     -----------------------------T-----------------N----T------- 240
AFX66476      181     ------------------------------------------------------------ 240
AFX66475      181     ------------------------------------------------------------ 240
AFX66484      181     --------------------------------------------T--------------- 240
ACV60385      181     ------------------------------------------------------------ 240
ACV60363      181     ------------------------------------------------------------ 240
ACV60372      181     ------------------------------------------------------------ 240
ACV60391      181     ------------------------------------------------------------ 240
AFX66401      181     ------------------------------------------------------------ 240
ACV60399      181     --------------R--------------------------------------------- 240
ACV60415      181     ---------------------------------------G-------------------- 240
ACV60417      181     ---------------------------------------A------------T-----G- 240
AIV43040      181     ----------------------------------------------------T-----G- 240
ANOV01000063  9975    ------------------------------------------------------------ 10,155
LWBQ01000021  239,148 ------------------------------------------------------------ 238,900
NGJJ01000619  23,237  ------------------------------------------------------------ 23,057
LKHE01001605  14,067  ------------------------------------------------------------ 13,887
GCQL01020543  603     ------------------------------------------------------------ 424
OSIN7649      181     ------------------------------------------------------------ 240

AEH27625      241     GDTNTANGF                                                    249
EQL04085      241     ---------                                                    249
AGW27537      241     ---------                                                    249
AGW27539      241     ---------                                                    249
AGW27553      241     ---------                                                    249
AFX66401      241     ---------                                                    249
AFX66442      241     ---------                                                    249
AFX66437      241     ---------                                                    249
AFX66472      241     ---------                                                    249
AFX66476      241     ---------                                                    249
AFX66475      241     ---------                                                    249
AFX66484      241     ---------                                                    249
ACV60385      241     ---------                                                    249
ACV60363      241     ---------                                                    249
ACV60372      241     ---------                                                    249
ACV60391      241     ---------                                                    249
AFX66401      241     ---------                                                    249
ACV60399      241     ---------                                                    249
ACV60415      241     ---------                                                    249
ACV60417      241     ---------                                                    249
AIV43040      241     ---------                                                    249
ANOV01000063  10,156  ---------                                                    10,182
LWBQ01000021  238,901 ---------                                                    238,873
NGJJ01000619  23,056  ---------                                                    23,030
LKHE01001605  13,886  ---------                                                    13,860
GCQL01020543  423     ---------                                                    397
OSIN7649      241     ---------                                                    249
```

Figure S2. Alignment of the sequence of the reference MAT1-2-1 protein AEH27625 derived from the *Hirsutella sinensis* strain CS2 [56] and the sequences of the variant MAT1-2-1 proteins derived from the wild-type *Cordyceps sinensis* isolates with various amino acid residue substitutions and from the genome and metatranscriptome assemblies of *H. sinensis* strains or the *C. sinensis* insect–fungal complexes [49,58–61,64]. The underlined segment in blue refers to the HMG-box_ROX1-like domain (127→197 of the reference sequence AEH27625), and the 9 external amino acid residues upstream and downstream of the domain are shown in blue but not underlined. The amino acid substitution is shown in red, whereas the hyphens indicate identical amino acid residues, and the spaces denote unmatched protein sequence gaps.

Panel A: Amino acid sequence alignment

```
AGW27560      42   ADVLTPVSTAAASRATRQTKEASCDRAKRPLNAFMAFRSYYLKLFPDVQQKTASGFLTTL 101
GAGW01008880 1127                                                       ----- 1113

AGW27560      102  WHKDPFRNKWALIAKVYSFVRDQIGKDKVSLSYFMSLACPTMTIIEPAAYLNALGWCVQD 161
GAGW01008880 1112  ------------------------------------------------------------ 933

AGW27560      162  DDAGSQKLFQDESSANLDQSSLLSAEYPSTEIELLSALVNIGYFPDHGADLVERMGSSHS 221
GAGW01008880 932   ------------------------------------------------------------ 753

AGW27560      222  GIMAPRAANCTPP                                                234
GAGW01008880 752   -------------                                                714
```

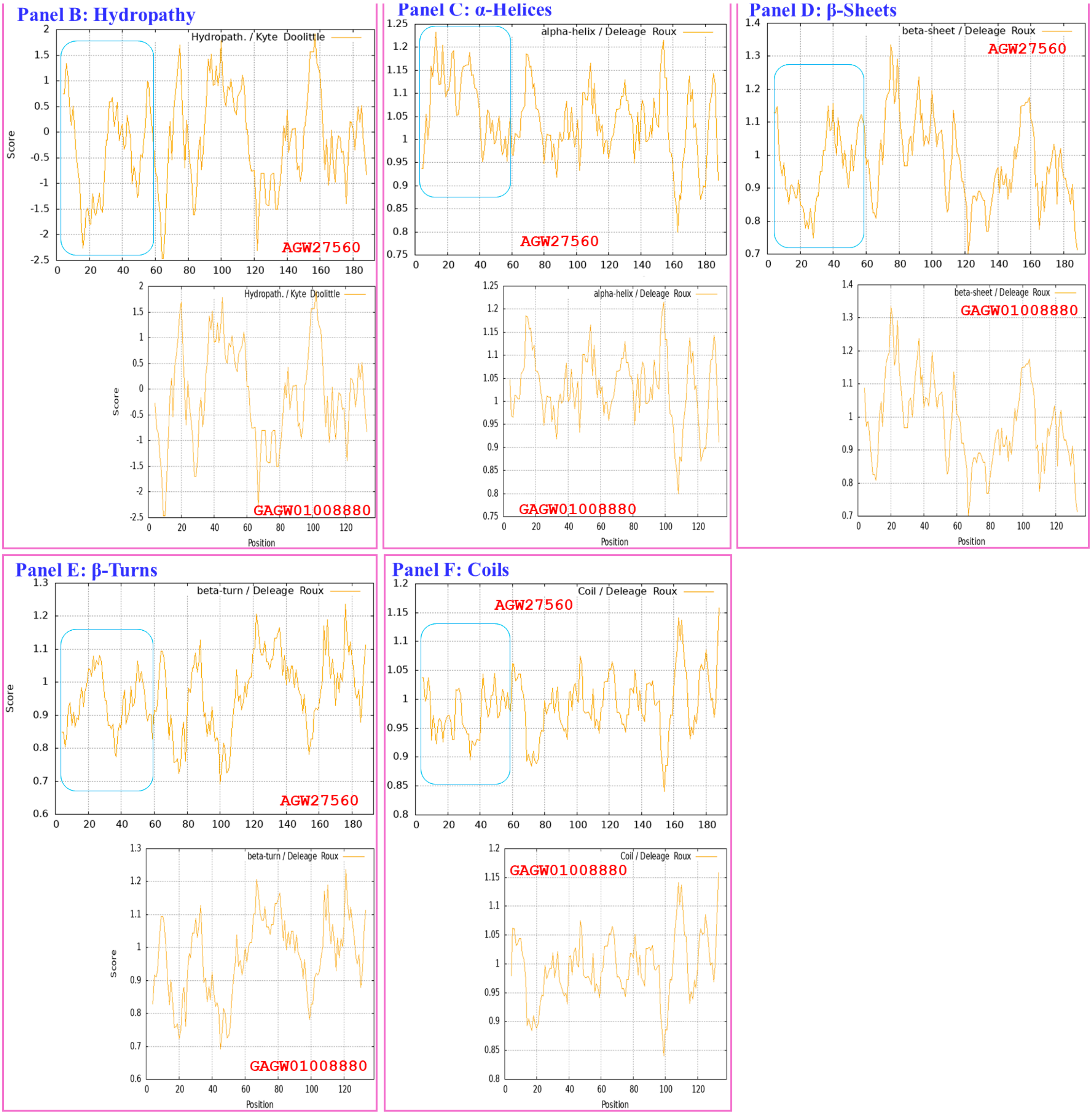

Figure S3. Correlations of the changes in the primary and secondary structures of the MATalpha_HMGbox domains of MAT1-1-1 proteins. The reference protein AGW27560 is derived from the *H. sinensis* strain CS68-2-1229 [48], and the

truncated MAT1-1-1 protein is encoded by the metatranscriptome assembly GAGW01008880 derived from the *C. sinensis* insect–fungal complex. Panel (A) shows an alignment of the amino acid sequences in the MATα_HMGbox domains of the MAT1-1-1 proteins; amino acid substitutions are shown in red, whereas the hyphens indicate identical amino acid residues, and the spaces denote unmatched protein sequence gaps. The ExPASy ProtScale plots show the changes in hydrophobicity [Panel (B)] and the 2D structures (Panels (C–F) show the α-helices, β-sheets, β-turns, and coils, respectively); the open blue rectangles highlight the truncation region in the plots.

Table S1. Co-occurrence or differential occurrence of the MAT1-1-1 and MAT1-2-1 proteins detected in different sample sources.

	Number of samples	Cooccurrence of MAT1-1-1 and MAT1-2-1 proteins	Differential occurrence of mating protein	
			MAT1-1-1	MAT1-2-1
O. sinensis strains of different genotypes, including *H. sinensis* (Genotype #1 of *O. sinensis*)	27	13	10	4
Wild-type *C. sinensis* isolates	151	31	85	35
C. sinensis insect–fungal complexes	5	2	1	2

Table S2. GenBank accession numbers (in red in parentheses) for the full-length MAT1-1-1 proteins in the AlphaFold database with the corresponding AlphaFold UniProt codes [50].

AlphaFold UniProt code	Bayesian cluster-branch	Strain/isolate number (GenBank accession number for MAT1-1-1 protein)
U3N942	**A1**	**GS09_111 (ALH24945), CS68-2-1229 (AGW27560)**, GS09_131 (ALH24947), ID10_1 (ALH24954), IOZ07 (KAF4512729), NP10_1 (ALH24955), NP10_2 (ALH24956), QH07_188 (ALH24957), QH07_197 (ALH24958), QH09_122 (ALH24959),QH09_131 (ALH24960), QH09_151 (ALH24961), QH09_20L (ALH24965), QH09_33L (ALH24967), QH09_37 (ALH24968), QH09_46 (ALH24969), QH09_56 (ALH24970), QH09_66 (ALH24971), QH09_78 (ALH24972), QH09_93 (ALH24973), QH10_1 (ALH24974), QH10_4 (ALH24975), QH10_7 (ALH24976), SC09_107 (ALH24978), SC09_117 (ALH24979), SC09_128 (ALH24980), SC09_147 (ALH24981), SC09_157 (ALH24982), SC09_167 (ALH24983), SC09_180 (ALH24984), SC09_190 (ALH24985), SC09_200 (ALH24986), SC09_21 (ALH24987), SC09_36 (ALH24988), SC09_37 (ALH24989), SC09_47 (ALH24990), SC09_57 (ALH24991), SC09_77 (ALH24993), SC10_18 (ALH24996), SC10_21 (ALH24997), SC10_4 (ALH24998), XZ05_12 (ALH25000), XZ05_3 (ALH25002), XZ05_7 (ALH25004), XZ06_124 (ALH25006), XZ06_152 (ALH25007), XZ07_108 (ALH25009), XZ07_133 (ALH25010), XZ07_154 (ALH25011), XZ07_166 (ALH25012), XZ07_176 (ALH25013), XZ07_180 (ALH25014), XZ08_10 (ALH25015), XZ08_24 (ALH25016), XZ08_26 (ALH25017), XZ08_4 (ALH25018), XZ08_56 (ALH25019), XZ08_59 (ALH25020), XZ08_A1 (ALH25021), XZ08_B1 (ALH25022), XZ09_106 (ALH25024), XZ09_113 (ALH25025), XZ09_118 (ALH25026), XZ09_15 (ALH25027), XZ09_32 (ALH25028), XZ09_4 (ALH25029),

		XZ09_46 (ALH25030), XZ09_48 (ALH25031), XZ09_59 (ALH25032), XZ09_71 (ALH25033), XZ09_80 (ALH25055), XZ10_15 (ALH25035), XZ10_17 (ALH25036), XZ10_23 (ALH25037), XZ10_7 (ALH25038), XZ12_1 (ALH25056), XZ12_33 (ALH25058), XZ12_43 (ALH25059), YN07_6 (ALH25039), YN07_8 (ALH25040), YN09_101 (ALH25041), YN09_140 (ALH25042), YN09_3 (ALH25044), YN09_72 (ALH25049), YN09_81 (ALH25050), YN09_85 (ALH25051), YN09_89 (ALH25052), YN09_96 (ALH25053)
A0A0N9QMM1	**A1**	GS09_121 (ALH24946), GS09_201 (ALH24949), GS09_225 (ALH24950), SC09_1 (ALH24977)
T5A511	**A1**	**Co18 (EQK97643)** (KE657544 410←1519) (ANOV01017390 410←1519)
A0A0N9R5B3	**A2**	SC09_65 (ALH24992)
A0A0N7G849	**A2**	SC09_97 (ALH24995)
A0A0N9QUF3	**A3**	GS09_143 (ALH24948)
A0A0N9R4V2	**A3**	YN09_61 (ALH25047)
A0A0N9QMS9	**B**	YN09_22 (ALH25043), YN09_51 (ALH25045), YN09_6 (ALH25046), YN09_64 (ALH25048)
A0A0N7G845	**C**	GS09_229 (ALH24951), GS09_281 (ALH24952), GS09_311 (ALH25054), GS10_1 (ALH24953), QH09_164 (ALH24962), QH09_173 (ALH24963), QH09_201 (ALH24964), QH09_210 (ALH24966) SC09_87 (ALH24994)
A0A0N9QUK2	**D1**	XZ05_8 (ALH25005)
A0A0N9QMT4	**D2**	XZ07_H2 (ALH24999), XZ12_16 (ALH25057)
A0A0N9QMR3	**E1**	XZ06_260 (ALH25008), XZ09_100 (ALH25023)
A0A0N9QMS4	**E2**	XZ09_95 (ALH25034)
A0A0N7G850	**E3**	XZ05_6 (ALH25003)
A0A0N9R4Q4	**E4**	XZ05_2 (ALH25001)

Note: *, Branch 1 in red, Branch 2 in pink, Branch 3 in purple, and Branch 4 in brown under the cluster codes (English letters) in the parentheses were determined *via* the Bayesian analysis shown in Figure 1 of [50]. The "←" arrows indicate sequences in the antisense strands of the genome of the *H. sinensis* strain Co18.

Table S3. GenBank accession numbers (in red) for the full-length MAT1-2-1 proteins of 69 *H. sinensis* strains or *C. sinensis* isolates with the corresponding AlphaFold UniProt codes [50].

AlphaFold UniProt code	Bayesian cluster-branch	Strain/isolate number (GenBank accession number for MAT1-2-1 protein)
D7F2E9	**I-1**	**CS2 (AEH27625)** (ACV60400), SC-2 (ACV60395), SC-4 (ACV60396), SC-5 (ACV60398), SC-7 (ACV60397), XZ-LZ06-1 (ACV60369), XZ-LZ06-108 (ACV60373), XZ-LZ06-21 (ACV60371), XZ-LZ06-7 (ACV60370), XZ-LZ07-108 (ACV60379),XZ-LZ07-30 (ACV60377), XZ-ML-191 (ACV60376), YN-1 (ACV60390), YN-5 (ACV60392), YN-6 (ACV60393), YN-8 (ACV60394), SC09_47 (AFX66423), SC09_57 (AFX66424), SC09_77 (AFX66426), SC09_97 (AFX66428), XZ05_12 (AFX66444), XZ05_7 (AFX66442), XZ06_152 (AFX66445), XZ07_11 (AFX66447), XZ07_46 (AFX66448), XZ09_106 (AFX66464), XZ09_113 (AFX66465), XZ09_15 (AFX66455), YN09_101 (AFX66482), YN09_72 (AFX66477), YN09_81 (AFX66478), YN09_85 (AFX66479), YN09_89 (AFX66480), SC09-37 (AFH35019), CS26-277 (AGW27541), CS36-1294 (AGW27538), CS37-295 (AGW27539)
T5AF56	**I-1**	**Co18 (EQL04085)** (ANOV01000063 9329→10182)
V9LW10	**I-2**	SC09_200 (AFX66437)
D7F2H1	**I-2**	YN-4 (ACV60391)
D7F2F2	**I-2**	XZ-LZ06-61 (ACV60372)
A0A0A0RCF5	**II-1**	XZ12_16 (AIV43040)
D7F2J7	**II-2**	XZ-LZ07-H1 (ACV60417), XZ-LZ07-H2 (ACV60418), XZ06-124 (AFH35020), XZ05_8 (AFX66443)
D7F2F5	**III**	XZ-LZ05-6 (ACV60415), XZ-SN-44 (ACV60375), XZ05_2 (AFX66441), XZ06_260 (AFX66446), XZ09_100 (AFX66463), XZ09_80 (AFX66461), XZ09_95 (AFX66462)
V9LWC9	**IV-1**	YN09_64 (AFX66476)
V9LVS8	**IV-2**	YN09_6 (AFX66472), YN09_22 (AFX66473), YN09_51 (AFX66474)
D7F2E3	**V-1**	XZ-NQ-154 (ACV60363), XZ-NQ-155 (ACV60364), GS09_111 (AFX66388), QH09-93 (AFH35018), CS560-961 (AGW27542)
D7F2G5	**V-2**	QH-YS-199 (ACV60385)
D7F2H9	**V-2**	SC-3 (ACV60399)
V9LW71	**V-2**	QH09_11 (AFX66401)
V9LVU8	**V-2**	YN09_61 (AFX66475)
V9LWG5	**V-2**	ID10_1 (AFX66484)
U3N6V5	**V-2**	CS6-251 (AGW27537)
‡	**V-1**	NP10_1 (AFX66485), NP10_2 (AFX66486), YN09_3 (AFX66471), YN09_96 (AFX66481), YN09_140 (AFX66483)

Note: **, Branch 1 in red and Branch 2 in pink under the cluster codes (Roman numerals) in the parentheses were determined *via* the Bayesian analysis shown in Figure 2 of [50]. ‡, The 5 MAT1-2-1 protein sequences in green are included in the GenBank database but not in the AlphaFold database. The "→" arrow indicates the sequence in the sense strand of the genome of the *H. sinensis* strain Co18. The "→" arrow indicates the sequence in the sense strand of the genome of the *H. sinensis* strain Co18.

Table S4. Wild-type *C. sinensis* isolates, GenBank accession numbers for the ITS nucleic acid sequences and mating protein sequences, and percentage similarities compared with GC-biased *O. sinensis* Genotypes #1–3 and #7–9.

**Wild-type *C. sinensis* isolate**	**GenBank accession #**			**% similarity *vs.* GC-biased *O. sinensis* genotype**						
	**ITS1-5.8S-ITS2**	**Mating protein**								
		**MAT1-1-1**	**MAT1-2-1**	**#1**	**#2**	**#3**	**#7**	**#8**	**#9**	**#10**
GS09_143	JQ325056	ALH24948	AFX66391	100%	95.0%	88.8%	89.5%	95.2%	83.1%	83.1%
SC09_65	JQ325090	ALH24992	AFX66425	100%	95.0%	88.8%	89.5%	95.2%	83.1%	83.1%
GS09_111	JQ325053	ALH24945	AFX66388	100%	97.2%	95.0%	95.0%	89.5%	95.2%	83.1%
QH09_11	JQ325066		AFX66401	100%	97.2%	95.0%	95.0%	89.5%	95.2%	83.1%
QH09-93	JQ286746	ALH24973	AFH35018	100%	97.2%	95.0%	95.0%	89.5%	95.2%	83.1%
QH-YS-199	FJ654226		ACV60385	99.8%	96.8%	94.8%	94.7%	89.3%	95.0%	82.9%
XZ-NQ-154	FJ654206		ACV60363	99.8%	96.8%	94.8%	94.7%	89.3%	95.0%	82.9%
XZ-NQ-155	FJ654207		ACV60364	99.8%	96.8%	94.8%	94.7%	89.3%	95.0%	82.9%
GS09_229	JQ325059	ALH24951	AFX66394	99.1%	94.4%	88.6%	88.9%	94.6%	82.8%	82.8%
GS09_281	JQ325061	ALH24952	AFX66396	99.1%	94.0%	88.6%	88.9%	94.6%	82.8%	82.8%
GS10_1	JQ325064	ALH24953	AFX66399	99.1%	94.0%	88.6%	88.9%	94.6%	82.8%	82.8%
QH09_164	JQ325077	ALH24962	AFX66412	99.1%	94.0%	88.6%	88.9%	94.6%	82.8%	82.8%
QH09_173	JQ325078	ALH24963	AFX66413	99.1%	94.0%	88.6%	88.9%	94.6%	82.8%	82.8%
QH09_201	JQ325080	ALH24964	AFX66415	99.1%	94.0%	88.6%	88.9%	94.6%	82.8%	82.8%
QH09_210	JQ325081	ALH24966	AFX66416	99.1%	94.0%	88.6%	88.9%	94.6%	82.8%	82.8%
SC09_87	JQ325092	ALH24994		99.1%	94.0%	88.6%	88.9%	94.6%	82.8%	82.8%
GS09_311	JQ325062	ALH25054	AFX66397	98.9%	94.2%	88.4%	88.7%	94.4%	82.6%	82.8%
XZ05_2	JQ325106	ALH25001	AFX66441	98.9%	95.0%	88.6%	90.1%	95.3%	83.5%	83.5%
XZ09_80	JQ325126	ALH25055	AFX66461	98.9%	96.8%	95.0%	93.7%	90.0%	95.3%	83.5%
XZ-LZ05-6	FJ654259		ACV60415	98.9%	96.8%	95.0%	93.7%	90.0%	95.3%	83.5%
YN09_61	JQ325140	ALH25047	AFX66475	98.9%	97.2%	95.0%	94.1%	90.2%	96.1%	84.0%
ID10_1	JQ325149	ALH24954	AFX66484	98.9%	97.2%	95.0%	94.1%	90.2%	96.1%	84.0%
SC-3	FJ654238		ACV60399	98.7%	96.8%	95.0%	93.7%	90.4%	95.5%	83.7%
XZ06_260	JQ325111	ALH25008	AFX66446	98.7%	96.8%	94.8%	93.5%	89.9%	95.2%	83.5%
XZ-SN-44	FJ654218		ACV60375	98.7%	96.8%	94.8%	93.5%	89.9%	95.2%	83.5%
YN09_51	JQ325139	ALH25045	AFX66474	97.6%	95.0%	87.1%	89.1%	94.0%	82.0%	82.0%
YN09_6	JQ325137	ALH25046	AFX66472	97.2%	95.1%	86.4%	88.4%	93.5%	81.2%	81.2%
YN09_22	JQ325138	ALH25043	AFX66473	97.0%	94.9%	86.2%	88.2%	93.3%	81.0%	81.0%
YN09_64	JQ325141	ALH25048	AFX66476	97.0%	94.5%	86.6%	88.6%	93.5%	81.6%	81.6%
XZ12_16	KM197540	ALH25057	AIV43040	95.7%	96.1%	99.6%	87.7%	92.2%	81.1%	81.1%
XZ05_8	JQ325108	ALH25005	AFX66443	96.1%	95.8%	98.6%	91.5%	87.9%	92.5%	81.1%
XZ-LZ07-H1	FJ654148		ACV60417	96.1%	95.8%	98.6%	91.5%	87.9%	92.5%	81.1%
XZ06-124	JQ286748	ALH25006	AFH35020	96.3%	95.8%	98.4%	91.8%	88.1%	92.7%	81.3%
XZ-LZ07-H2	FJ654149		ACV60418	96.3%	95.8%	98.4%	91.8%	88.1%	92.7%	81.3%

Note: The accession numbers in green refer to the MAT1-1-1 and MAT1-2-1 protein sequences recorded in the GenBank database but not in the AlphaFold database. Those in red were recorded in both the GenBank and AlphaFold databases. The percentages in blue indicate high homology (≥97%) to the reference sequences of GC-biased Genotypes #1 (AB067721), #2 (MG770309), #3 (HM595984), #7 (AJ488254), #8 (GU246286), #9 (GU246288), and #10 (GU246287) [7]

Table S5. Amino acids are scaled based on the general chemical characteristics of their side chains for the ProtScale analysis (https://web.expasy.org/protscale/) to predict the hydrophobicity and secondary structures (α-helices, β-sheets, β-turns, and coils) of proteins.

		Chemical-physical property	hydropathy index *	α-Helix	β-Sheet	β-Turn	Coil
Phenylalanine	Phe, F	Aromatic	2.800	1.195	1.393	0.624	0.797
Tryptophan	Trp, W	Aromatic	-0.900	1.090	1.306	0.546	0.941
Tyrosine	Tyr, Y	Aromatic	-1.300	0.787	1.266	0.795	1.109
Isoleucine	Ile, I	Aliphatic	4.500	1.003	1.799	0.240	0.886
Valine	Val, V	Aliphatic	4.200	0.990	1.965	0.387	0.772
Leucine	Leu, L	Aliphatic	3.800	1.236	1.261	0.670	0.810
Alanine	Ala, A	Aliphatic	1.800	1.489	0.709	0.788	0.824
Cysteine	Cys, C	with polar neutral side chains	2.500	0.966	1.191	0.965	0.953
Methionine	Met, M	with polar neutral side chains	1.900	1.363	1.210	0.436	0.810
Serine	Ser, S	with polar neutral side chains	-0.800	0.739	0.928	1.316	1.130
Threonine	Thr, T	with polar neutral side chains	-0.700	0.785	1.221	0.739	1.148
Asparagine	Asn, N	with polar neutral side chains	-3.500	0.772	0.604	1.572	1.167
Glutamine	Gln, Q	with polar neutral side chains	-3.500	1.164	0.840	0.997	0.947
Histidine	His, H	Basic	-3.200	1.003	0.863	0.970	1.068
Lysine	Lys, K	Basic	-3.900	1.172	0.721	1.302	0.897
Arginine	Arg, R	Basic	-4.500	1.224	0.920	0.912	0.893
Aspartic acid	Asp, D	Acidic	-3.500	0.924	0.541	1.197	1.197
Glutamic acid	Glu, E	Acidic	-3.500	1.504	0.567	1.149	0.761
Glycine	Gly, G	Unique amino acid	-0.400	0.510	0.657	1.860	1.251
Proline	Pro, P	Unique amino acid	-1.600	0.492	0.354	1.415	1.540

Note: An amino acid scale is defined at https://web.expasy.org/protscale/ by a numerical value assigned to each type of amino acid. The most frequently used scales are the hydrophobicity or hydrophilicity scales and the secondary structure conformational parameter scales, but many other scales exist, which are based on the different chemical and physical properties of the amino acids. The ExPASy ProtScale program provides 57 predefined scales on the basis of the literature [68]. *, Hydropathy index [67]; the larger the value is, the stronger the hydrophobicity; negative values indicate hydrophilicity.

Table S6. Summary of the results shown in Figures 1, 3–8, and S1 with the amino acid substitutions in the MATα_HMGbox domains of the 19 full-length MAT1-1-1 proteins of the wild-type *C. sinensis* isolates based on AlphaFold UniProt codes and GenBank accession numbers.

***C. sinensis* isolate**	**GenBank accession #**	**AlphaFold UniProt code**	**The Bayesian cluster based on full length sequence***	**The Bayesian cluster based on the sequence of MATα_HMGbox domain**	**Amino acid substitution in the MATα_HMGbox domain (*vs.* AGW27560)**
SC09_65	ALH24992	A0A0N9R5B3	A2	a2	A-to-D
GS09_143	ALH24948	A0A0N9QUF3	A3	e2	QI-to-SF
YN09_22<br>YN09_51<br>YN09_6<br>YN09_64	ALH25043<br>ALH25045<br>ALH25046<br>ALH25048	A0A0N9QMS9	B	b	R-to-I
GS09_311<br>GS09_229<br>GS09_281<br>GS10_1<br>QH09_164<br>QH09_173<br>QH09_201<br>QH09_210<br>SC09_87	ALH25054<br>ALH24951<br>ALH24952<br>ALH24953<br>ALH24962<br>ALH24963<br>ALH24964<br>ALH24966<br>ALH24994	A0A0N7G845	C	e1	I-to-L
XZ07_H2<br>XZ12_16	ALH24999<br>ALH25057	A0A0N9QMT4	D2	d	A-to-V and A-to-T
XZ05_2	ALH25001	A0A0N9R4Q4	E4	d	A-to-V and A-to-T
XZ05_6	ALH25003	A0A0N7G850	E3	a2	S-to-G

Note: *, The Bayesian clustering results were reported previously by Li *et al*. [50].

Table S7. Summary of the Bayesian clustering results shown in Figures 1, 9, S1, and S3 with the amino acid substitutions in the MATα_HMGbox domains of the MAT1-1-1 proteins encoded by the genome assemblies of *H. sinensis* strains and the metatranscriptome assemblies of natural *C. sinensis* based on the GenBank accession numbers.

***H. sinensis* strain**	**GenBank accession #**	**The MATα_HMGbox domain: Nucleotide sequence arranges (deleted the intron sequence)**	**The MATα_HMGbox domain: Bayesian cluster**	**The MATα_HMGbox domain: Amino acid substitution or deletion (*vs.* AGW27560)**
IOZ07	JAAVMX010000001	6,699,061→6,699,153 &<br>6,699,203→6,699.637	c	Y-to-M
1229	LKHE01001116	4183←4620 & 4667←4759	c	Y-to-M
Co18	ANOV01017390	794←1129 & 1280←1396	a1	(100% identical)
†	OSIN7648	151→675	a1	(100% identical)
†	GAGW01008880	714←1127	a1	46 aa deletions at the N-terminus of the domain

Note: †, MAT1-1-1 transcripts GAGW01008880 and OSIN7648 were derived from *C. sinensis* insect–fungi complexes. The arrows "→" and "←" indicate sequences in the sense and antisense strands of the genomes, respectively; "&" refers to the removed intron portion.

Table S8. Summary of the results shown in Figures 2, 10–13, and S2 with the amino acid substitutions in the HMG-box_ROX1-like domains of the 25 full-length MAT1-2-1 proteins of wild-type *C. sinensis* isolates based on the AlphaFold UniProt codes and GenBank accession numbers.

***C. sinensis* isolate**	**GenBank accession #**	**AlphaFold UniProt code**	**The Bayesian Cluster based on**		**Amino acid substitution in the HMG-box_ROX1-like domain (*vs*. AEH27625)**
			**full length sequence***	**HMG-box_ROX1-like domain sequence**	
XZ12_16	AIV43040	A0A0A0RCF5	II-1	b2β	V-to-H, M-to-I, and Q-to-R
XZ-LZ07-H1<br>XZ-LZ07-H2<br>XZ06-124<br>XZ05_8	ACV60417<br>ACV60418<br>AFH35020<br>AFX66443	D7F2J7	II-2	b2*α*	V-to-H and M-to-I
XZ-SN-44<br>XZ-LZ05-6<br>XZ05_2<br>XZ06_260<br>XZ09_80	ACV60375<br>ACV60415<br>AFX66441<br>AFX66446<br>AFX66461	D7F2F5	III	b2*α*	V-to-H and M-to-I
XZ-NQ-154<br>XZ-NQ-155<br>GS09_111<br>QH09-93<br>CS560-961	ACV60363<br>ACV60364<br>AFX66388<br>AFH35018<br>AGW27542	D7F2E3	V-1	b1*α*	V-to-H
QH-YS-199	ACV60385	D7F2G5	V-2	b1*α*	V-to-H
QH09_11	AFX66401	V9LW71	V-2	b1*α*	V-to-H
YN09_6<br>YN09_22<br>YN09_51	AFX66472<br>AFX66473<br>AFX66474	V9LVS8	IV-2	b1*α*	V-to-H
YN09_61	AFX66475	V9LVU8	V-2	b1*α*	V-to-H
YN09_64	AFX66476	V9LWC9	IV-1	b1*α*	V-to-H
ID10_1	AFX66484	V9LWG5	V-2	b1*α*	V-to-H
CS6-251	AGW27537	U3N6V5	V-2	b1*α*	V-to-H
SC-3	ACV60399	D7F2H9	V-2	b1β	V-to-H and Q-to-R

Note: *, The Bayesian clustering results were reported by Li *et al*. [50].

Table S9. Summary of the results shown in Figures 2, 14–16, and S2 with the amino acid substitutions and deletions in the HMG-box_ROX1-like domains of the MAT1-2-1 proteins encoded by the genome and transcriptome assemblies of *H. sinensis* strains and the metatranscriptome assembly of natural *C. sinensis* insect–fungal complexes found based on the GenBank accession numbers.

***H. sinensis* strain**	**GenBank accession #**	**The HMG-box_ROX1-like domain**		
		**Nucleotide sequence arranges (deleted intron region)**	**Bayesian cluster**	**Amino acid substitution (*vs.* AEH27625)**
Co18	ANOV01000063	9759→9851 & 9907→10,026	a2	S-to-A
1229	LKHE01001605	14,016←14,135 & 14,191←14,283	b3	Y-to-H and S-to-A
ZJB12195	LWBQ01000021	239,029←239,148 & 239,204←239,269	b3	Y-to-H and S-to-A
CC1406-20395	NGJJ01000619	23,186←23,305 & 23,361←23,453	b3	Y-to-H and S-to-A
L0106	GCQL01020543	553←765	b1α	Y-to-H
†	OSIN7649	379→591	b1α	(100% identical)

Note: †, The MAT1-2-1 transcript OSIN7649 was derived from the mature *C. sinensis* insect–fungi complex. The arrows "→" and "←" indicate sequences in the sense and antisense strands of the genomes, respectively; "&" refers to the removed intron portion.

[REFERENCES]

1. Zhu, J.-S.; Halpern, G.M.; Jones, K. The scientific rediscovery of a precious ancient Chinese herbal regimen: *Cordyceps sinensis*: Part I. *J. Altern. Complem. Med.* **1998**, *4*, 289–303. https://doi.org/10.1089/acm.1998.4.3-289.
2. Zhu, J.-S.; Halpern, G.M.; Jones, K. The scientific rediscovery of an ancient Chinese herbal medicine: *Cordyceps sinensis*: Part II. *J. Altern. Complem. Med.* **1998**, *4*, 429–457. https://doi.org/10.1089/acm.1998.4.429.
3. Zhu, J.-S.; Li, C.-L.; Tan, N.-Z.; Berger, J.L.; Prolla, T.A. Combined use of whole-gene expression profiling technology and mouse lifespan test in anti-aging herbal product study. In Proceedings of the 2011 New TCM Products Innovation and Industrial Development Summit, Hangzhou, China, 27 November 2011; pp. 443–448. Available online: https://xueshu.baidu.com/usercenter/paper/show?paperid=08341c17fa58c8f85584b92572b90f75&site=xueshu_se (accessed on 30 January 2025).
4. Ren, Y.; Wan, D.-G.; Lu, X.-M.; Guo, J.-L. The study of scientific name discussion for TCM Cordyceps. *LisShenzhen Med. Mater. Medica Res.* **2013**, *24*, 2211–2212.
5. Zhang, Y.-J.; Zhang, S.; Li, Y.-L.; Ma, S.-L.; Wang, C.-S.; Xiang, M.-C.; Liu, X.; An, Z.-Q.; Xu, J.-P.; Liu, X.-Z. Phylogeography and evolution of a fungal–insect association on the Tibetan Plateau. *Mol. Ecol.* **2014**, *23*, 5337–5355. https://doi.org/10.1111/mec.12940.
6. Lu, H.-L.; St. Leger, R.J. Chapter Seven—Insect Immunity to Entomopathogenic Fungi. In *Advances in Genetics*; Lovett, B., St. Leger, R.J., Eds.; Academic Press: Cambridge, MA, USA, 2016; Volume 94, pp. 251–285.
7. Li, Y.-L.; Li, X.-Z.; Yao, Y.-S.; Xie, W.-D.; Zhu, J.-S. Molecular identification of *Ophiocordyceps sinensis* genotypes and the indiscriminate use of the Latin name for the multiple genotypes and the natural insect–fungi complex. *Am. J. BioMed. Sci.* **2022**, *14*, 115–135. https://doi.org/10.5099/aj220300115.
8. Li, M.-M.; Zhang, J.-H.; Qin, Q.-L.; Zhang, H.; Li, X.; Wang, H.-T.; Meng, Q. Transcriptome and Metabolome Analyses of Thitarodes xiaojinensis in Response to *Ophiocordyceps sinensis* Infection. *Microorganisms* **2023**, *11*, 2361. https://doi.org/10.3390/microorganisms11092361.

9. Li, Y.-L.; Gao, L.; Yao, Y.-S.; Wu, Z.-M.; Lou, Z.-Q.; Xie, W.-D.; Wu, J.-Y.; Zhu, J.-S. Altered GC- and AT-biased genotypes of *Ophiocordyceps sinensis* in the stromal fertile portions and ascospores of natural *Cordyceps sinensis*. *PLoS ONE.* **2023**, *18*, e0286865. https://doi.org/10.1371/journal.pone.0286865.
10. Li, Y.-L.; Li, X.-Z.; Yao, Y.-S.; Wu, Z.-M.; Gao, L.; Tan, N.-Z.; Lou, Z.-Q.; Xie, W.-D.; Wu, J.-Y.; Zhu, J.-S. Differential cooccurrence of multiple genotypes of *Ophiocordyceps sinensis* in the stromata, stromal fertile portion (ascocarps) and ascospores of natural *Cordyceps sinensis*. *PLoS ONE* **2023**, *18*, e0270776. https://doi.org/10.1371/journal.pone.0270776.
11. Li, C.-L. A study of *Tolypocladium sinense* C.L. Li. sp. nov. and cyclosporin production. *Acta Mycol. Sin.* **1988**, *7*, 93−98.
12. Dai, R.-Q.; Lan, J.-L.; Chen, W.-H.; Li, X.-M.; Chen, Q.-T.; Shen, C.-Y. Discovery of a new fungus *Paecilomyces hepiali* Chen & Dai. *Acta Agricult. Univ. Pekin.* **1989**, *15*, 221−224.
13. Dai, R.-Q.; Li, X.-M.; Shao, A.-J.; Lin, S.-F.; Lan, J.-L.; Chen, W.-H.; Shen, C.-Y. Nomenclatural validation of *Paecilomyces hepiali*. *Mycosystema* **2008**, *27*, 641–644.
14. Kinjo, N.; Zang, M. Morphological and phylogenetic studies on *Cordyceps sinensis* distributed in southwestern China. *Mycoscience* **2001**, *42*, 567–574. https://doi.org/10.1007/BF02460956.
15. Jiang, Y.; Yao, Y.-J. A review for the debating studies on the anamorph of *Cordyceps sinensis*. *Mycosistema* **2003**, *22*, 161–176.
16. Stensrud, Ø.; Hywel-Jones, N.L.; Schumacher, T. Towards a phylogenetic classification of Cordyceps: ITS nrDNA sequence data confirm divergent lineages and paraphyly. *Mycol. Res.* **2005**, *109*, 41–56. https://doi.org/10.1017/s095375620400139x.
17. Stensrud, Ø.; Schumacher, T.; Shalchian-Tabrizi, K.; Svegardenib, I.B.; Kauserud, H. Accelerated nrDNA evolution and profound AT bias in the medicinal fungus *Cordyceps sinensis*. *Mycol. Res.* **2007**, *111*, 409–415. https://doi.org/10.1016/j.mycres.2007.01.015.
18. Leung, P.-H.; Zhang, Q.-X.; Wu, J.-Y. Mycelium cultivation, chemical composition and antitumour activity of a *Tolypocladium* sp. fungus isolated from wild *Cordyceps sinensis*. *J. Appl. Microbiol.* **2006**, *101*, 275–283. https://doi.org/10.1111/j.1365-2672.2006.02930.x.
19. Yang, J.-L.; Xiao, W.; He, H.-X.; Zhu, H.-X.; Wang, S.-F.; Cheng, K.-D.; Zhu, P. Molecular phylogenetic analysis of *Paecilomyces hepiali* and *Cordyceps sinensis*. *Acta Pharmaceut. Sinica.* **2008**, *43*, 421–426.
20. Yang, J.-Y.; Tong, X.-X.; He, C.-Y.; Bai, J.; Wang, F.; Guo, J.-L. Comparison of endogenetic microbial community diversity between wild *Cordyceps sinensis*, artificial *C. sinensis* and habitat soil. *Chin. J. Chin. Mater. Medica* **2021**, *46*, 3106–3115.
21. Zhang, Y.-J.; Sun, B.-D.; Zhang, S.; Wàngmǔ Liu, X.-Z.; Gong, W.-F. Mycobiotal investigation of natural *Ophiocordyceps sinensis* based on culture-dependent investigation. *Mycosistema* **2010**, *29*, 518–527.
22. Zhang, S.-W.; Cen, K.; Liu, Y.; Zhou, X.-W.; Wang, C.-S. Metatranscriptomics analysis of the fruiting caterpillar fungus collected from the Qinghai-Tibetan plateau. *Sci. Sinica Vitae* **2018**, *48*, 562–570.
23. Li, Y.; Jiao, L.; Yao, Y.-J. Non-concerted ITS evolution in fungi, as revealed from the important medicinal fungus *Ophiocordyceps sinensis*. *Mol. Phylogenet. Evol.* **2013**, *68*, 373–379. https://doi.org/10.1016/j.ympev.2013.04.010.
24. Li, Y.; Jiang, L.; Wang, K.; Wu, H.-J.; Yang, R.-H.; Yan, Y.-J.; Bushley, K.E.; Hawksworth, D.L.; Wu, Z.-J.; Yao, Y.-J. RIP mutated ITS genes in populations of *Ophiocordyceps sinensis* and their implications for molecular systematics. *IMA Fungus* **2020**, *11*, 18.
25. Meng, Q.; Yu, H.-Y.; Zhang, H.; Zhu, W.; Wang, M.-L.; Zhang, J.-H.; Zhou, G.-L.; Li, X.; Qin, Q.-L.; Hu, S.-N.; et al. Transcriptomic insight into the immune defenses in the ghost moth, Hepialus xiaojinensis, during an *Ophiocordyceps sinensis* fungal infection. Insect Biochem. *Mol. Biol.* **2015**, *64*, 1–15. https://doi.org/10.1016/j.ibmb.2015.06.014.
26. Xia, F.; Liu, Y.; Shen, G.-L.; Guo, L.-X.; Zhou, X.-W. Investigation and analysis of microbiological communities in natural *Ophiocordyceps sinensis*. *Can. J. Microbiol.* **2015**, *61*, 104–111. https://doi.org/10.1139/cjm-2014-0610.
27. Guo, M.-Y.; Liu, Y.; Gao, Y.-H.; Jin, T.; Zhang, H.-B.; Zhou, X.-W. Identification and bioactive potential of endogenetic fungi isolated from medicinal caterpillar fungus *Ophiocordyceps sinensis* from Tibetan Plateau. *Int. J. Agric. Biol.* **2017**, *19*, 307–313. https://doi.org/10.17957/IJAB/15.0281.
28. Zhong, X.; Gu, L.; Wang, H.-Z.; Lian, D.-H.; Zheng, Y.-M.; Zhou, S.; Zhou, W.; Gu, J.; Zhang, G.; Liu, X. Profile of *Ophiocordyceps sinensis* transcriptome and differentially expressed genes in three different mycelia, sclerotium and fruiting body developmental stages. *Fungal Biol.* **2018**, *122*, 943–951. https://doi.org/10.1016/j.funbio.2018.05.011.
29. Kang, Q.; Zhang, J.; Chen, F.; Dong, C.; Qin, Q.; Li, X.; Wang, H.; Zhang, H.; Meng, Q. Unveiling mycoviral diversity in *Ophiocordyceps sinensis* through transcriptome analyses. *Front. Microbiol.* **2024**, *15*, 1493365. https://doi.org/10.3389/fmicb.2024.1493365.
30. China Ministry of Agriculture and Rural Affairs. Announcement (No. 15 of 2021) of National Forestry and Grassland Administration: List of National Key Protected Wild Plants. 7 September 2021. Available online: https://m.163.com/dy/article/HHCVOJPU055360T7.html (accessed on 3 May 2025).
31. Wei, X.-L.; Yin, X.-C.; Guo, Y.-L.; Shen, N.-Y.; Wei, J.-C. Analyses of molecular systematics on *Cordyceps sinensis* and its related taxa. *Mycosystema* **2006**, *25*, 192–202.

32. Wei, J.-C.; Wei, X.-L.; Zheng, W.-F.; Guo, W.; Liu, R.-D. Species identification and component detection of *Ophiocordyceps sinensis* cultivated by modern industry. *Mycosystema* **2016**, *35*, 404–410.
33. Sung, G.-H.; Hywel-Jones, N.L.; Sung, J.-M.; Luangsa-ard, J.J.; Shrestha, B.; Spatafora, J.W. Phylogenetic classification of *Cordyceps* and the clavicipitaceous fungi. *Stud. Mycol.* **2007**, *57*, 5–59. https://doi.org/10.3114/sim.2007.57.01.
34. Zhang, Y.-J.; Li, E.-W.; Wang, C.-S.; Li, Y.-L.; Liu, X.-Z. *Ophiocordyceps sinensis*, the flagship fungus of China: Terminology, life strategy and ecology. *Mycology* **2012**, *3*, 2–10. https://doi.org/10.1080/21501203.2011.654354.
35. Wang, Y.; Stata, M.; Wang, W.; Stajich, J.E.; White, M.M.; Moncalvo, J.M. Comparative genomics reveals the core gene toolbox for the fungus-insect symbiosis. *mBio* **2018**, *9*, e00636-e18. https://doi.org/10.1128/mBio.00636-18.
36. Zhang, S.; Zhang, Y.-J.; Shrestha, B.; Xu, J.-P.; Wang, C.-S.; Liu, X.-Z. *Ophiocordyceps sinensis* and *Cordyceps militaris*: Research advances, issues and perspectives. *Mycosystema* **2013**, *32*, 577–597.
37. Hawksworth, D.L.; Crous, P.W.; Redhead, S.A.; Reynolds, D.R.; Samson, R.A.; Seifert, K.A.; Taylor, J.W.; Wingfield, M.J.; Abaci, Ö.; Aime, C.; et al. The Amsterdam declaration on fungal nomenclature. *IMA Fungus* **2011**, *2*, 105–112. https://doi.org/10.5598/imafungus.2011.02.01.14.
38. Turgeon, B.G.; Yoder, O.C. Proposed nomenclature for mating type genes of filamentous ascomycetes. *Fungal Genet. Biol.* **2000**, *31*, 1–5. https://doi.org/10.1006/fgbi.2000.1227.
39. Debuchy, R.; Turgeo, B.G. Mating-Type Structure, Evolution, and Function in Euascomycetes. In *Growth, Differentiation and Sexuality*; Kües, U., Fischer, R., Eds.; Springer: Berlin/Heidelberg, Germany, 2006; pp. 293–323.
40. Jones, S.K.; Bennett, R.J. Fungal mating pheromones: Choreographing the dating game. *Fungal Genet. Biol.* **2011**, *48*, 668–676. https://doi.org/10.1016/j.fgb.2011.04.001.
41. Zheng, P.; Wang, C.-S. Sexuality Control and Sex Evolution in Fungi. *Sci. Sin. Vitae* **2013**, *43*, 1090–1097.
42. Wilson, A.M.; Wilken, P.M.; van der Nest, M.A.; Steenkamp, E.T.; Wingfield, M.J.; Wingfield, B.D. Homothallism: An umbrella term for describing diverse sexual behaviours. *IMA Fungus* **2015**, *6*, 207–214. https://doi.org/10.5598/imafungus.2015.06.01.13.
43. Sun, S.; Coelho, M.A.; David-Palma, M.; Priest, S.J.; Heitman, J. The evolution of sexual reproduction and the mating-type locus: Links to pathogenesis of *Cryptococcus* human pathogenic fungi. *Annu. Rev. Genet.* **2019**, *53*, 417–444. https://doi.org/10.1146/annurev-genet-120116-024755.
44. Ramšak, B.; Markau, J.; Pazen, T.; Dahlmann, T.A.; Krappmann, S.; Kűck, U. The master regulator MAT1-1-1 of fungal mating binds to its targets via a conserved motif in the human pathogen *Aspergillus fumigatus*. *G3 Genes Genom. Genet.* **2020**, *11*, jkaa012. https://doi.org/10.1093/g3journal/jkaa012.
45. Ramšak, B.; Kűck, U.; Hofmann, E. The mating type transcription factor MAT1-1-1 from the fungal human pathogen *Aspergillus fumigatus*: Synthesis, purification, and crystallization of the DNA binding domain. *bioRxiv* **2021**. https://doi.org/10.1101/2021.12.13.472399.
46. Ramšak, B.; Kück, U. The *Penicillium chrysogenum* tom1 gene a major target of transcription factor MAT1-1-1 encodes a nuclear protein involved in sporulation. *Front. Fungal Bio.* **2022**, *3*, 937023. https://doi.org/10.3389/ffunb.2022.937023.
47. Metin, B.; Findley, K.; Heitman, J. The mating type locus (MAT) and sexual reproduction of *Cryptococcus heveanensis*: Insights into the evolution of sex and sex-determining chromosomal regions in fungi. *PLoS Genet.* **2010**, *6*, e1000961. https://doi.org/10.1371/journal.pgen.1000961.
48. Bushley, K.E.; Li, Y.; Wang, W.-J.; Wang, X.-L.; Jiao, L.; Spatafora, J.W.; Yao, Y.-J. Isolation of the MAT1-1 mating type idiomorph and evidence for selfing in the Chinese medicinal fungus *Ophiocordyceps sinensis*. *Fungal Biol.* **2013**, *117*, 599–610. https://doi.org/10.1016/j.funbio.2013.06.001.
49. Hu, X.; Zhang, Y.-J.; Xiao, G.-H.; Zheng, P.; Xia, Y.-L.; Zhang, X.-Y.; St Leger, R.J.; Liu, X.-Z.; Wang, C.-S. Genome survey uncovers the secrets of sex and lifestyle in caterpillar fungus. *Chin. Sci. Bull.* **2013**, *58*, 2846–2854. https://doi.org/10.1007/s11434-013-5929-5.
50. Li, X.-Z.; Li, Y.-L.; Zhu, J.-S. Three-dimensional structural heteromorphs of mating-type proteins in *Hirsutella sinensis* and the natural *Cordyceps sinensis* insect–fungal complex. *J. Fungi.* **2025**, *11*, 244. https://doi.org/10.3390/jof11040244.
51. Zhou, X.W.; Li, L.J.; Tian, E.W. Advances in research of the artificial cultivation of *Ophiocordyceps sinensis* in China. *Crit. Rev. Biotechnol.* **2013**, *34*, 233–243. https://doi.org/10.3109/07388551.2013.791245.
52. Zhang, S.; Zhang, Y.-J. Molecular evolution of three protein-coding genes in the Chinese caterpillar fungus *Ophiocordyceps sinensis*. *Microbiol. China.* **2015**, *42*, 1549–1560.
53. Li, X.; Wang, F.; Liu, Q.; Li, Q.-P.; Qian, Z.-M.; Zhang, X.-L.; Li, K.; Li, W.-J.; Dong, C.-H. Developmental transcriptomics of Chinese cordyceps reveals gene regulatory network and expression profiles of sexual development-related genes. *BMC Genom.* **2019**, *20*, 337. https://doi.org/10.1186/s12864-019-5708-z.

54. Li, X.-Z.; Li, Y.-L.; Zhu, J.-S. Differential transcription of mating-type genes during sexual reproduction of natural *Cordyceps sinensis*. *Chin. J. Chin. Mater. Medica* **2023**, *48*, 2829–2840. https://doi.org/10.19540/j.cnki.cjcmm.20230213.102.
55. Li, X.-Z.; Xiao, M.-J.; Li, Y.-L.; Gao, L.; Zhu, J.-S. Mutations and differential transcription of mating-type and pheromone receptor genes in *Hirsutella sinensis* and the natural *Cordyceps sinensis* insect–fungi complex. *Biology* **2024**, *13*, 632. https://doi.org/10.3390/biology13080632.
56. Zhang, Y.-J.; Xu, L.-L.; Zhang, S.; Liu, X.-Z.; An, Z.-Q.; Wàngmǔ Guo, Y.-L. Genetic diversity of *Ophiocordyceps sinensis*, a medicinal fungus endemic to the Tibetan Plateau: Implications for its evolution and conservation. *BMC Evol. Biol.* **2009**, *9*, 290. https://doi.org/10.1186/1471-2148-9-290.
57. Zhang, S.; Zhang, Y.-J.; Liu, X.-Z.; Wen, H.-A.; Wang, M.; Liu, D.-S. Cloning and analysis of the MAT1-2-1 gene from the traditional Chinese medicinal fungus *Ophiocordyceps sinensis*. *Fungal Biol.* **2011**, *115*, 708–714.
58. Liu, Z.-Q.; Lin, S.; Baker, P.J.; Wu, L.-F.; Wang, X.-R.; Wu, H.; Xu, F.; Wang, H.-Y.; Brathwaite, M.E.; Zheng, Y.-G. Transcriptome sequencing and analysis of the entomopathogenic fungus *Hirsutella sinensis* isolated from *Ophiocordyceps sinensis*. *BMC Genom.* **2015**, *16*, 106–123. https://doi.org/10.1186/s12864-015-1269-y.
59. Liu, J.; Guo, L.-N.; Li, Z.-W.; Zhou, Z.; Li, Z.; Li, Q.; Bo, X.-C.; Wang, S.-Q.; Wang, J.-L.; Ma, S.-C.; et al. Genomic analyses reveal evolutionary and geologic context for the plateau fungus *Ophiocordyceps sinensis*. *Clin. Med.* **2020**, *15*, 107–119. https://doi.org/10.1186/s13020-020-00365-3.
60. Li, Y.; Hsiang, T.; Yang, R.-H.; Hu, X.-D.; Wang, K.; Wang, W.-J.; Wang, X.-L.; Jiao, L.; Yao, Y.-J. Comparison of different sequencing and assembly strategies for a repeat-rich fungal genome, *Ophiocordyceps sinensis*. *J. Microbiol. Methods* **2016**, *128*, 1–6. https://doi.org/10.1016/j.mimet.2016.06.025.
61. Jin, L.-.Q.; Xu, Z.-.W.; Zhang, B.; Yi, M.; Weng, C.-.Y.; Lin, S.; Wu, H.; Qin, X.-T.; Xu, F.; Teng, Y.; et al. Genome sequencing and analysis of fungus *Hirsutella sinensis* isolated from *Ophiocordyceps sinensis*. *AMB Expr.* **2020**, *10*, 105. https://doi.org/10.1186/s13568-020-01039-x.
62. Shu, R.-H.; Zhang, J.-H.; Meng, Q.; Zhang, H.; Zhou, G.-L.; Li, M.-M.; Wu, P.-P.; Zhao, Y.-N.; Chen, C.; Qin, Q.-L. A new high-quality draft genome assembly of the Chinese cordyceps *Ophiocordyceps sinensis*. *Genome Biol. Evol.* **2020**, *12*, 1074–1079. https://doi.org/10.1093/gbe/evaa112.
63. Xiang, L.; Li, Y.; Zhu, Y.; Luo, H.; Li, C.; Xu, X.; Sun, C.; Song, J.-Y.; Shi, L.-H.; He, L.; et al. Transcriptome analysis of the *Ophiocordyceps sinensis* fruiting body reveals putative genes involved in fruiting body development and cordycepin biosynthesis. *Genomics* **2014**, *103*, 154–159. https://doi.org/10.1016/j.ygeno.2014.01.002.
64. Xia, E.-H.; Yang, D.-R.; Jiang, J.-J.; Zhang, Q.-J.; Liu, Y.; Liu, Y.-L.; Zhang, Y.; Zhang, H.-B.; Shi, C.; Tong, Y.; et al. The caterpillar fungus, *Ophiocordyceps sinensis*, genome provides insights into highland adaptation of fungal pathogenicity. *Sci. Rep.* **2017**, *7*, 1806. https://doi.org/10.1038/s41598-017-01869-z.
65. Huelsenbeck, J.P.; Ronquist, F. MRBAYES, Bayesian inference of phylogeny. *Bioinformatics* **2001**, *17*, 754–755. https://doi.org/10.1093/bioinformatics/17.8.754.
66. Ronquist, F.; Teslenko, M.; van der Mark, P.; Ayres, D.L.; Darling, A.; Höhna, S.; Larget, B.; Liu, L.; Suchard, M.A.; Huelsenbeck, J.P. MrBayes 3.2, Efficient Bayesian Phylogenetic Inference and Model Choice Across a Large Model Space. *Syst. Biol.* **2012**, *61*, 539–542. https://doi.org/10.1093/sysbio/sys029.
67. Kyte, J.; Doolittle, R.F. A simple method for displaying the hydropathic character of a protein. *J. Mol. Biol.* **1982**, *157*, 105–132. https://doi.org/10.1016/0022-2836(82)90515-0.
68. Deleage, G.; Roux, B. An algorithm for protein secondary structure prediction based on class prediction. *Protein Eng. Des. Sel.* **1987**, *1*, 289–294. https://doi.org/10.1093/protein/1.4.289.
69. Gasteiger, E.; Hoogland, C.; Gattiker, A.; Duvaud, S.; Wilkins, M.R.; Appel, R.D.; Bairoch, A. Protein Identification and Analysis Tools on the ExPASy Server, Chapter 52. In *The Proteomics Protocols Handbook*; Walker, J.M., Ed.; Humana Press: Totowa, NJ, USA, 2005; pp. 571–607.
70. Peters, C.; Elofsson, A. Why is the biological hydrophobicity scale more accurate than earlier experimental hydrophobicity scales? *Proteins* **2014**, *82*, 2190–2198. https://doi.org/10.1002/prot.24582.
71. Simm, S.; Einloft, J.; Mirus, O.; Schleiff, E. 50 years of amino acid hydrophobicity scales, revisiting the capacity for peptide classification. *Biol. Res.* **2016**, *49*, 31. https://doi.org/10.1186/s40659-016-0092-5.
72. Tunyasuvunakool, K.; Adler, J.; Wu, Z.; Green, T.; Zielinski, M.; Žídek, A.; Bridgland, A.; Cowie, A.; Meyer, C.; Laydon, A.; et al. Highly accurate protein structure prediction for the human proteome. *Nature* **2021**, *596*, 590–596. https://doi.org/10.1038/s41586-021-03828-1.

73. Abramson, J.; Adler, J.; Dunger, J.; Evans, R.; Green, T.; Pritzel, A.; Ronneberger, O.; Willmore, L.; Ballard, A.J.; Bambrick, J.; et al. Accurate structure prediction of biomolecular interactions with AlphaFold 3. *Nature* **2024**, *630*, 493–500. https://doi.org/10.1038/s41586-024-07487-w.
74. Jumper, J.; Evans, R.; Pritzel, A.; Green, T.; Figurnov, M.; Ronneberger, O.; Tunyasuvunakool, K.; Bates, R.; Žídek, A.; Potapenko, A.; et al. Highly accurate protein structure prediction with AlphaFold. *Nature* **2021**, *596*, 583–589. https://doi.org/10.1038/s41586-021-03819-2.
75. David, A.; Islam, S.; Tankhilevich, E.; Sternberg, M.J.E. The AlphaFold Database of Protein Structures, A Biologist's Guide. *J. Mol. Biol.* **2022**, *434*, 167336. https://doi.org/10.1016/j.jmb.2021.167336.
76. Monzon, V.; Haft, D.H.; Bateman, A. Folding the unfoldable, using AlphaFold to explore spurious proteins. *Bioinform. Adv.* **2022**, *1*, vbab043. https://doi.org/10.1093/bioadv/vbab043.
77. Rettie, S.A.; Campbell, K.V.; Bera, A.K.; Kang, A.; Kozlov, S.; De La Cruz, J.; Adebomi, V.; Zhou, G.; DiMaio, F.; Ovchinnikov, S.; et al. Cyclic peptide structure prediction and design using AlphaFold. *bioRxiv* **2023**, 26:2023.02.25.529956. https://doi.org/10.1101/2023.02.25.529956.
78. Xu, T.; Xu, Q.; Li, J.-Y. Toward the appropriate interpretation of Alphafold2. *Front. Artif. Intell.* **2023**, *6*, 1149748. https://doi.org/10.3389/frai.2023.1149748.
79. Varadi, M.; Bertoni, D.; Magana, P.; Paramval, U.; Pidruchna, I.; Radhakrishnan, M.; Tsenkov, M.; Nair, S.; Mirdita, M.; Yeo, J.; et al. AlphaFold Protein Structure Database in 2024, providing structure coverage for over 214 million protein sequences. *Nucleic Acids Res.* **2024**, *52*, D368–D375. https://doi.org/10.1093/nar/gkad1011.
80. Wroblewski, K.; Kmiecik, S. Integrating AlphaFold pLDDT Scores into CABS-flex for enhanced protein flexibility simulations. *Comput. Struct. Biotechnol. J.* **2024**, *30*, 4350–4356. https://doi.org/10.1016/j.csbj.2024.11.047.
81. Ellison, T.J.; Ellison, C.K. Improved DNA binding to a type IV minor pilin increases natural transformation. *Nucleic Acids Res*. **2025**, *53*, gkaf467. https://doi.org/10.1093/nar/gkaf467.
82. Yang, X.; Zhu, H.-Q.; Shi, L.-X.; Song, T.-G.; Gong, W.-B.; He, S.-M.; Shan, S.; Xu, C.-F.; Zhou, Z. AlphaFold-guided structural analyses of nucleosome binding proteins. *Nucleic Acids Res.* **2025**, *53*, gkaf735. https://doi.org/10.1093/nar/gkaf735.
83. Mariani, V.; Biasini, M.; Barbato, A.; Schwede, T. lDDT, a local superposition-free score for comparing protein structures and models using distance difference tests. *Bioinformatics* **2013**, *29*, 2722–2728. https://doi.org/10.1093/bioinformatics/btt473.
84. Baxevanis, A.D.; Bryant, S.H.; Landsman, D. Homology model building of the HMG-1 box structural domain. *Nucleic Acids Res.* **1995**, *23*, 1019–1029. https://doi.org/10.1093/nar/23.6.1019.
85. Thapar, R. Structure-specific nucleic acid recognition by L-motifs and their diverse roles in expression and regulation of the genome. *Biochim. Biophys. Acta* **2015**, *1849*, 677–687. https://doi.org/10.1016/j.bbagrm.2015.02.006.
86. Li, Y.-L.; Yao, Y.-S.; Zhang, Z.-H.; Xu, H.-F.; Liu, X.; Ma, S.-L.; Wu, Z.-M.; Zhu, J.-S. Synergy of fungal complexes isolated from the intestines of *Hepialus lagii* larvae in increasing infection potency. *J. Fungal Res.* **2016**, *14*, 96–112.
87. Kück, U.; Bennett, R.J.; Wang, L.; Dyer, P.S. Editorial, Sexual and Parasexual Reproduction of Human Fungal Pathogens. *Front. Cell Infect. Microbiol.* **2022**, *12*, 934267. https://doi.org/10.3389/fcimb.2022.934267.
88. Bennett, R.J.; Johnson, A.D. Completion of a parasexual cycle in *Candida albicans* by induced chromosome loss in tetraploid strains. *EMBO J.* **2003**, *22*, 2505–2515. https://doi.org/10.1093/emboj/cdg235.
89. Sherwood, R.K.; Bennett, R.J. Fungal meiosis and parasexual reproduction--lessons from pathogenic yeast. *Curr. Opin. Microbiol.* **2009**, *12*, 599–607. https://doi.org/10.1016/j.mib.2009.09.005.
90. Seervai, R.N.H.; Jones, S.K.; Hirakawa, M.P.; Porman, A.M.; Bennett, R.J. Parasexuality and ploidy change in *Candida tropicalis*. *Eukaryot. Cell.* **2013**, *12*, 1629–1640. https://doi.org/10.1128/EC.00128-13.
91. Nakamura, N.; Tanaka, C.; Takeuchi-Kaneko, Y. Transmission of antibiotic-resistance markers by hyphal fusion suggests partial presence of parasexuality in the root endophytic fungus Glutinomyces brunneus. *Mycol. Progress.* **2019**, *18*, 453–462. https://doi.org/10.1007/s11557-018-1455-9.
92. Samarasinghe, H.; You, M.; Jenkinson, T.S.; Xu, J.-P.; James, T.Y. Hybridization Facilitates Adaptive Evolution in Two Major Fungal Pathogens. *Genes* **2020**, *11*, 101. https://doi.org/10.3390/genes11010101.
93. Steensels, J.; Gallone, B.; Verstrepen, K.J. Interspecific hybridization as a driver of fungal evolution and Adaptation. *Nat. Rev. Microbiol.* **2021**, *19*, 485–500.
94. Mishra, A.; Forche, A.; Anderson, M.Z. Parasexuality of Candida Species. *Front. Cell. Infect. Microbiol.* **2021**, *11*, 796929. https://doi.org/10.3389/fcimb.2021.796929.
95. Saunders, W.W. Proceedings of learned societies, entomological society. *Ann. Mag. Nat. Hist.* **1842**, *8*, 217–220.
96. Pereira, J. Notice of a Chinese article of the materia medica, called "summer-plant-winter-worm". *Pharm. J. Trans.* **1843**, *2*, 591–595.

97. Lu, D. Western records and studies of the Chinese caterpillar fungus to the beginning of the 20th century. *J. Fungal. Res.* **2014**, *12*, 233–244. https://doi.org/10.13341/j.jfr.2014.0043.
98. Berkeley, M.J. On some entomogenous Sphaeriae. *Lond. J. Bot.* **1843**, *2*, 205–211.
99. Berkeley, M.J. On some entomogenous Sphaeriae. *J. Proc. Linn. Soc. Lond. (Bot.)* **1857**, *1*, 157–159.
100. Saccardo, P.A. Enumeratio pyrenomycetum hypocreaceorum hucusque congitorum systemate carpologico dispositorum. *Michelia* **1878**, *1*, 277–325.
101. Saccardo, P.A. *Sylloge Fungorum Omnium Hucusque Cognitorum*; Sumptibus Auctoris: Patavii, Italy, 1883; Volume 2.
102. Liu, X.-J.; Guo, Y.-L.; Yu, Y.-X.; Zeng, W. Isolation and identification of the anamorph of *Cordyceps sinensis* fungus. *Acta Mycol. Sin.* **1989**, *8*, 35–40.
103. Jackson, D.; Lawson, T.; Villafane, R.; Gary, L. Modeling the structure of yeast MATα1, An HMG-Box motif with a C-terminal helical extension. *Open J. Biophys.* **2013**, *3*, 1–12. https://doi.org/10.4236/ojbiphy.2013.31001.
104. Zheng, Q.; Hou, R.; Zhang, J.-Y.; Ma, J.; Ma, J.-W.; Wu, Z.-S.; Wang, G.-H.; Wang, C.-F.; Xu, J.-R. The MAT locus genes play different roles in sexual reproduction and pathogenesis in *Fusarium graminearum*. *PLoS ONE.* **2013**, *8*, e66980. https://doi.org/10.1371/journal.pone.0066980.
105. Kim, H.-K.; Jo, S.-M.; Kim, G.-Y.; Kim, D.-W.; Kim, Y.-K.; Yun, S.-H. A large-scale functional analysis of putative target genes of mating-type loci provides insight into the regulation of sexual development of the cereal pathogen *Fusarium graminearum*. *PLoS Genet.* **2015**, *11*, e1005486. https://doi.org/10.1371/journal.pgen.1005486.
106. Martin, T.; Lu, S.-W.; van Tilbeurgh, H.; Ripoll, D.R.; Dixelius, C.; Dixelius, C.; Turgeon, B.G.; Debuchy, R. Tracing the Origin of the Fungal a1 Domain Places Its Ancestor in the HMG-Box Superfamily, Implication for Fungal Mating-Type Evolution. *PLoS ONE* **2010**, *5*, e15199. https://doi.org/10.1371/journal.pone.0015199.
107. Ait Benkhali, J.; Coppin, E.; Brun, S.; Peraza-Reyes, L.; Martin, T.; Dixelius, C.; Lazar, N.; van Tilbeurgh, H.; Debuchy, R. A Network of HMG-box Transcription Factors Regulates Sexual Cycle in the Fungus Podospora anserina. *PLoS Genet.* **2013**, *9*, e1003642. https://doi.org/10.1371/journal.pgen.1003642.
108. Yamamoto, A.; Ando, Y.; Yoshioka, K.; Saito, K.; Tanabe, T.; Shirakawa, H.; Yoshida, M. Difference in affinity for DNA between HMG proteins 1 and 2 determined by surface plasmon resonance measurements. *J. Biochem.* **1997**, *122*, 586–594. https://doi.org/10.1093/oxfordjournals.jbchem.a021793. PMID 9348088.
109. Balasubramanian, B.; Lowry, C.V.; Zitomer, R.S. The Rox1 repressor of the *Saccharomyces cerevisiae* hypoxic genes is a specific DNA-binding protein with a high-mobility-group motif. *Mol. Cell Biol.* **1993**, *13*, 6071–6078. https://doi.org/10.1128/mcb.13.10.6071-6078.1993.
110. Zitomer, R.S.; Limbach, M.P.; Rodriguez-Torres, A.M.; Balasubramanian, B.; Deckert, J.; Snow, P.M. Approaches to the study of Rox1 repression of the hypoxic genes in the yeast Saccharomyces cerevisiae. *Methods* **1997**, *11*, 279–288. https://doi.org/10.1006/meth.1996.0422.
111. Kastaniotis, A.J.; Zitomer, R.S. Oxygen Dependent Repression in Yeast. In *Rox1 Mediated Repression*; Advances in Experimental Medicine and Biology; Springer Nature: Cham, Switzerland, 2000; Volume 475, pp. 185–195. https://doi.org/10.1007/0-306-46825-5_18.
112. Kües, U.; Casselton, L.A. The origin of multiple mating types in mushrooms. *J. Cell Sci.* **1993**, *104*, 227–230. https://doi.org/10.1242/jcs.104.2.227.
113. Asante-Owusu, R.N.; Banham, A.H.; Böhnert, H.U.; Mellor, E.J.C.; Casselton, L.A. Heterodimerization between two classes of homeodomain proteins in the mushroom *Coprinus cinereus* brings together potential DNA-binding and activation domains. *Gene* **1996**, *172*, 25–31. https://doi.org/10.1016/0378-1119(96)00177-1.
114. Jacobsen, S.; Wittig, M.; Pöggeler, S. Interaction Between Mating-Type Proteins from the Homothallic Fungus Sordaria macrospora. *Curr. Genet.* **2002**, *41*, 150–158. https://doi.org/10.1007/s00294-002-0276-0.
115. Hancock, S.P.; Cascio, D.; Johnson, R.C. Cooperative DNA binding by proteins through DNA shape complementarity. *Nucleic Acids Res.* **2019**, *47*, 8874–8887. https://doi.org/10.1093/nar/gkz642.
116. Chen, Y.-Q.; Hu, B.; Xu, F.; Zhang, W.; Zhou, H.; Qu, L.-H. Genetic variation of *Cordyceps sinensis*, a fruit-body-producing entomopathogenic species from different geographical regions in China. *FEMS Microbiol. Lett.* **2004**, *230*, 153–158. https://doi.org/10.1016/S0378-1097(03)00889-9.
117. Chen, C.-S.; Hseu, R.-S.; Huang, C.-T. Quality Control of *Cordyceps sinensis* Teleomorph, Anamorph, and Its Products, Chapter 12. In *Quality Control of Herbal Medicines and Related Areas*; Shoyama, Y., Ed.; InTech: Rijeka, Croatia, 2011; pp. 223–238. Available online: www.intechopen.com (accessed on 3 May 2024).
118. Xiao, W.; Yang, J.-P.; Zhu, P.; Cheng, K.-D.; He, H.-X.; Zhu, H.-X.; Wang, Q. Non-support of species complex hypothesis of *Cordyceps sinensis* by targeted rDNA-ITS sequence analysis. *Mycosystema* **2009**, *28*, 724–730.

119. Zhu, J.-S.; Gao, L.; Li, X.-H.; Yao, Y.-S.; Zhou, Y.-J.; Zhao, J.-Q.; Zhou, Y.-J. Maturational alterations of oppositely orientated rDNA and differential proliferations of CG:AT-biased genotypes of *Cordyceps sinensis* fungi and *Paecilomyces hepiali* in natural *C.* sinensis. *Am. J. Biomed. Sci.* **2010**, *2*, 217–238. https://doi.org/10.5099/aj100300217.
120. Barseghyan, G.S.; Holliday, J.C.; Price, T.C.; Madison, L.M.; Wasser, S.P. Growth and cultural-morphological characteristics of vegetative mycelia of medicinal caterpillar fungus *Ophiocordyceps sinensis* G.H. Sung et al. (Ascomycetes) Isolates from Tibetan Plateau (P. R. China). *Intl. J. Med. Mushrooms* **2011**, *13*, 565–581. https://doi.org/10.1615/intjmedmushr.v13.i6.90.
121. Mao, X.-M.; Zhao, S.-M.; Cao, L.; Yan, X.; Han, R.-C. The morphology observation of *Ophiocordyceps sinensis* from different origins. *J. Environ. Entomol.* **2013**, *35*, 343–353.
122. Du, X.-H.; Wu, D.-M.; Kang, H.; Wang, H.-C.; Xu, N.; Li, T.-T.; Chen, K.-L. Heterothallism and potential hybridization events inferred for twenty-two yellow morel species. *IMA Fungus* **2020**, *11*, 4. https://doi.org/10.1186/s43008-020-0027-1.
123. Hėnault, M.; Marsit, S.; Charron, G.; Landry, C.R. The effect of hybridization on transposable element accumulation in an undomesticated fungal species. *eLife* **2020**, *9*, e60474. https://doi.org/10.7554/eLife.60474.
124. Li, X.-Z.; Li, Y.-L.; Wang, Y.-N.; Zhu, J.-S. Translations of mutant repetitive genomic sequences in *Hirsutella sinensis* and changes in secondary structures and functional specifications of the encoded proteins. *Int. J. Mol. Sci.* **2024**, *25*, 11178. https://doi.org/10.3390/ijms252011178.
125. Li, Y.; Yang, R.-H.; Jiang, L.; Hu, X.-D.; Wu, Z.-J.; Yao, Y.-J. rRNA Pseudogenes in Filamentous Ascomycetes as Revealed by Genome Data. *G3-Genes Genom. Genet.* **2017**, *7*, 2695–2703. https://doi.org/10.1534/g3.117.044016.
126. Li, W.-J.; Xia, J.-M.; Li, Q.-P.; Zhang, Z.-Y.; Zhang, W.-W.; Dong, C.-H.; Wei, J.-C.; Liu, X.-Z. Developmental recording of the ghost-moth larvae after ex situ infection by *Ophiocordyceps sinensis*. *Sci. China Life Sci.* **2020**, *63*, 1093–1095. https://doi.org/10.1007/s11427-020-1686-7. Erratum in Sci. China Life Sci. **2023**, 66, 892. https://doi.org/10.1007/s11427-022-2232-5.
127. Holliday, J.; Cleaver, M. Medicinal value of the caterpillar fungi species of the genus *Cordyceps* (Fr.) Link (Ascomycetes). A review. *Int. J. Med. Mushrooms* **2008**, *10*, 219–234. https://doi.org/10.1615/IntJMedMushr.v10.i3.30.
128. Stone, R. Improbable partners aim to bring biotechnology to a Himalayan kingdom. *Science* **2010**, *327*, 940–941. https://doi.org/10.1126/science.327.5968.940.
129. Qin, Q.-L.; Zhou, G.-L.; Zhang, H.; Meng, Q.; Zhang, J.-H.; Wang, H.-T.; Miao, L.; Li, X. Obstacles and approaches in artificial cultivation of Chinese cordyceps. *Mycology* **2018**, *9*, 7–9. https://doi.org/10.1080/21501203.2018.1442132.
